# Psychometric validation of the Ostomy Skin Tool 2.0

**DOI:** 10.7717/peerj.16685

**Published:** 2023-12-18

**Authors:** Gregor Jemec, Nana Overgaard Herschend, Helle Doré Hansen, Amy Findley, Abi Williams, Kate Sully, Tonny Karlsmark, Zenia Størling

**Affiliations:** 1 Department of Dermatology, Roskilde Hospital, Roskilde, Denmark; 2 Clinical Strategies, Coloplast A/S, Humlebæk, Denmark; 3 Patient-Centered Outcomes, Adelphi Values, Bollington, United Kingdom; 4 Copenhagen Wound Healing Centre, Department of Dermatology, Bispebjerg Hospital, København, Denmark

**Keywords:** Peristomal skin, Ostomy, Ostomy skin tool, Psychometric validation

## Abstract

**Background:**

Peristomal skin complications (PSCs) pose a major challenge for people living with an ostomy. To avoid severe PSCs, it is important that people with an ostomy check their peristomal skin condition on a regular basis and seek professional help when needed.

**Aim:**

To validate a new ostomy skin tool (OST 2.0) that will make regular assessment of the peristomal skin easier.

**Methods:**

Seventy subjects participating in a clinical trial were eligible for the analysis and data used for the validation. Item-level correlation with anchors, inter-item correlations, convergent validity of domains, test-retest reliability, anchor- and distribution-based methods for assessment of meaningful change were all part of the psychometric validation of the tool.

**Results:**

A final tool was established including six patient reported outcome items and automatic assessment of the discolored peristomal area. Follow-up with cognitive debriefing interviews assured that the concepts were considered relevant for people with an ostomy.

**Conclusion:**

The OST 2.0 demonstrated evidence supporting its reliability and validity as an outcome measure to capture both visible and non-visible peristomal skin complications.

## Introduction

A compromised skin barrier in the peristomal area can be detrimental to people living with an ostomy. Findings from a recent systematic literature review demonstrated that peristomal skin complications (PSCs) are the most frequent post-operative complication associated with creation of an ostomy (Malik, Lee & Harikrishnan, 2018). The largest multinational survey to date, with data collected from 5,187 subjects across 17 countries, revealed that 88% of the responders reported some level of PSC (Fellows et al., 2021). A recent survey study further supported the importance of PSCs, with 70% of subjects reporting irritated peristomal skin within the ostomy population (Nichols, Goldstine & Inglese, 2019). Due to the high incidence, the negative impact on quality of life, and the associated health-care related costs, PSCs pose a major challenge to people living with an ostomy and society in general (Malik, Lee & Harikrishnan, 2018; Meisner et al., 2012).

Leakage (ostomy output under the adhesive part of the appliance) is a major contributor to development of PSCs. The occurrence of leakage has been shown to significantly correlate with the incidence of PSCs (Voegeli et al., 2020), and an increased leakage frequency has also been reported to correlate with the severity of these skin complications (Porrett et al., 2011). Upon exposure to effluent from an ostomy, the peristomal skin becomes irritated. Common clinical symptoms include itching (67%), bleeding (45%), discoloration (38%), burning (32%), moisture from damage (28%), pain (21%), wounds (11%), and tissue overgrowth (7%) (Voegeli et al., 2020). Collectively, it is of great importance to monitor these symptoms closely to avoid development or progression of an existing PSC.

The Ostomy Skin Tool (OST) is a clinical reported outcome tool designed to assess the condition of peristomal skin in a standardized manner and is considered state-of-the-art approach for this purpose (Andersen et al., 2020). The OST was developed in 2008 and provides a useful evidence-based and validated tool to allow ostomy care nurses to make uniform and qualified decisions regarding evaluation and treatment of PSCs (Martins et al., 2010). The OST consists of two parts: The ‘Assessment’, ‘Intervention’, ‘Monitoring’ (AIM) guide and the DET score. The DET score comprises three standardized domains of abnormal peristomal skin namely discoloration (D), erosion (E), and tissue overgrowth (T) (Martins et al., 2010). For each of these three domains, both the size of the peristomal area affected as well as the severity are evaluated. The area affected is assigned a score between 0 and 3 and the severity is assigned a score between 0 and 2. The total DET score is one single composite score, generated from the three domains, with scores ranging from 0 to 15 (Jemec et al., 2011).

The DET score has been widely used across various clinical studies for evaluation of peristomal skin conditions (Davis et al., 2011; Erwin-Toth, Thompson & Davis, 2012; Kruse & Størling, 2015; Martins et al., 2011; Meisner et al., 2012; Miyo et al., 2017; Porrett et al., 2011; Shiraishi et al., 2020). Despite the advantages of the current DET score, some limitations do exist. Calculation of the DET score is heavily affected by the discoloration domain. If no discoloration is present (*i.e.,* the discoloration area score = 0), then the total DET score = 0 (Jemec et al., 2011). Consequently, there is a risk of not capturing an existing or developing PSC with sensation or visible symptoms in the absence of discolored skin. Moreover, the DET score could in principle be used every day but it requires a trained nurse to administer it. Therefore, the DET score is not applicable for self-assessment by users and will in practice only provide a snapshot of the skin condition. Given that a PSC and particularly discoloration can change rapidly, it is recommended to have a close monitoring program and follow-up between healthcare visits.

Given the limitations of the DET score in the OST, the aim of the current study was to validate a new score for a patient-reported version of the OST. The new tool, referred to as OST 2.0 (Martins et al., 2022), is therefore without the AIM guide and the DET score is replaced with a patient-reported outcome (PRO) questionnaire and an objective assessment of peristomal skin discoloration. The detailed development of the OST 2.0 has been described elsewhere (Martins et al., 2022). The PRO questionnaire includes six items designed to assess the severity of PSCs. Instead of focusing primarily on discoloration, the OST 2.0 has increased focus on sensation symptoms such as pain, itching, and burning alongside capturing signs of compromised skin such as weeping, bleeding, and ulcers. The combination of the PRO and the objective assessment of peristomal skin discoloration form a composite outcome score of OST 2.0, namely the Decision Tree score. Together, the OST 2.0 provides a tool that can be used to monitor the skin closely and with increased sensitivity for evaluating signs related to having peristomal skin complications.

## Materials & Methods

### Study design

Data for the psychometric validation study was obtained from a randomized controlled, open-label, comparative, cross-over, multicenter investigation (Clinical Trial ID: NCT04101318). This investigation was carried out in four countries including United Kingdom (UK), Germany, Italy, and Norway. Subjects were eligible for enrolment if they had a colostomy or ileostomy for at least three months, were at least 18 years old, were able to use an electronic diary (questionnaire), had liquid fecal output, and an existing skin complication in the peristomal area. A total of 79 subjects were enrolled of which 72 completed the investigation. Of these, 70 subjects were eligible to be part of the psychometric analysis population. A small subset of the participants from UK were asked if they were willing to participate in an exit cognitive debriefing interview. Prior to commencing data collection, the investigation was approved by the local ethics committee in each country (UK: 20/LO/0220, Germany: 19-363 and 00012177, the Netherlands: NL71653.068.19, Italy: NP 3841, and Norway: 65025). All subjects provided written informed consent.

### Patient reported outcome (PRO) questionnaire

The new OST 2.0 comprises a PRO questionnaire consisting of six items designed to assess the severity of PSCs (Fig. S1). These items have been identified after qualitative interviews with health care professionals and people with an ostomy. The first three items (Q1, Q2, and Q3) assess the symptoms of bleeding, weeping, and ulcers/sores (visible symptoms) experienced when the subjects changed their product. Subjects living with an ostomy were asked if they were experiencing or not experiencing these symptoms, utilizing a dichotomous response scale.

The remaining three items (Q4–Q6) assess symptoms of itching, pain, and burning (sensation symptoms). For each symptom, the corresponding item asks the subject to rate the severity of the symptom at its worst since the last ostomy product change. These items utilize a 0–10 numerical rating scale ranging from 0 (No symptom) to 10 (Worst possible peristomal skin symptom).

In an exit interview 12 subjects from the UK population participated in 30 min Cognitive Debriefing (CD) interviews conducted by phone.

During the interviews, subjects were asked to discuss and evaluate item relevance, interpretation of items, item response options, and recall periods. Moreover, the subjects were asked whether they thought any important concepts were missing and whether any items should be removed. All interviews were audio-recorded and transcribed verbatim. Qualitative analysis of the verbatim transcripts, was conducted using the computer assisted qualitative analysis software program ATLAS.ti. (Atlas.ti, 2019) PowerBi (Version 2.85.98.0; Microsoft, Redmond, CA, USA) was utilized to generate frequency counts and percentages (based on the proportion of the overall sample) for each item. The CD interviews demonstrated that all items, response options, and recall periods were well understood and considered relevant to the majority of the participating population.

### Peristomal skin image analysis

Image analysis techniques were applied to pictures of peristomal skin taken by the subjects to quantify the total area of discolored skin. Specifically, this was an automated assessment using an algorithm based on artificial intelligence (Andersen et al., 2020). After careful instructions to the participants and step by step guidance in an app (developed for the purpose), images were taken at each ostomy product change, and the total discoloration area was then used as part of the Decision Tree score.

### Decision tree model scoring

The PRO questionnaire and image analysis data were combined in a Decision Tree model to provide an overall score between the score 0–3 representing the severity level of skin complications for each patient. A composite score of 0 represents no treatment required peristomal skin condition and the score of 3 is represents a severe peristomal skin condition. *E.g.* having ulcers or bleeding peristomal skin would be at the highest severity level in the hierarchy and correspond to a Decision Tree score of 3 whereas a pain, itching or burning level below 4 would correspond to a Decision Tree score of 1. A detailed description of the development of the severity categories encompassing the Decision Tree model has been described elsewhere (Martins et al., 2022).

### Anchor measures

For the psychometric evaluation, five anchor measures were included. After review of the literature for gold standard measures to use as anchor measures, it was deemed there were none that were appropriate for use. As such, new items were developed in line with US FDA guidance (FDA, 2009; FDA, 2019) and were qualitatively tested prior to use to ensure patients understood the items as intended. These included the Patient Global Impression of Severity (PGIS), Patient Global Impression of Change (PGIC), Clinician Global Impression of Severity (CGIS), and Clinician Global Impression of Change (CGIC). The DET score was used as anchor measure as well. Although OST 2.0 aims to improve on the DET score, this provided useful information to confirm that the OST 2.0 captures the same concepts as the DET score, but to a more accurate capacity.

For the PGIS anchor, subjects were initially asked whether they had “any skin complications around your stoma today” (Yes/No). If patients answered ‘Yes’, they were then asked to “describe the skin complications around your stoma today”, using a five-point Likert-type scale, with options of ‘very mild’, ‘mild’, ‘moderate’, ‘severe’, and ‘very severe’. These responses were coded from ‘1- very mild’ to ‘5- very severe’ (0 if ‘No’ to the first question). This was asked at both visits.

For the PGIC anchor, subjects were asked “Compared to the beginning of this test period, how have any skin complications around your stoma changed”. Response options used a seven-point Likert-type scale ranging from ‘1 = very much improved, 2 = much improved, 3 = a little improved, 4 = no change, 5 = a little worse, 6 = much worse, 7 = very much worse’. This question was completed at Visit 2 only.

For the CGIS anchor, three versions of the anchor were included. These questions asked about the subject’s overall PSCs, erosion, and discoloration. Firstly, “Does the subject have any PSCs on the peristomal skin today?” (Yes/No). Secondly, “If yes, overall, how would you describe the severity of the subject’s PSCs on the peristomal skin today?” (very severe, severe, moderate, mild, very mild). The responses were coded from ‘1- very mild’ to ‘5- very severe’ (0 if ‘No’ to the first question). This was asked at both visits.

Similarly, there were three CGIC questions asking about changes in the subject’s PSCs. Response options used a seven-point Likert scale ranging from ‘1 = very much improved, 2 = much improved, 3 = a little improved, 4 = no change, 5 = a little worse, 6 = much worse, and 7 = very much worse’. This was asked at Visit 2 only.

The DET score as an anchor measure was calculated by summing all scores given, which resulted in a range of scores from 0 to 15, where higher scores represented more severe symptoms.

### Psychometric validation

Data for the psychometric validation was derived from 70 eligible subjects participating in the clinical investigation (Clinical Trial ID: NCT04101318). Although the study was a cross-over design, only data from the first test period was used (Visit 1 and Visit 2) with exception of the subpopulation eligible for the test-retest evaluation. A detailed overview of the clinical trial is outlined in Fig. S2.

#### Analysis

All analyses were pre-defined in a statistical analysis plan prior to conducting psychometric evaluation and conducted using SAS software (SAS Institute Inc. Cary, NC, USA). The psychometric evaluation was conducted in accordance with European Medicines Agency and US Food & Drug Administration (FDA) best practice guidelines (European Medicines Agency, 2005; FDA, 2009; FDA, 2018; FDA, 2019; FDA, 2022a; FDA, 2022b). The emphasis in a psychometric validation study is on evaluating the magnitude of relationships between variables and the overall pattern of results, rather than on significance testing. Because of this, no adjustment for multiple testing was applied. Where specific thresholds have been proposed for evaluating the results of certain psychometric tests, these have been noted. Where significance tests were used, the threshold for statistical significance was *p* ≤ 0.05 for each test. Where appropriate, results were reported with 95% confidence intervals. All PRO assessments were scored for each subject and summarized. Sociodemographic and clinical variables were obtained and descriptively summarized at baseline in the psychometric analysis population. These variables included gender, age, and type of stoma. For evaluation of the Decision Tree score, only the weekly mean values were investigated. For the PIB score (combination of pain, itching, and burning), it has been indicated for each analysis whether it was performed on weekly mean values alone or weekly mean and weekly maximum values.

#### Item-level correlations with anchors

To evaluate the properties of the individual items, the relationships with anchor measures was explored. Specifically, correlations with the PGIS anchor were explored, and correlations were calculated using data collected at Visit 2, where the PRO data used was from the closest assessment to Visit 2 (provided this was within four days) in the psychometric analysis population. For item 1–3, the point-biserial correlation coefficient was determined due to the use of a dichotomous scale’. For item 4–6, the polyserial correlation coefficient was determined for these severity items. For all correlation coefficients, the following interpretation cut-offs were applied: ‘weak correlation’: *r* < 0.30; ‘moderate correlation’: 0.30 ≤ *r* < 0.50; and ‘strong correlation’: *r* ≥ 0.50 (Cohen, 2013). These thresholds were pre-specified in the statistical analysis plan prior to conducting the psychometric validation.

#### Inter-item correlations

Inter-item correlations were used to explore the relationships among the PRO items. Inter-item correlations were determined using correlation coefficients appropriate for the variables in question between each pair of items at Visit 1. Due to the complexity and variety of the data of interest, using a single type of correlation coefficient would not have been appropriate for all calculations. For item 1–3 (dichotomous scale), the appropriate correlation coefficient was simple matching coefficient, while Pearson’s correlation coefficient was used for the inter-item correlations of item 4–6. Items correlating very highly with one another (*r* ≥ 0.90; indicating over 80% shared variance) were considered to suggest redundancy (Streiner, 2003).

#### Convergent validity of domains

The convergent validity method was applied to evaluate the construct validity and correlation between the different measures (Campbell & Fiske, 1959). Convergent validity was calculated for the PIB score (weekly mean of pain, itching, and burning severity items on a scale from 0–10) and the Decision Tree score using data associated with Visit 2 in the psychometric analysis population (*i.e.,* the weekly score taken over the seven days prior to Visit 2). The measures employed to assess convergent validity included PGIS and the DET score. A polyserial correlation coefficient was calculated, when correlating the PIB score with the PGIS and the Decision Tree score with the PGIS anchor. A Spearman’s correlation coefficient was calculated for the correlation between the PIB score with the DET score and the Decision Tree score with the DET score. The following interpretation cut-offs were applied: weak correlation’: *r* < 0.30; ‘moderate correlation’: 0.30 ≤ *r* < 0.50; and ‘strong correlation’: *r* ≥ 0.50 as suggested for these analyses (Cohen, 2013).

#### Test-retest reliability

Test-retest reliability was used to evaluate the stability of the PIB score and the Decision Tree score in relation to the PGIS, PGIC, CGIS, and CGIC anchor. Moreover, the stability of the weeping, bleeding, and ulcer items were evaluated using the same four anchors. The test-retest reliability measured the degree to which the given score was similar at different points in time in a subset of ‘stable’ patients. A stable subject was defined as a subject with no change in PGIS and CGIS scores from Visit 1 to Visit 2 and similarly no change for the PGIC and CGIC scores from Visit 1 to Visit 2.

The test-retest reliability was determined by calculating the intraclass correlation coefficient (ICC). Specifically, an ICC based on a single measurement, absolute agreement, two-way mixed effects model was used which has been specifically recommended for use in test-retest reliability analyses (Koo & Li, 2016). A key assumption of this variant is that the two time points at which scores are measured are the only time points of interest, rather than being sampled from a wider population of possible time points. The absolute agreement component is specified to incorporate systematic differences between scores at each timepoint. This ICC variant is mathematically equivalent to the ICC (2, 1) (Koo & Li, 2016). The following cut-offs were employed to interpret ICC values: ICC < 0.5 indicated poor reliability, ICC values between 0.5 and 0.75 indicated moderate reliability, ICC values between 0.75 and 0.9 indicated good reliability, and ICC values greater than 0.90 indicated excellent reliability (Bobak, Barr & O’Malley, 2018).

#### Known-groups analysis

The PIB score and the Decision Tree score were evaluated in patients who differed on variables hypothesized to influence the construct of interest. The magnitude of differences in scores characterized the degree to which the PIB score/Decision Tree score could distinguish among groups hypothesized a priori to be clinically distinct. Known-groups comparisons were assessed using data from the measurement period associated with Visit 2 in the psychometric analysis population. The known-groups were defined for the PGIS anchor by asking the following question: ‘Do you have any complications around your stoma today? If yes, overall, how would you describe the skin complications around your stoma today’. This led to three defined groups: ‘Group 1- no (reference)’, ‘Group 2- very mild or mild’, and ‘Group 3- moderate, severe, or very severe’.

The magnitude of the differences was evaluated using between-group effect size estimates, calculated using the pooled standard deviation (SD) as the denominator, and based against the reference group as defined. The following cut-offs were used to interpret the magnitude of each effect size (ES): small change (ES = 0.20), moderate change (ES = 0.50), and large change (ES = 0.80) (Cohen, 2013). The statistical significance of differences in scores between groups was also calculated using the *F*-test of one-way ANOVAs with a significance level of *p* ≤ 0.05.

#### Ability to detect change

The ability of a score to detect change over time was assessed using data from the measurement periods associated with Visit 1 and Visit 2 in the psychometric analysis population. To investigate the ability of the PIB score to detect change, subjects were grouped according to the PGIC anchor and categorized into ‘Improved’, ‘Stable’, and ‘Worsened’ groups as follows: ‘Improved’ (very much improved, much improved, or a little improved at Visit 2), ‘Stable’ (no change at Visit 2), and ‘Worsened’ (a little worse, much worse, or very much worse at Visit 2). For the Decision Tree score, the same groups were defined using the CGIS anchor instead. For both domains, the frequency and percentage of subjects in each category were summarized, and the mean change scores for each group from Visit 1 to Visit 2 were listed alongside the SD. The mean change scores were compared between the three groups, and one-way ANOVA *F*-test was employed to evaluate the statistical significance of any differences in change scores between each group.

#### Anchor-based methods for assessing meaningful change

Anchor-based methods were conducted to establish the level of change which could be considered meaningful for the domains. For this analysis, both PIB weekly mean and PIB weekly maximum scores were assessed alongside the Decision Tree score. The anchor-based analyses were performed in the psychometric analysis population using data from Visit 1 and Visit 2. The suitability of proposed anchors was tested using a polyserial correlation coefficient to establish the relationship between the anchor categories and change in domain scores. Anchors with correlations of *r* < 0.3 were not taken forward for analysis (Revicki et al., 2008).

For PIB weekly mean and PIB weekly maximum, PGIC was the only anchor demonstrating a sufficient polyserial correlation coefficient. Thus, the PGIC anchor was used to define groups of patients who had experienced improvement or no change. For the Decision Tree score, the CGIS anchor was used instead due to a sufficient polyserial correlation coefficient, and patient groups were again defined as experiencing either improvement or no change. Subjects with worsened skin complications were excluded from this analysis. The groupings based on the PGIC/CGIS anchor were as follows: ‘Improved’ (very much improved, much improved, or a little improved at Visit 2) and ‘Stable’ (no change at Visit 2).

The within-group mean change scores evaluated the minimal important change (MIC) within groups. The mean change in domain score was calculated for patients classified according to the PGIC anchor (PIB weekly mean and PIB weekly maximum) and the CGIS anchor (Decision Tree score). The MIC estimate was derived using each groups’ mean change scores.

The between-group differences in mean change scores evaluated the minimal important difference (MID) between groups. This analysis informed between-group MID estimates, and the mean change in domain scores was calculated for patients classified as above according to the PGIC anchor (PIB weekly mean and PIB weekly maximum) and the CGIS anchor (Decision Tree score). The MID estimate was defined as the difference in mean change score between these groups.

#### Distribution-based methods for assessing meaningful change

A distribution-based approach was employed, and these methods consisted of computing the SD and the standard error of measurement (SEm) (Wyrwich, Tierney & Wolinsky, 1999). This distribution-based approach involved calculating 0.5 of the SD at the Visit 2 measurement. The SEm was calculated as the SD at the Visit 2 measurement period multiplied by the square root of one minus the reliability of the score at baseline. Therefore, the SEm was equivalent to 0.5 SD when the reliability equaled 0.75 and decreased as reliability increased. The ICC values calculated based on the PGIS anchor between Visit 1 and Visit 2 were used for the reliability of scores when determining the SEm. A value of 1 SEm was used as the estimate of the responder threshold.

## Results

### Sociodemographic profile

The psychometric analysis sample was comprised of a total of 70 subjects living with an ostomy. There was an even distribution between females (51%) and males (49%), and the population had a mean age of 55.3 years (Table 1). There was a larger proportion of subjects with an ileostomy (80%) compared to subjects with a colostomy (20%) (Table 1), which was expected based on the inclusion criteria for the clinical investigation.

**Table 1:** Sociodemographic profile of subjects. The psychometric analysis population was comprised of 70 subjects living with an ostomy. Data shows distribution of samples according to gender, age, and type of ostomy.

**Gender**	
Female (n, %)	36 (51%)
Male (n, %)	34 (49%)
**Age**	
Mean (min; max)	55.3 (19;80)
**Type of ostomy**	
Colostomy (n, %)	14 (20%)
Ileostomy (n, %)	56 (80%)

### Item-level correlations with anchors

The severity items were correlated with the PGIS anchor. Table 2 depicts the correlation coefficients for the six items within the PRO.

**Table 2:** Item-level correlations. The correlations of the six items were determined by calculating the relevant correlation coefficient based on the PGIS anchor (*n* = 59). Cut-offs applied were ‘weak correlation’: *r* < 0.30; ‘moderate correlation’: 0.30 ≤ *r* < 0.50; and ‘strong correlation’: *r* ≥ 0.50.

**Item**	**Type of correlation coefficient**	**r**
1–Bleeding	Point-biserial	0.266
2–Weeping	Point-biserial	0.431
3–Ulcers/sores	Point-biserial	0.633
4–Itching (severity)	Polyserial	0.457
5–Pain (severity)	Polyserial	0.442
6–Burning (severity)	Polyserial	0.468

Based on the applied cut-off values, five out of six items demonstrated a moderate or strong correlation with the PGIS anchor. The item regarding bleeding (item 1) showed a 0.266 correlation coefficient, which was therefore classified as a weak correlation with the given anchor.

### Inter-item correlations

To explore how the items could be grouped into domains, the inter-item correlations were examined among the items assessing itching severity, pain severity, and burning severity (item 4, 5, and 6). As depicted in Table 3, the itching severity item showed a moderate correlation with both the pain severity item (*r* = 0.668) and burning severity item (*r* = 0.600). In addition, the pain severity and burning severity items were shown to correlate well (*r* = 0.800) (Table 3). Moreover, no redundancy (*r* ≥ 0.9) was observed. Collectively, these data support combining the pain, itching, and burning severity items into a single domain; referred to as the PIB score.

**Table 3:** Inter-item correlations for severity items. The Pearson’s correlation coefficient was determined for the itching severity, pain severity, and burning severity items. *r* ≥ 0.9 indicated redundancy.

	**4-Itching**	**5-Pain**	**6-Burning**
**Itching**	N/A	–	–
**Pain**	0.668	N/A	–
**Burning**	0.600	0.800	N/A

The weeping, bleeding, and ulcer/sore items were also subject to inter-item correlation analysis. All correlation among those items were poor; thus, the weeping, bleeding, and ulcer/sore items were not combined into a domain score but kept as single items (Files S8 and S9).

### Convergent validity of domains

In addition to the composite outcome score of the OST 2.0, namely the Decision Tree score, the PIB domain was also taken through for further validation at the domain level. The PGIS and DET score were the two anchors used for assessing convergent validity of the two domains. When determining the polyserial correlation coefficient, it was evident that the PIB score correlated moderately with the PGIS anchor (*r* = 0.436), while the Decision Tree correlated strongly with this anchor measure (*r* = 0.560) (Table 4). In addition, evaluation of the Spearman’s correlation coefficient revealed a weak correlation between the PIB score and the DET score (*r* = 0.241) alongside a strong correlation between the Decision Tree score and the DET score (*r* = 0.592) (Table 4).

**Table 4:** Convergent validity of domains. The polyserial correlation coefficient was determined for correlation of the PIB score (weekly mean) and the PGIS anchor (*n* = 60) and for correlation of the Decision Tree score and the PGIS anchor (*n* = 57). The Spearman’s correlation coefficient was determined for correlation of the PIB score and the DET score (*n* = 58) and for correlation of the Decision Tree score and the DET score (*n* = 55). Cut-offs applied were ‘weak correlation’: *r* < 0.30; ‘moderate correlation’: 0.30 ≤ *r* < 0.50; and ‘strong correlation’: *r* ≥ 0.50.

	**PIB score**	**Decision Tree score**
**PGIS**	0.436	0.560
**DET score**	0.241	0.592

### Test-retest reliability

The ICC can be interpreted as the correlation between repeatedly measured scores within subjects, where higher values indicate greater stability in scores. The test-retest reliability was investigated for the PIB score (weekly mean) and the Decision Tree score. The PIB score demonstrated good reliability when using the CGIS anchor (ICC = 0.871) and the PGIC anchor (ICC = 0.785) (Table 5). Moreover, the PIB score showed moderate reliability when using the PGIS anchor (ICC = 0.673) and CGIC anchor (ICC = 0.753) (Table 5). The Decision Tree score showed good reliability when using the PGIS anchor (ICC = 0.805) and the PGIC anchor (ICC = 0.823) alongside moderate reliability when employing the CGIS anchor (ICC = 0.735) and the CGIC anchor (ICC =0.735) (Table 5). Collectively, these data provide good evidence of test-retest reliability for both domain scores.

**Table 5:** Test-retest reliability of weekly mean domain scores between the two visits. The test-retest reliability of the PIB score (weekly mean) and Decision Tree score were evaluated by calculating the intraclass correlation coefficient (ICC). Data is listed with 95% confidence intervals displayed in brackets. For the number of subjects, data is displayed as n (PIB score)/n (Decision Tree score). The following cut-offs were applied: ICC < 0.5 indicated poor reliability, ICC values between 0.5 and 0.75 indicated moderate reliability, ICC values between 0.75 and 0.9 indicated good reliability, and ICC values greater than 0.90 indicated excellent reliability.

**Anchor**	**n**	**ICC–PIB score**	**ICC–decision tree score**
PGIS	13/12	0.673 (−0.100, 0.901)	0.805 (0.292, 0.944)
CGIS	21/20	0.871 (0.686, 0.947)	0.735 (0.326, 0.896)
PGIC	34/31	0.785 (0.573, 0.892)	0.823 (0.637, 0.915)
CGIC	31/30	0.753 (0.455, 0.884)	0.735 (0.449, 0.874)

When evaluating the bleeding item, strong ICC scores when stable patients were defined using the PGIS, PGIC, and CGIC anchors (ICC range: 0.758–0.804) were demonstrated, whereas for the CGIS anchor test-retest results were poor (ICC = 0.314) (Table 6). Similarly, the weeping item exhibited strong ICC scores when stable patients were defined using the PGIS, PGIC, and PGIC anchors (ICC range: 0.734–0.860), while this item also showed a poor correlation with the CGIS anchor (ICC = 0.419) (Table 6). Finally, test-retest results were strong for the ulcers/sores item when stable patients were defined using the PGIS anchor (ICC = 0.853) and moderate test-retest reliability when stability was defined using the CGIS, PGIC, and CGIC (ICC range: 0.642–0.745) (Table 6).

**Table 6:** Test-retest reliability of bleeding, weeping, and ulcers/sores items. The test-retest reliability the bleeding, weeping, and ulcers/sores items were evaluated by calculating the intraclass correlation coefficient (ICC). Data is listed with 95% confidence intervals displayed in brackets. The number of subjects used for the analysis is displayed (n). The following cut-offs were applied: ICC < 0.5 indicated poor reliability, ICC values between 0.5 and 0.75 indicated moderate reliability, ICC values between 0.75 and 0.9 indicated good reliability, and ICC values greater than 0.90 indicated excellent reliability.

**Anchor**	**n**	**ICC–Bleeding**	**ICC–Weeping**	**ICC–Ulcers/sores**
PGIS	13	0.758 (0.244, 0.925)	0.860 (0.535, 0.958)	0.853 (0.503, 0.955)
CGIS	21	0.314 (−0.734, 0.724)	0.419 (−0.456, 0.766)	0.645 (0.153, 0.854)
PGIC	34	0.804 (0.607, 0.902)	0.810 (0.623, 0.905)	0.745 (0.487, 0.873)
CGIC	31	0.801 (0.584, 0.904)	0.734 (0.449, 0.871)	0.642 (0.262, 0.827)

### Known-groups analysis

The known-groups analysis of the PIB score and the Decision Tree score was evaluated by comparing groups defined based on the PGIS anchor. When evaluating the differences in PIB mean scores between the three groups, Group 1 (reference) showed a mean score of 1.5, while group 2 and 3 demonstrated a mean score of 1.9 and 3.6, respectively (Table 7). Thus, there were monotonically increasing scores across groups, as hypothesized, with a statistically significant difference in mean scores between the groups (*p* = 0.003). Compared to the reference population (Group 1), this corresponded to a small between-groups ES for Group 2 (ES = 0.24) and a large between group ES for Group 3 (ES = 1.04) (Table 7). For the Decision Tree score, a mean score of 1.5 was shown for Group 1 (reference), while Group 2 and Group 3 demonstrated a mean score of 1.8 and 2.7, respectively (Table 7). Thus, again there were monotonically increasing scores across groups, with statistically significant differences between the groups (*p* < 0.001). When comparing to the reference group, a small between-groups ES was found for Group 2 (ES = 0.30), and a large between group ES for Group 3 (ES = 1.49; Table 7).

**Table 7:** Known-groups analysis of the domain scores. Known-groups analysis was investigated for the PIB score (weekly mean) and for the Decision Tree score. Subjects were divided into three groups depending on presence and severity of peristomal skin complications. Using the PGIS anchor, the between group effect sizes (ES) were estimated using the pooled standard deviation (SD) based on the reference group (Group 1). The following cut-offs were applied: small change (ES = 0.20), moderate change (ES = 0.50), and large change (ES = 0.80). The *F*-test of one-way ANOVA was used to determine the statistical significance of differences in scores between groups. *p* ≤ 0.05 was considered significant.

**Grouping variable**	**n**	**Mean score (SD)**	**Between groups effect size**	**Between groups *p*-value**
**PIB score**				
Group 1 - No (reference)	31	1.5 (1.58)	–	0.003
Group 2 - Very mild or Mild	14	1.9 (1.40)	0.24	–
Group 3 - Severe or Very severe	15	3.6 (2.56)	1.04	–
**Decision tree score**				
Group 1 - No (reference)	30	1.5 (0.88)	–	<0.001
Group 2 - Very mild or mild	12	1.8 (0.84)	0.30	–
Group 3 - Severe or very severe	15	2.7 (0.56)	1.49	–

### Ability to detect change

The ability of the PIB score to detect change was investigated by using the PGIC anchor to define change groups, while the ability of the Decision Tree score to detect change was evaluated by comparison with the CGIS anchor. The mean change score was assessed for the three groups of subjects. For the PIB score, the change score was negative (indicating an improvement in score) in the improved group (mean change score = −1.6) with a larger change compared to the stable population (mean change score = −0.3) (Table 8). The worsened group displayed a positive change score (mean change score = 0.3) compared to the stable group (mean change score = −0.3) (Table 8); thus, the PIB score (weekly mean) did fluctuate in accordance with the pre-defined patient groups. Finally, the one-way ANOVA F-test demonstrated a statistically significant difference in change scores between the subject groups (Table 8).

**Table 8:** Ability to detect change of domain scores. The ability of the PIB score (weekly mean) to detect change was evaluated by use of the PGIC anchor, while the ability of the Decision Tree score to detect change was investigated by comparison with the CGIS anchor. Subjects were divided into three groups depending on their progression from Visit 1 to Visit 2. These groups included ‘Improved’ subjects (very much improved, much improved, or a little improved at Visit 2), ‘Stable’ subjects (no change at Visit 2), and ‘Worsened’ subjects (a little worse, much worse or Very much worse at Visit 2). The mean change score was determined. One-way ANOVA F-test was used to calculate potential statistical significance of differences in change scores between groups.

**Grouping variable**	**n**	**Mean change score (SD)**	**Between groups *p*-value**
**PIB score**			
Improved	14	−1.6 (1.75)	–
Stable	34	−0.3 (1.53)	–
Worsened	6	0.3 (2.41)	0.026
**Decision tree score**			
Improved	25	−0.4 (0.75)	–
Stable	20	−0.1 (0.85)	–
Worsened	10	0.1 (0.92)	0.246

For the Decision Tree score, a larger negative change in mean score was shown for the improved group (mean change score = −0.4) compared to the stable one (mean change score = −0.1). Moreover, the worsened group demonstrated a positive change in mean score (mean score = 0.1) compared to the stable group (mean change score = −0.1) (Table 8). Although no statistically significant difference between the groups was found (*p* = 0.246), the Decision Tree score also fluctuated in accordance with the pre-defined patient groups. Combined, both domain scores demonstrated an ability to detect change.

### Anchor-based methods of score interpretation

To establish an estimate for a meaningful change in domain score, a correlation between the anchor and the change in domain scores of *r* > 0.3 was required. As depicted in Table 9, the PGIC anchor correlated sufficiently with the change in PIB weekly mean score (*r* = 0.454) and the PIB weekly maximum score (*r* = 0.422). When a subject improved from Visit 1 to Visit 2, the MIC of the PIB weekly mean score and the PIB weekly maximum score was 1.6 units and 2.5 units, respectively (Table 9). When comparing between subjects, the MID value for the PIB weekly mean score was a 1.3-point reduction, while MID for the PIB weekly maximum score was a 1.6-point reduction (Table 9). For the Decision Tree score, the CGIS anchor was used instead of the PGIC anchor due to a sufficient correlation with the change in domain score (*r* = 0.31). The MIC value for the Decision Tree was a 0.52-point reduction, while the MID value was a 0.4-point reduction (Table 9).

**Table 9:** Meaningful change estimates for domain scores. Meaningful change estimates for the PIB weekly mean and PIB weekly maximum domains were calculated using the PGIC anchor. For the Decision Tree score, the CGIS anchor was used instead. The correlation between the anchor and the change in domain score was determined by calculating polyserial correlation coefficient. Subjects were divided into groups based on their progression from Visit 1 to Visit 2. According to the anchor point used, the groups were defined as ‘Improved’ (very much improved, much improved, or a little improved at Visit 2) and ‘Stable’ (no change at Visit 2). Meaningful change estimates were determined within subjects (minimal important change) and between groups (minimal important difference). Data is displayed as the mean change score / mean difference score with the 95% confidence interval being displayed in brackets for each mean value.

**Grouping variable**	**n**	**Anchor correlation**	**Within subjects** **(MIC)**	**Between subjects** **(MID)**
**PIB score (weekly mean)**				
Improved	14	0.45	−1.6 (−2.50, −0.78)	
Stable	34	–	−0.3 (−0.86, 0.24)	−1.3 (−2.35, −0.30)
**PIB score (weekly maximum)**				
Improved	14	0.42	−2.5 (−3.90, −1.24)	
Stable	34	–	−0.9 (−1.77, −0.06)	−1.6 (−3.24, −0.08)
**Decision tree score**				
Improved	11	0.31	−0.52 (−1.03, −0.00)	
Stable	20	–	−0.10 (−0.48, 0.28)	−0.4 (−1.05, 0.23)

**Notes.**

Abbreviations MIC minimal important change MID minimal important difference

### Distribution-based methods of score interpretation

In addition to the anchor-based methods, distribution-based methods were also used to determine a meaningful change for the domain scores. These methods aimed to identify the smallest amount of change which exceeded measurement errors. Thus, the distribution-based estimates, in the form of 0.5 SD and the SEm, were calculated for the domain scores. For PIB weekly mean, the distribution-based methods suggested a point reduction exceeding 1.13 to be meaningful (Table 10). For the PIB weekly maximum, a point reduction exceeding 1.53 was suggested as a meaningful change (Table 10). Finally, a point reduction exceeding 0.42 was proposed as a meaningful change for the Decision Tree score (Table 10).

**Table 10:** Distribution-based estimates for PIB weekly mean and PIB weekly maximum.

**Domain scores**	**n**	**$ \frac{1}{2} $ SD**	**SEm (ICC)**
PIB score (weekly mean)	64	0.98	1.13
PIB score (weekly maximum)	64	1.21	1.53
Decision Tree score	64	0.47	0.42

**Notes.**

The distribution-based estimates were determined for the PIB weekly mean and PIB weekly maximum domain. The estimates were 0.5 of the SD and the SEm.

Abbreviations SD standard deviation SEm standard error of measurement ICC intraclass correlation coefficient

## Discussion

The OST 2.0 was designed to evaluate the severity of PSCs within the ostomy population, and the Decision Tree score offers a simple and evidence-based categorization of PSC severity (Martins et al., 2022). This study presents the psychometric validation of the OST 2.0. CD interviews ensured that the concepts comprising the PRO were relevant and of interest for people living with an ostomy, and the psychometric analysis sample was considered representative of the ostomy population. Three domain scores were validated, namely PIB (weekly mean), PIB (weekly maximum), and the Decision Tree score. The reason for including two versions of the PIB score was to accommodate for comparison of subjects with similar or different device changing patterns.

Despite the continuous development of improved ostomy devices, people living with an ostomy continue to experience challenges with PSCs (Fellows et al., 2021). Within the ostomy care field, other psychometric validated tools do exist including among others the Ostomy-Q (Nafees, Rasmussen & LL, 2017), the Ostomy Leak Impact Tool (Nafees et al., 2018), the Ostomy Adjustment Inventory (Simmons, Smith & Maekawa, 2009), the Ostomy Adjustment Scale (Zhang et al., 2015), the Stoma-Quality-of-Life (Prieto, Thorsen & Juul, 2005), the City of Hope Quality of Life-Ostomy Questionnaire (Grant et al., 2004), the Ostomy Self-Care Index (Villa et al., 2019), and the Caregiver Contribution to Self-Care in Ostomy Patient Index (Villa et al., 2019). However, none of these instruments specifically focus on evaluating the severity of PSCs.

A review by Haugen & Ratliff (2013) compared some existing, yet not psychometric validated, tools available for assessing PSCs in the ostomy care field. Amongst those four tools, the OST (Martins et al., 2010) was the only one containing a scoring system and was referred to as a standardized approach for determining the condition of peristomal skin. Although the OST was validated to some degree (Jemec et al., 2011), the tool was not subject to an actual psychometric validation. For this reason, it was impossible to directly compare the OST and OST 2.0, as the validation processes measured different performance parameters. However, the OST 2.0 has clear advantages including no need for training prior to using the tool, increased sensitivity, and the ability to closely monitor the skin. The DET score, which is the outcome of the OST, requires trained personnel to administer it. As such, the DET score does not allow for self-assessment by the users, meaning they cannot monitor the changes in their skin condition closely.

To be fit for purpose, an instrument should demonstrate psychometric properties including validity, reliability, and responsiveness to change (Mouelhi et al., 2020). The Ostomy Complication Severity Index (Pittman et al., 2014) is a psychometric validated tool for assessing incidence and severity of ostomy complications in recently operated patients. Although it assesses a few PSC symptoms like pain and bleeding, this instrument focuses on early post-operative complications and may not be relevant for the majority of the ostomy population. Moreover, the Ostomy Complication Severity Index does not provide estimates of clinically meaningful changes (Pittman et al., 2014); thus, limiting its interpretation of score changes. As such, the OST 2.0 is, to the best of our knowledge, the first psychometrically validated PRO instrument specifically focusing on assessing visible and sensation symptoms of PSCs.

Overall, the OST 2.0 instrument demonstrated good correlations with the anchor measures at item level, and inter-item correlations were therefore subsequently evaluated; revealing that pain, itching, and burning severity items could be mapped together. Thus, generating the possibility of using the PIB score as a second composite score in addition to the Decision Tree score, which currently is the outcome score of the OST 2.0.

Concept elicitation work performed during development of the OST 2.0 (Martins et al., 2022) underlined the importance of the pain, itching, burning, weeping, bleeding, and ulcer items for people with an ostomy. The association between itching and pain has previously been reported (Davidson & Giesler, 2010) alongside a demonstration of pain, itching, and burning sensations being common co-existing symptoms for patients with chronic venous insufficiency (Duque et al., 2005). Thus, it was found that the correlations evaluated provided support for the pain, itching, and burning items to be combined together to form a domain score in the ostomy population. In contrast, the weeping, bleeding, and ulcer/sore items were not found to be closely related with low inter-item correlations with each other. Consequently, the weeping, bleeding, and ulcer/sore will be evaluated individually.

When evaluating convergent validity of the PIB domain, a moderate correlation with the PGIS anchor was found, while its correlation with the DET score was weak. These data underlined that there was conformity in what the PIB score measures and what people with an ostomy were experiencing. The weak correlation with the DET score was expected as it further supports the difference between what the DET score measures and how people with an ostomy experience sensation symptoms in the peristomal area. The Decision Tree score demonstrated a strong correlation with the DET score, which could partially be due to the incorporation of peristomal image analysis and subsequent quantification of the discolored area in this domain. Moreover, this correlation could also reflect that the visible signs of PSCs (weeping, bleeding, and ulcer/sores) are an integrated part of the Decision Tree score. As the discoloration domain is strongly impacting the outcome of the DET score (Jemec et al., 2011), the OST 2.0 has the advantage of incorporating both discoloration area and the severity levels of sensation symptoms, which are absent in OST.

The OST 2.0 demonstrated good stability based on the test-retest reliability assessment. This evaluation was conducted to evaluate the degree to which the PIB (weekly mean) score and the Decision Tree score were similar over time in a subset of subjects (defined as having stable peristomal skin according to anchor points). In general, test-retest reliability findings should be interpreted in consideration of the ability to detect change findings, as good test-retest reliability can be the artefact of a score being unable to detect change. If an instrument like the OST 2.0 is intended to measure a change in patients over time, it is crucial that the tool is responsive to change (Mouelhi et al., 2020). This means that the domain scores must fluctuate in accordance with true change to possess the ability to detect change. The fluctuations of the PIB score and the Decision Tree score between the pre-defined ‘improved’, ‘stable’, and ‘worsened’ patient groups underlined that these domains were responsive to change, and the test-retest results were therefore not an artefact.

The ability to detect change is an inevitable prerequisite to subsequently determine the meaningful change of a score. Positioning the magnitude of a given clinical change into a meaningful context can often be challenging and a statistical analysis for interpreting the outcome of a clinical score should not stand alone (Juniper et al., 1994; Mouelhi et al., 2020). According to the US FDA guidance on interpretation of PRO results (McLeod et al., 2011), distribution-based methods can provide supportive evidence of meaningful change, but the anchor-based methods should be considered the primary approach for obtaining these thresholds. In this study, the anchor-based methods suggested a 1.3-point reduction for PIB score (weekly mean), a 1.6-point reduction for PIB score (weekly maximum), and a 0.4-point reduction for the Decision Tree score as a meaningful change. These estimates may be useful *e.g.*, if these domain scores are to be used in clinical trials for evaluating the performance of a new ostomy device. Importantly, one must keep the relatively large SD-values of the meaningful estimates in mind, when interpreting MID values in clinical investigations. Of note, the US FDA supports the use of PRO instruments to measure primary or secondary safety and/or performance endpoints (FDA, 2020); further underlining the potential in using one of the composite scores, *i.e.,* the Decision Tree score or the PIB score, in clinical investigations.

## Limitations

Despite the fact that the psychometric analysis sample was broad and representative of the end user population, the study did encompass some limitations. Specifically, the sample size for (70 subjects for the psychometric validation) could have been larger although similar sample sizes have been used for other tools *e.g.*, the Ostomy Complication Severity Index (Pittman et al., 2014). The potential concerns regarding sample size were more pronounced in analyses where subjects were subdivided into smaller groups. For instance, the ‘improved’ groups for determining estimates of meaningful change (MIC/MID) was relatively small. Moreover, the meaningful change estimates were determined with relatively large SD intervals. Based on this, additional evaluations may be needed to further explore these estimates for use in clinical investigations, and it has been suggested elsewhere that full confidence in a given MID value evolves over time (Revicki et al., 2006).

PGI/CGI items were developed specifically for use as anchor measures in the psychometric evaluation of the OST 2.0 due to lack of existing measures that would be appropriate for these analyses. However, the PGI/CGI items were qualitatively tested prior to use to ensure patients understood the items as intended, and the items were developed in line with FDA guidance. Additionally, comparisons of the DET and OST 2.0 scores were drawn to confirm that the new OST 2.0 measures the same concepts as the DET score but with the aim of being more sensitive.

Finally, different types of correlations were used in the analyses based on the type of data included. Although this follows guidelines it may be harder to draw comparisons across correlations. Factor analysis was not performed to evaluate dimensionality due to sample size limitations and the complexity of the instrument.

## Conclusions

This study presents the psychometric validation of the OST 2.0 instrument. The evidence provided support that OST 2.0 is reliable and valid for assessing severity of PSCs. Unlike the OST, this new tool enables close monitoring and captures subjects with PSC even in the absence of discolored peristomal skin. The Decision Tree score and PIB score both have great potential as a primary endpoint in clinical investigations. However, the meaningful change estimates should be interpreted with caution due to the sample size and the SD intervals of the estimates. Collectively, the OST 2.0 instrument provides a standardized, objective, sensitive, and easy-to-use approach for closely assessing changes in peristomal skin conditions over time, which can capture both visual and non-visual symptoms of PSC.

## Supplemental Information

10.7717/peerj.16685/supp-1 Supplemental Information 1 Patient questionnaire

10.7717/peerj.16685/supp-2 Supplemental Information 2 Study design

10.7717/peerj.16685/supp-3 Supplemental Information 3 Skin area visit 3

10.7717/peerj.16685/supp-4 Supplemental Information 4 Skin Discolouration score

10.7717/peerj.16685/supp-5 Supplemental Information 5 Skin area visit 1

10.7717/peerj.16685/supp-6 Supplemental Information 6 Skin area visit 2

10.7717/peerj.16685/supp-7 Supplemental Information 7 DET Score

10.7717/peerj.16685/supp-8 Supplemental Information 8 Case Report Form (annotated)

10.7717/peerj.16685/supp-9 Supplemental Information 9 Baseplate Change scoring

10.7717/peerj.16685/supp-10 Supplemental Information 10 Randomisation

## Additional Information and Declarations

### Data Availability

The following information was supplied regarding data availability: The raw measurements and an annotated Case Report Form are available in the Supplementary Files.
