# Supplementary material for: Psychometric validation of the Ostomy Skin Tool 2.0
Source: PeerJ. 2023 Dec 18;11:e16685. doi: 10.7717/peerj.16685 (PMC10734405; doi:10.7717/peerj.16685)
Supplement: Supplemental Information 1 [file peerj-11-16685-s001.pdf]

Modified ostomy skin tool

Patient reported outcome

Coloplast

Patient questionnaire

Patient number: _ _ _ _ _ _ _ **Date:** _ _/_ _/_ _ _ _

<u>Instructions</u>

These questions should be completed by the patient themselves. *The following questions ask about the* ***skin complications*** *you experience* ***around your stoma*** *(from the stoma site to the edge of the stoma bag adhesive). Please answer each question thinking about* ***right now when changing your product.***

Question 1. Do you experience any **bleeding from the skin** around your stoma **right now** when changing your product? (tick one box only)

☐	☐
Experiencing	Not experiencing

Question 2. Once you have cleaned and dried the skin, do you still experience any **weeping or moisture on the skin** around your stoma **right now** when changing your product? (tick one box only)

☐	☐
Experiencing	Not experiencing

Question 3. Are you experiencing any **ulcers or sores** around your stoma **right now** when changing your product? (tick one box only)

☐	☐
Experiencing	Not experiencing

<u>Instructions</u>

The following questions ask about the ***skin complications*** *you experience* ***around your stoma*** *(from the stoma site to the edge of the stoma bag adhesive). Please answer each question thinking about the period* ***since you last changed your product until now****.*

Question 4. Please rate on a scale from 0-10 how **itchy** the skin around your stoma has been <u>at its worst</u> since you last changed your product (tick one box only)

☐	☐	☐	☐	☐	☐	☐	☐	☐	☐	☐
0<br>No itch	1<br>Very mild itch	2	3	4	5	6	7	8	9	10<br>Worst possible peristomal skin itch

Patient questionnaire

Patient number: _ _ _ _ _ _ _ **Date:** _ _/_ _/_ _ _ _

Question 5. Please rate on a scale from 0-10 how **painful** the skin around your stoma has been at its worst since you last changed your product (tick one box only)

☐	☐	☐	☐	☐	☐	☐	☐	☐	☐	☐
0<br>No pain	1<br>Very mild pain	2	3	4	5	6	7	8	9	10<br>Worst possible peristomal skin pain

Question 6. Please rate on a scale from 0-10 any **burning** feelings from the skin around your stoma at its worst since you last changed your product (tick one box only)

☐	☐	☐	☐	☐	☐	☐	☐	☐	☐	☐
0<br>No burning	1<br>Very mild burning	2	3	4	5	6	7	8	9	10<br>Worst possible peristomal skin burning
