# Supplementary figures and images for: Psychometric validation of the Ostomy Skin Tool 2.0
Source: PeerJ. 2023 Dec 18;11:e16685. doi: 10.7717/peerj.16685 (PMC10734405; doi:10.7717/peerj.16685)

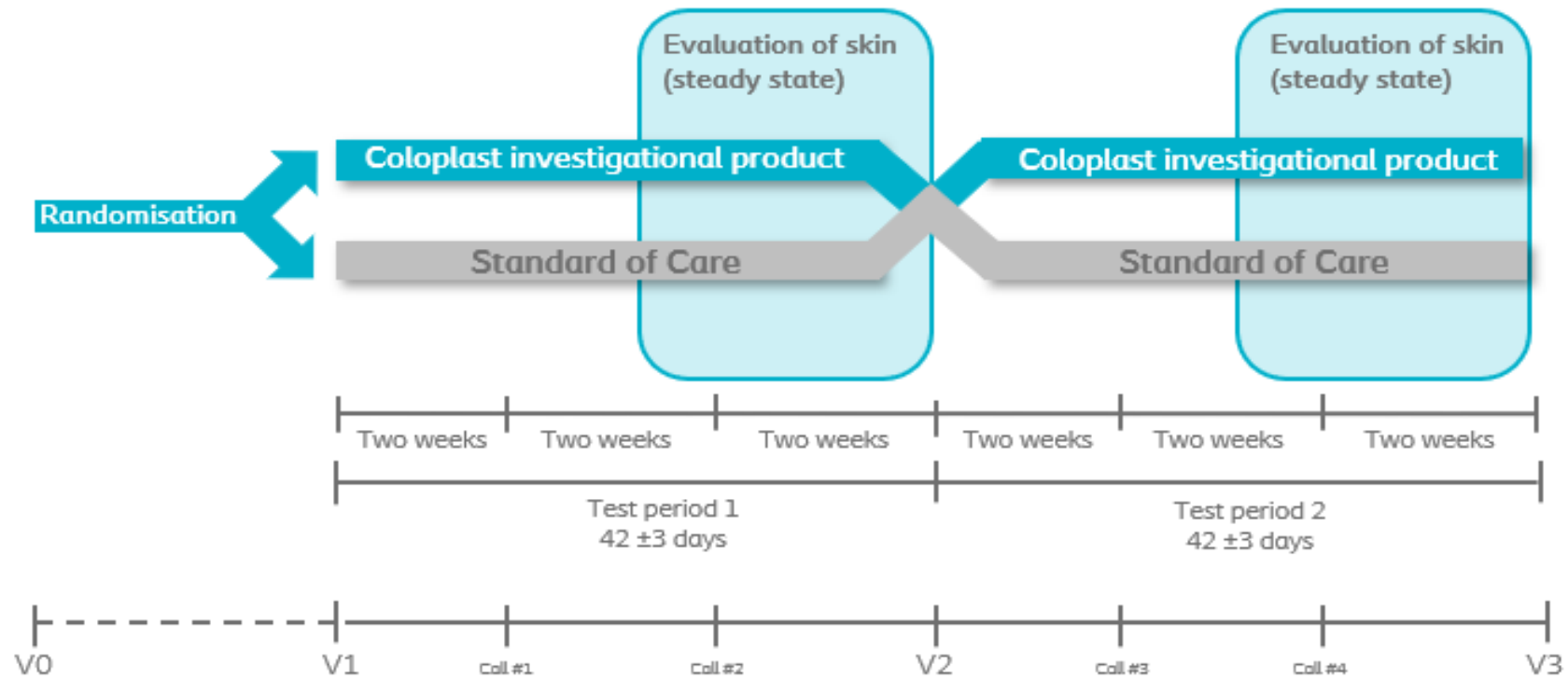

Supplement: Supplemental Information 2 [file peerj-11-16685-s002.pdf]
