# Supplementary material for: Psychometric validation of the Ostomy Skin Tool 2.0
Source: PeerJ. 2023 Dec 18;11:e16685. doi: 10.7717/peerj.16685 (PMC10734405; doi:10.7717/peerj.16685)
Supplement: Supplemental Information 8 [file peerj-11-16685-s008.pdf]

Subject Case Report Forms

Production Germany 14. Jan. 2020 - Primary matrix

Signature Prompt: As investigator I certify that the information submitted within the application is true, complete, and accurate to the best of my knowledge.

Production Germany 14. Jan. 2020: Primary matrix
Form: Subject
Generated On: 30 Sep 2020 12:31:31

Investigator Name	__________________	1
Site Number	__________________	2
Subject Number	__________________	3

Production Germany 14. Jan. 2020: Primary matrix
Form: Subject
Generated On: 30 Sep 2020 12:31:31

	Field Name	Data Type	Units	Values	Pre-Filled Values	Include Field OID
1	INVNAM	$30				INVNAM
2	SITE	$5				SITE
3	SUBJECT	4				SUBJECT

Production Germany 14. Jan. 2020: Primary matrix
Folder: Visit 0
Form: Subject Informed Consent
Generated On: 30 Sep 2020 12:31:31

Please enter date of signed Informed Consent ________________________ (1)

Production Germany 14. Jan. 2020: Primary matrix
Folder: Visit 0
Form: Subject Informed Consent
Generated On: 30 Sep 2020 12:31:31

	Field Name	Data Type	Units	Values	Pre-Filled Values	Include Field OID
1	ICDAT	dd MMM yyyy				ICDAT

Production Germany 14. Jan. 2020: Primary matrix
Folder: Visit 0
Form: Visit Date
Generated On: 30 Sep 2020 12:31:31

Please enter visit date ______________________ (1)

Production Germany 14. Jan. 2020: Primary matrix
Folder: Visit 0
Form: Visit Date
Generated On: 30 Sep 2020 12:31:31

	Field Name	Data Type	Units	Values	Pre-Filled Values	Include Field OID
1	VISITDAT	dd MMM yyyy				VISITDAT

Production Germany 14. Jan. 2020: Primary matrix
Folder: Visit 0
Form: Inclusion Criteria
Generated On: 30 Sep 2020 12:31:31

All fields on the Inclusion Criteria Form are required. The Subject must be able to answer 'Yes' to the following criteria:

Has given written consent to participate by signing the Informed Consent Signature Form

No ▢ (2)
Yes ▢

Has an ileostomy or colostomy with liquid output*
*Definition of liquid output: Six-Seven in the Bristol scale (Appendix 5 - Bristol scale)

No ▢ (3)
Yes ▢

Currently using a flat product

No ▢ (4)
Yes ▢

Be at least 18 years of age and have full legal capacity

No ▢ (5)
Yes ▢

Have had their stoma for at least three months (90 days)

No ▢ (6)
Yes ▢

Can use a product with a max cut size of 40 mm

No ▢ (7)
Yes ▢

Has experienced leakage under the baseplate at least three times within the last fourteen days.
Leakage defined as output seeping under the baseplate

No ▢ (8)
Yes ▢

Has symptoms of peristomal skin complications or has peristomal skin complications defined by at least one of the below.
a)=Has experienced symptoms of skin compli-cations (itching, burning, pain) within the last fourteen days.
b)=Has experienced red skin in the inner circle (within three cm from stoma edge) within the last fourteen days.
c)=Has skin complication (assessed by Principal Investigator, or delegate) in the inner circle (within three cm from stoma edge) of the peristomal area.

No ☐ (9)
Yes ☐

Is able to handle the electronic diary (questionnaire/photo) themselves

No ☐ (10)
Yes ☐

Is able to handle (apply, remove, cut etc.) the product themselves

No ☐ (11)
Yes ☐

Is willing to not use barrier film or barrier cream during the investigation

No ☐ (12)
Yes ☐

Is willing and suitable (determined by Principal Inves-tigator, or delegate) to use a flat custom cut one-piece open or a two-piece open product during the investigation

No ☐ (13)
Yes ☐

Is willing to change the product (1pc) or baseplate (2pc) at least every fourth days

No ☐ (14)
Yes ☐

Production Germany 14. Jan. 2020: Primary matrix
Folder: Visit 0
Form: Inclusion Criteria
Generated On: 30 Sep 2020 12:31:31

	Field Name	Data Type	Units	Values	Pre-Filled Values	Include Field OID
2	Inclusion no. 1	1		0 = No<br>1 = Yes		ITEST1
3	Inclusion no. 2	1		0 = No<br>1 = Yes		ITEST2
4	Inclusion no. 3	1		0 = No<br>1 = Yes		ITEST3
5	Inclusion no. 4	1		0 = No<br>1 = Yes		ITEST4
6	Inclusion no. 5	1		0 = No<br>1 = Yes		ITEST5
7	Inclusion no. 6	1		0 = No<br>1 = Yes		ITEST6
8	Inclusion no. 7	1		0 = No<br>1 = Yes		ITEST7
9	Inclusion no. 8	1		0 = No<br>1 = Yes		ITEST8
10	Inclusion no. 9	1		0 = No<br>1 = Yes		ITEST9
11	Inclusion no. 10	1		0 = No<br>1 = Yes		ITEST10

Production Germany 14. Jan. 2020: Primary matrix
Folder: Visit 0
Form: Inclusion Criteria
Generated On: 30 Sep 2020 12:31:31

	Field Name	Data Type	Units	Values	Pre-Filled Values	Include Field OID
12	Inclusion no. 11	1		0 = No<br>1 = Yes		ITEST11
13	Inclusion no. 12	1		0 = No<br>1 = Yes		ITEST12
14	Inclusion no. 13	1		0 = No<br>1 = Yes		ITEST13

Production Germany 14. Jan. 2020: Primary matrix
Folder: Visit 0
Form: Exclusion criteria
Generated On: 30 Sep 2020 12:31:31

All fields on the Exclusion Criteria Form are required. The Subject must be able to answer 'No' to the following criteria:

Is currently receiving or have within the past 60 days received radio-and/or chemotherapy
-=low doses chemotherapy (assessed by Principal Investigator) is allowed for indi-cations other than cancer

No ☐ 2
Yes ☐

Is currently receiving or have within the past month received topical steroid treatment in the peristomal skin area, e.g. lotion or spray.
-=Low dose systemic steroid treatment (e.g. inhalation) assessed by the Princi-pal Investigator are allowed.
-=Other systemic steroid treatment (e.g. in-jection, or tablet) are not allowed

No ☐ 3
Yes ☐

Is breastfeeding

No ☐ 4
Yes ☐
Not applicable ☐

Is pregnant (based on pregnancy test - urine)

No ☐ 5
Yes ☐
Not applicable ☐

Has known hypersensitivity towards any of the products used in the investigation

No ☐ 6
Yes ☐

Production Germany 14. Jan. 2020: Primary matrix
Folder: Visit 0
Form: Exclusion criteria
Generated On: 30 Sep 2020 12:31:31

	Field Name	Data Type	Units	Values	Pre-Filled Values	Include Field OID
2	Exclusion no. 1	1		0 = No<br>1 = Yes		ETEST1
3	Exclusion no. 2	1		0 = No<br>1 = Yes		ETEST2
4	Exclusion no. 3	1		0 = No<br>1 = Yes<br>2 = Not applicable		ETEST3
5	Exclusion no. 4	1		0 = No<br>1 = Yes<br>2 = Not applicable		ETEST4
6	Exclusion no. 5	1		0 = No<br>1 = Yes		ETEST5

Production Germany 14. Jan. 2020: Primary matrix
Folder: Randomisation
Form: Randomisation Details
Generated On: 30 Sep 2020 12:31:31

Is the subject eligible for inclusion in the study?	No ▢<br>Yes ▢	1
Which product type does the subject use?	One piece product ▢<br>Two piece product ▢	2
Is the subject ready to be randomized?	________	3
Regime Name	________	4
Regime Ratio	________	5
Regime Description	________	6
Date and time of randomization<br>(time in UTC)	________	7
Stratum Name	________	8
Blinded	________	9
Reason for Unblinding	________	10
Error Marking Group	1	11

Production Germany 14. Jan. 2020: Primary matrix
Folder: Randomisation
Form: Randomisation Details
Generated On: 30 Sep 2020 12:31:31

	Field Name	Data Type	Units	Values	Pre-Filled Values	Include Field OID
1	Eligibility	1		0 = No<br>1 = Yes		ELIGIBLE_YN
2	PRODUCT_TYPE	1		1 = One piece product<br>2 = Two piece product		PRODUCT_TYPE
3	RANDTRIG	1				RANDTRIG
4	REGIME_NAME	$200				REGIME_NAME
5	REGIME_RATIO	2				REGIME_RATIO
6	REGIME_DESCRIPTION	$1999				REGIME_DESCRIPTION
7	RANDOMIZED_AT	dd MMM yyyy HH:nn:ss				RANDOMIZED_AT
8	STRATUM_NAME	$50				STRATUM_NAME
9	BLINDED	$6				BLINDED

Production Germany 14. Jan. 2020: Primary matrix
Folder: Randomisation
Form: Randomisation Details
Generated On: 30 Sep 2020 12:31:31

	Field Name	Data Type	Units	Values	Pre-Filled Values	Include Field OID
10	UNBLIND_REASON	$1999				UNBLIND_REASON
11	SYS_ERROR_MARKING_GROUP	1		1		SYS_ERROR_MARKING_GROUP

Production Germany 14. Jan. 2020: Primary matrix
Folder: Visit 1
Form: Visit Date
Generated On: 30 Sep 2020 12:31:31

Please enter visit date ______________________ (1)

Production Germany 14. Jan. 2020: Primary matrix
Folder: Visit 1
Form: Visit Date
Generated On: 30 Sep 2020 12:31:31

	Field Name	Data Type	Units	Values	Pre-Filled Values	Include Field OID
1	VISITDAT	dd MMM yyyy				VISITDAT

Demographics

Date of birth	________	2
Derived age	________	3
Gender	Female ▢<br>Male ▢	4

Production Germany 14. Jan. 2020: Primary matrix
Folder: Visit 1
Form: Demographics
Generated On: 30 Sep 2020 12:31:31

	Field Name	Data Type	Units	Values	Pre-Filled Values	Include Field OID
2	Date of birth	dd MMM yyyy				BRTHDAT
3	Derived age	2				AGE
4	Gender	1		1 = Female<br>2 = Male		GENDER

Production Germany 14. Jan. 2020: Primary matrix
Folder: Visit 1
Form: Vital signs
Generated On: 30 Sep 2020 12:31:31

Height and weight

Height (xxx)	**cm** ▢ (2)<br>in ▢
Weight (xxx)	**kg** ▢ (3)<br>lb ▢

	Field Name	Data Type	Units	Values	Pre-Filled Values	Include Field OID
2	Height	3	cm = cm<br>IN = in			VSHT
3	Weight	3	kg = kg<br>LB = lb			VSWT

Production Germany 14. Jan. 2020: Primary matrix
Folder: Visit 1
Form: Stoma
Generated On: 30 Sep 2020 12:31:31

Information about the stoma

When was the stoma created / month; (if applicable; e.g Jan) ____________ 2

When was the stoma created / Year ____________ 3

Type of stoma — Ileostomy ☐ / Colostomy ☐ 4

What was the reason for creation of the stoma? (you may tick more than 1 box)

Morbus Crohn ____________ 6

Colitis Ulcerosa ____________ 7

Cancer ____________ 8

Other ____________ 9

What is the diameter of the stoma on the widest place? Fixed Unit: mm ____________ 10

What is the height of the stoma? Fixed Unit: mm ____________ 11

What is the shape of the stoma / intestine? Round ☐ / Oval ☐ 12

Irregular ▢

Production Germany 14. Jan. 2020: Primary matrix
Folder: Visit 1
Form: Stoma
Generated On: 30 Sep 2020 12:31:31

	Field Name	Data Type	Units	Values	Pre-Filled Values	Include Field OID
2	Stoma operation month	$3				STOMA_OP MONTH
3	Stoma operation - Year	4				STOMA_OP YEAR
4	Stoma type	1		1 = Ileostomy 2 = Colostomy		STOMA_TY PE
6	Stoma reason 1	1				MULTI_STO MAREASON _1
7	Stoma reason 2	1				MULTI_STO MAREASON _2
8	Stoma reason 3	1				MULTI_STO MAREASON _3
9	Stoma reason 4	1				MULTI_STO MAREASON _4
10	Stoma diameter	2				STOMA_DIA METER

Production Germany 14. Jan. 2020: Primary matrix
Folder: Visit 1
Form: Stoma
Generated On: 30 Sep 2020 12:31:31

	Field Name	Data Type	Units	Values	Pre-Filled Values	Include Field OID
11	Stoma height	2				STOMA_HEIGHT
12	Stoma shape	1		1 = Round<br>2 = Oval<br>3 = Irregular		STOMA_SHAPE

Production Germany 14. Jan. 2020: Primary matrix
Folder: Visit 1
Form: Output
Generated On: 30 Sep 2020 12:31:31

Leakage / output

What is the frequency of the output?	All the time (continuous) ☐ (2)<br>With regular intervals ☐<br>With irregular intervals ☐
What is the consistency of the output evaluated on the Bristol scale?	Type 1 ☐ (3)<br>Type 2 ☐<br>Type 3 ☐<br>Type 4 ☐<br>Type 5 ☐<br>Type 6 ☐<br>Type 7 ☐
Does the subject experience the bag becoming overfilled with air during use (ballooning)?	No ☐ (4)<br>Yes ☐

	Field Name	Data Type	Units	Values	Pre-Filled Values	Include Field OID
2	Output frequency	1		1 = All the time (continuous)<br>2 = With regular intervals<br>3 = With irregular intervals		OUTPUT_FRQ
3	Bristol scale	1		1 = Type 1<br>2 = Type 2<br>3 = Type 3<br>4 = Type 4<br>5 = Type 5<br>6 = Type 6<br>7 = Type 7		BRISTOL_SCALE
4	Ballooning	1		0 = No<br>1 = Yes		BALLOONING

Information about the stoma product normally used

What brand of stoma product is normally used? (you may tick more than 1 box)

Coloplast	______	3
ConvaTec	______	4
Hollister	______	5
Dansac	______	6
Salts	______	7
Other	______	8
If 'Other', please specify	______	9
What is the product name?	______	10
What is the product number / item number?	______	11
What is the product size (base plate size)?	______	12
Is the subject used to cutting the product / baseplate?	No ☐<br>Yes ☐	13
If Yes, how much is cut off - minimum value?	Fixed Unit: mm<br>______	14

If Yes, how much is cut off - maximum value? Fixed Unit: mm 15

On average, how often is the product/baseplate normally changed? 16

- Once a day or more frequent ☐
- Every 2nd day ☐
- Every 3rd day ☐
- Every 4th day ☐
- Every 5th day ☐
- Every 6th day ☐
- Once a week or less frequent ☐

Production Germany 14. Jan. 2020: Primary matrix
Folder: Visit 1
Form: Normal device
Generated On: 30 Sep 2020 12:31:31

	Field Name	Data Type	Units	Values	Pre-Filled Values	Include Field OID
3	Brand 1	1				MULTI_BRAND_1
4	Brand 2	1				MULTI_BRAND_2
5	Brand 3	1				MULTI_BRAND_3
6	Brand 4	1				MULTI_BRAND_4
7	Brand 5	1				MULTI_BRAND_5
8	Brand 6	1				MULTI_BRAND_6
9	Brand other	$50				MULTI_BRAND_OTHER
10	Product name	$100				PRODUCT_NAME
11	Product number	$50				PRODUCT_NUMBER
12	Product size	$30				PRODUCT_SIZE

Production Germany 14. Jan. 2020: Primary matrix
Folder: Visit 1
Form: Normal device
Generated On: 30 Sep 2020 12:31:31

	Field Name	Data Type	Units	Values	Pre-Filled Values	Include Field OID
13	Cutting YN	1		0 = No<br>1 = Yes		CUTTING_YN
14	Cutting min	2				CUTTING_MIN
15	Cutting max	2				CUTTING_MAX
16	Frequency of change	1		1 = Once a day or more frequent<br>2 = Every 2nd day<br>3 = Every 3rd day<br>4 = Every 4th day<br>5 = Every 5th day<br>6 = Every 6th day<br>7 = Once a week or less frequent		CHANGE_FREQ

Use of accessories

Which stoma accessories are normally used? (you may tick more than one box)

None	3
Adhesive remover	4
Paste	5
Rings	6
Ostomy tape	7
Ostomy belt	8
Hernia belt	9
Stoma powder	10
Barrier lotion	11
Barrier cream	12
Barrier spray	13
Barrier wipes	14

Production Germany 14. Jan. 2020: Primary matrix
Folder: Visit 1
Form: Accessories
Generated On: 30 Sep 2020 12:31:31

Cleansing wipes/cleansing spray	____________	15
Odour remover	____________	16
Other accessories	____________	17
If 'Other' please specify	____________	18

Production Germany 14. Jan. 2020: Primary matrix
Folder: Visit 1
Form: Accessories
Generated On: 30 Sep 2020 12:31:31

	Field Name	Data Type	Units	Values	Pre-Filled Values	Include	Field OID
3	Accessories 1 1						MULTI_ACC_1
4	Accessories 1 2						MULTI_ACC_2
5	Accessories 1 3						MULTI_ACC_3
6	Accessories 1 4						MULTI_ACC_4
7	Accessories 1 5						MULTI_ACC_5
8	Accessories 1 6						MULTI_ACC_6
9	Accessories 1 7						MULTI_ACC_7
10	Accessories 1 8						MULTI_ACC_8
11	Accessories 1 9						MULTI_ACC_9
12	Accessories 1 10						MULTI_ACC_10

	Field Name	Data Type	Units	Values	Pre-Filled Values	Include Field OID
13	Accessories 11	1				MULTI_ACC_11
14	Accessories 12	1				MULTI_ACC_12
15	Accessories 13	1				MULTI_ACC_13
16	Accessories 14	1				MULTI_ACC_14
17	Accessories 15	1				MULTI_ACC_15
18	Accessories other	$50				MULTI_ACC_OTHER

Production Germany 14. Jan. 2020: Primary matrix
Folder: Visit 1
Form: Body Profile
Generated On: 30 Sep 2020 12:31:31

Body Profile

Please refer to the Body Profile Guide in the Site File and describe the area that is usually covered by the base plate with regard to the following parameters

---

The form around the stoma (picture in box 1)	Regular ☐<br>Inward ☐<br>Outward ☐	3
Selected form being uniform or variable (picture in box 2)	Uniform ☐<br>Variable ☐	4
Soft or firm abdomen (pictures in box 3)	Soft ☐<br>Firm ☐	5
Superficial creases or deep folds (pictures in box 4)	No creases or superficial creases ☐<br>Deep folds ☐	6
Location of stoma (pictures in box 5)	Above bending line ☐<br>At bending line ☐<br>Below bending line ☐	7
Level of stoma opening in relation to skin surface (pictures in box 6)	Above skin surface ☐<br>In level with skin surface ☐<br>Below skin surface ☐	8
Consistency of output	Thick stool ☐	9

Liquid stool ☐

Production Germany 14. Jan. 2020: Primary matrix
Folder: Visit 1
Form: Body Profile
Generated On: 30 Sep 2020 12:31:31

	Field Name	Data Type	Units	Values	Pre-Filled Values	Include Field OID
3	Body Profile 1	1		1 = Regular<br>2 = Inward<br>3 = Outward		PROFILE_1
4	Body Profile 2	1		1 = Uniform<br>2 = Variable		PROFILE_2
5	Body Profile 3	1		1 = Soft<br>2 = Firm		PROFILE_3
6	Body Profile 4	1		1 = No creases or superficial creases<br>2 = Deep folds		PROFILE_4
7	Body Profile 5	1		1 = Above bending line<br>2 = At bending line<br>3 = Below bending line		PROFILE_5
8	Body Profile 6	1		1 = Above skin surface<br>2 = In level with skin surface		PROFILE_6

Field Name	Data Type	Units	Values	Pre-Filled Values	Include Field OID
			3 = Below skin surface		
Body Profile 7	1		1 = Thick stool<br>2 = Liquid stool		PROFILE_7

Peristomal skin area

Please ask the subject the following questions:

How often do you experience skin complications around the stoma (itching, burning, red or discoloured skin, pain, bleeding, moist or broken skin)? (2)

- Daily ☐
- Every 2-3 days ☐
- Every 4-6 days ☐
- Once a week ☐
- Every second week ☐
- More rarely ☐

How many times in the last year have you had a peristomal skin complication where you had to seek consultation and/or treatment from a health care professional? (3) ______________________

Which peristomal skin complication/symptom do you experience most often? (you may tick more than one box)

Itching ______________________ (5)

Burning ______________________ (6)

Red/discoloured skin ______________________ (7)

Pain ______________________ (8)

Bleeding skin ______________________ (9)

Moist skin ______________________ (10)

Broken skin ____________________ 11

Do you have any skin complications around your stoma today? 12

- No ☐
- Yes ☐

How would you describe the skin complications around your stoma today? 13

- No skin complications ☐
- Very mild ☐
- Mild ☐
- Moderate ☐
- Severe ☐
- Very severe ☐

Peristomal skin area

Please evaluate the peristomal skin - for site personnel to evaluate

Does the subject have any peristomal skin complications today? 15

- No ☐
- Yes ☐

Overall, how would you describe the severity of the subjects peristomal skin complications today? 16

- Very mild ☐
- Mild ☐
- Moderate ☐
- Severe ☐
- Very severe ☐

Does the subject have any discoloration on the peristomal skin today? 17

- No ☐
- Yes ☐

Overall, how would you describe the severity of the subject’s discoloration on their peristomal skin today?

Very mild ▢ 18
Mild ▢
Moderate ▢
Severe ▢
Very severe ▢

Does the subject have any erosion on the peristomal skin today?

No ▢ 19
Yes ▢

Overall, how would you describe the severity of the subject’s erosion on their peristomal skin complications today?

Very mild ▢ 20
Mild ▢
Moderate ▢
Severe ▢
Very severe ▢

Is there discoloration of peristomal skin with complications (pain, shiny, indurated, hot, itching, burning)?

No ▢ 21
Yes ▢

Is there damage to the lower layers of the skin with complications (moisture, bleeding or ulceration)?

No ▢ 22
Yes ▢

Is there raised tissue above skin level with complications (bleeding, pain or moisture)?

No ▢ 23
Yes ▢

Please remember to check for adverse events, device deficiencies and concomitant medication

---

	Field Name	Data Type	Units	Values	Pre-Filled Values	Include	Field OID
2	Frequency of complications	1		1 = Daily<br>2 = Every 2-3 days<br>3 = Every 4-6 days<br>4 = Once a week<br>5 = Every second week<br>6 = More rarely			COMP_FRQ
3	Number of consultations	$5					COMP_CONSULT
5	Skin complication 1	1					MULTI_COMPTYPE_1
6	Skin complication 2	1					MULTI_COMPTYPE_2
7	Skin complication 3	1					MULTI_COMPTYPE_3
8	Skin complication 4	1					MULTI_COMPTYPE_4

Production Germany 14. Jan. 2020: Primary matrix
Folder: Visit 1
Form: Skin area visit 1
Generated On: 30 Sep 2020 12:31:31

	Field Name	Data Type	Units	Values	Pre-Filled Values	Include Field OID
9	Skin complication 5	1				MULTI_COMPTYPE_5
10	Skin complication 6	1				MULTI_COMPTYPE_6
11	Skin complication 7	1				MULTI_COMPTYPE_7
12	Complications today	1		0 = No<br>1 = Yes		COMP_TODAY
13	Severity of complications	1		0 = No skin complications<br>1 = Very mild<br>2 = Mild<br>3 = Moderate<br>4 = Severe<br>5 = Very severe		COMP_IF_YES
15	Skin complications	1		0 = No<br>1 = Yes		COMPLICATION_YN

	Field Name	Data Type	Units	Values	Pre-Filled Values	Include Field OID
16	Skin complications severity	1		1 = Very mild<br>2 = Mild<br>3 = Moderate<br>4 = Severe<br>5 = Very severe		COMPLICATION_IF_YES
17	Discolouration	1		0 = No<br>1 = Yes		DISCOLOUR_YN
18	Discolouration severity	1		1 = Very mild<br>2 = Mild<br>3 = Moderate<br>4 = Severe<br>5 = Very severe		DISCOLOUR_IF_YES
19	Erosion	1		0 = No<br>1 = Yes		EROSION_YN
20	Erosion severity	1		1 = Very mild<br>2 = Mild<br>3 = Moderate<br>4 = Severe<br>5 = Very severe		EROSION_IF_YES

Production Germany 14. Jan. 2020: Primary matrix
Folder: Visit 1
Form: Skin area visit 1
Generated On: 30 Sep 2020 12:31:31

	Field Name	Data Type	Units	Values	Pre-Filled Values	Include Field OID
21	Discolouration complications	1		0 = No<br>1 = Yes		DISCOLOUR_COMP_YN
22	Erosion complications	1		0 = No<br>1 = Yes		EROSION_COMP_YN
23	Tissue complications	1		0 = No<br>1 = Yes		TISSUE_COMP_YN

Administration of study supplies
Please register the supplies handed out to this subject at this visit

Please enter the name of the product handed out at this visit	______________________	2
Please enter the number of boxes handed out	______________________	3
Please enter the subject number from the phone that is handed out. PLEASE NOTE: it is extremely important that the subject number in Rave and on the phone is identical. Please also take the first photo at the visit.	______________________	4

Production Germany 14. Jan. 2020: Primary matrix
Folder: Visit 1
Form: Study supplies visit 1
Generated On: 30 Sep 2020 12:31:31

	Field Name	Data Type	Units	Values	Pre-Filled Values	Include Field OID
2	Product supplied	$100				SUPPLIES_1
3	Boxes supplied	2				SUPPLIES_2
4	Phone supplied	4				SUPPLIES_3

Registration of phone call

Date of the call ______ (2)

Please check the subject's well-being and make sure to ask the subject the following questions

Have you seen a change in the skin condition since the last visit/call? No ☐ Yes ☐ (4)

If yes, please describe ______ (5)

Did the stoma behave differently from normal since the last visit/call? No ☐ Yes ☐ (6)

If the stoma behaved differently, please describe ______ (7)

Please remember to check for adverse events, device deficiencies and concomitant medication

Production Germany 14. Jan. 2020: Primary matrix
Folder: Phone call week 2
Form: Phone call
Generated On: 30 Sep 2020 12:31:31

	Field Name	Data Type	Units	Values	Pre-Filled Values	Include Field OID
2	Date of call	dd MMM yyyy				VISITDAT
4	Check of skin	1		0 = No<br>1 = Yes		SKINCHECK
5	If yes, please describe	$300				SKINCHECK _IF_YES
6	Check of stoma	1		0 = No<br>1 = Yes		STOMA_CH ECK
7	If yes please describe	$300				STOMACHE CK_IF_YES

Production Germany 14. Jan. 2020: Primary matrix
Folder: Phone call week 4
Form: Phone call
Generated On: 30 Sep 2020 12:31:31

Registration of phone call

Date of the call ______________ 2

Please check the subject's well-being and make sure to ask the subject the following questions

---

Have you seen a change in the skin condition since the last visit/call? No ☐ Yes ☐ 4

If yes, please describe ______________ 5

Did the stoma behave differently from normal since the last visit/call? No ☐ Yes ☐ 6

If the stoma behaved differently, please describe ______________ 7

Please remember to check for adverse events, device deficiencies and concomitant medication

---

Production Germany 14. Jan. 2020: Primary matrix
Folder: Phone call week 4
Form: Phone call
Generated On: 30 Sep 2020 12:31:31

	Field Name	Data Type	Units	Values	Pre-Filled Values	Include Field OID
2	Date of call	dd MMM yyyy				VISITDAT
4	Check of skin	1		0 = No<br>1 = Yes		SKINCHECK
5	If yes, please describe	$300				SKINCHECK _IF_YES
6	Check of stoma	1		0 = No<br>1 = Yes		STOMA_CH ECK
7	If yes please describe	$300				STOMACHE CK_IF_YES

Production Germany 14. Jan. 2020: Primary matrix
Folder: Visit 2
Form: Visit Date
Generated On: 30 Sep 2020 12:31:31

Please enter visit date ______________________ (1)

Production Germany 14. Jan. 2020: Primary matrix
Folder: Visit 2
Form: Visit Date
Generated On: 30 Sep 2020 12:31:31

	Field Name	Data Type	Units	Values	Pre-Filled Values	Include Field OID
1	VISITDAT	dd MMM yyyy				VISITDAT

Peristomal skin area
Please evaluate the peristomal skin

Does the subject have any peristomal skin complications today?	No ☐<br>Yes ☐	2
Overall, how would you describe the severity of the subject's peristomal skin complications today?	Very mild ☐<br>Mild ☐<br>Moderate ☐<br>Severe ☐<br>Very severe ☐	3
Does the subject have any discoloration on the peristomal skin today?	No ☐<br>Yes ☐	4
Overall, how would you describe the severity of the subject’s discoloration on their peristomal skin today?	Very mild ☐<br>Mild ☐<br>Moderate ☐<br>Severe ☐<br>Very severe ☐	5
Does the subject have any erosion on the peristomal skin today?	No ☐<br>Yes ☐	6
Overall, how would you describe the severity of the subject’s erosion on their peristomal skin complications today?	Very mild ☐<br>Mild ☐<br>Moderate ☐<br>Severe ☐<br>Very severe ☐	7

Is there discoloration of peristomal skin with complications (pain, shiny, indurated, hot, itching, burning)?

No ☐ (8)
Yes ☐

Is there damage to the lower layers of the skin with complications (moisture, bleeding or ulceration)?

No ☐ (9)
Yes ☐

Is there raised tissue above skin level with complications (bleeding, pain or moisture)?

No ☐ (10)
Yes ☐

Compared to the beginning of this test period (Test Period 1) how have the subject's peristomal skin complications changed during the study? (based on photo from visit 1 and the skin at visit 2)

Very much improved ☐ (11)
Much improved ☐
A little improved ☐
No change ☐
A little worse ☐
Much worse ☐
Very much worse ☐

Compared to the beginning of this test period (test period 1) how has the discolouration of the subject's skin around the stoma changed during the study? (based on photo from visit 1 and the skin at visit 2)

Very much improved ☐ (12)
Much improved ☐
A little improved ☐
No change ☐
A little worse ☐
Much worse ☐
Very much worse ☐

Compared to the beginning of this test period (test period 1) how has the erosion of the subject's skin around the stoma changed during the study? (based on photo from visit 1 and the skin at visit 2)

Very much improved ☐ (13)
Much improved ☐
A little improved ☐

No change ☐
A little worse ☐
Much worse ☐
Very much worse ☐

Please remember to check for adverse events, device deficiencies and concomitant medication

---

Production Germany 14. Jan. 2020: Primary matrix
Folder: Visit 2
Form: Skin area visit 2
Generated On: 30 Sep 2020 12:31:31

	Field Name	Data Type	Units	Values	Pre-Filled Values	Include Field OID
2	Skin complications	1		0 = No<br>1 = Yes		COMPLICATION_YN
3	Skin complications severity	1		1 = Very mild<br>2 = Mild<br>3 = Moderate<br>4 = Severe<br>5 = Very severe		COMPLICATION_IF_YES
4	Discolouration	1		0 = No<br>1 = Yes		DISCOLOUR_YN
5	Discolouration severity	1		1 = Very mild<br>2 = Mild<br>3 = Moderate<br>4 = Severe<br>5 = Very severe		DISCOLOUR_IF_YES
6	Erosion	1		0 = No<br>1 = Yes		EROSION_YN
7	Erosion severity	1		1 = Very mild<br>2 = Mild<br>3 = Moderate		EROSION_IF_YES

	Field Name	Data Type	Units	Values	Pre-Filled Values	Include Field OID
				4 = Severe<br>5 = Very severe		
8	Discolouration complications	1		0 = No<br>1 = Yes		DISCOLOUR_COMP_YN
9	Erosion complications	1		0 = No<br>1 = Yes		EROSION_COMP_YN
10	Tissue complications	1		0 = No<br>1 = Yes		TISSUE_COMP_YN
11	Comparison of skin	1		1 = Very much improved<br>2 = Much improved<br>3 = A little improved<br>4 = No change<br>5 = A little worse<br>6 = Much worse<br>7 = Very much worse		COMPARE_SKIN

	Field Name	Data Type	Units	Values	Pre-Filled Values	Include Field OID
12	Comparison of discolouration	1		1 = Very much improved<br>2 = Much improved<br>3 = A little improved<br>4 = No change<br>5 = A little worse<br>6 = Much worse<br>7 = Very much worse		COMPARE_ DISCOLOUR
13	Comparison of erosion	1		1 = Very much improved<br>2 = Much improved<br>3 = A little improved<br>4 = No change<br>5 = A little worse<br>6 = Much worse<br>7 = Very much worse		COMPARE_ EROSION

Production Germany 14. Jan. 2020: Primary matrix
Folder: Visit 2
Form: Study supplies visit 2
Generated On: 30 Sep 2020 12:31:31

Administration of study supplies
Please register the supplies handed out to this subject at this visit

---

______________________ (2)

______________________ (3)

Please confirm that the phone has been configured, so the app is pushed forward to test period 2. Please also take the first photo at the visit.	No ▢ Yes ▢	(4)

Production Germany 14. Jan. 2020: Primary matrix
Folder: Visit 2
Form: Study supplies visit 2
Generated On: 30 Sep 2020 12:31:31

	Field Name	Data Type	Units	Values	Pre-Filled Values	Include Field OID
2	Product supplied	$100				SUPPLIES_ 1
3	Boxes supplied	2				SUPPLIES_ 2
4	Phone configured	1		0 = No<br>1 = Yes		SUPPLIES_ 4

Production Germany 14. Jan. 2020: Primary matrix
Folder: Phone call week 8
Form: Phone call
Generated On: 30 Sep 2020 12:31:31

Registration of phone call

Date of the call ______ (2)

Please check the subject's well-being and make sure to ask the subject the following questions

---

Have you seen a change in the skin condition since the last visit/call? No ☐ Yes ☐ (4)

If yes, please describe ______ (5)

Did the stoma behave differently from normal since the last visit/call? No ☐ Yes ☐ (6)

If the stoma behaved differently, please describe ______ (7)

Please remember to check for adverse events, device deficiencies and concomitant medication

---

	Field Name	Data Type	Units	Values	Pre-Filled Values	Include Field OID
2	Date of call	dd MMM yyyy				VISITDAT
4	Check of skin	1		0 = No<br>1 = Yes		SKINCHECK
5	If yes, please describe	$300				SKINCHECK_IF_YES
6	Check of stoma	1		0 = No<br>1 = Yes		STOMA_CHECK
7	If yes please describe	$300				STOMACHECK_IF_YES

Production Germany 14. Jan. 2020: Primary matrix
Folder: Phone call week 10
Form: Phone call
Generated On: 30 Sep 2020 12:31:31

Registration of phone call

Date of the call ________________ (2)

Please check the subject's well-being and make sure to ask the subject the following questions

---

Have you seen a change in the skin condition since the last visit/call? No ☐ Yes ☐ (4)

If yes, please describe ________________ (5)

Did the stoma behave differently from normal since the last visit/call? No ☐ Yes ☐ (6)

If the stoma behaved differently, please describe ________________ (7)

Please remember to check for adverse events, device deficiencies and concomitant medication

---

	Field Name	Data Type	Units	Values	Pre-Filled Values	Include Field OID
2	Date of call	dd MMM yyyy				VISITDAT
4	Check of skin	1		0 = No<br>1 = Yes		SKINCHECK
5	If yes, please describe	$300				SKINCHECK_IF_YES
6	Check of stoma	1		0 = No<br>1 = Yes		STOMA_CHECK
7	If yes please describe	$300				STOMACHECK_IF_YES

Production Germany 14. Jan. 2020: Primary matrix
Folder: Visit 3
Form: Visit Date
Generated On: 30 Sep 2020 12:31:31

Please enter visit date ______________________________ (1)

Production Germany 14. Jan. 2020: Primary matrix
Folder: Visit 3
Form: Visit Date
Generated On: 30 Sep 2020 12:31:31

	Field Name	Data Type	Units	Values	Pre-Filled Values	Include Field OID
1	VISITDAT	dd MMM yyyy				VISITDAT

Peristomal skin area
Please evaluate the peristomal skin

Does the subject have any peristomal skin complications today?	No ☐ 2<br>Yes ☐
Overall, how would you describe the severity of the subjects peristomal skin complications today?	Very mild ☐ 3<br>Mild ☐<br>Moderate ☐<br>Severe ☐<br>Very severe ☐
Does the subject have any discoloration on the peristomal skin today?	No ☐ 4<br>Yes ☐
Overall, how would you describe the severity of the subject’s discoloration on their peristomal skin today?	Very mild ☐ 5<br>Mild ☐<br>Moderate ☐<br>Severe ☐<br>Very severe ☐
Does the subject have any erosion on the peristomal skin today?	No ☐ 6<br>Yes ☐
Overall, how would you describe the severity of the subject’s erosion on their peristomal skin complications today?	Very mild ☐ 7<br>Mild ☐<br>Moderate ☐<br>Severe ☐<br>Very severe ☐

Is there discoloration of peristomal skin with complications (pain, shiny, indurated, hot, itching, burning)?

No ☐ (8)
Yes ☐

Is there damage to the lower layers of the skin with complications (moisture, bleeding or ulceration)?

No ☐ (9)
Yes ☐

Is there raised tissue above skin level with complications (bleeding, pain or moisture)?

No ☐ (10)
Yes ☐

Compared to the beginning of this test period (Test Period 2) how have the subject's peristomal skin complications changed during the study? (based on photo from visit 2 and the skin at visit 3)

Very much improved ☐ (11)
Much improved ☐
A little improved ☐
No change ☐
A little worse ☐
Much worse ☐
Very much worse ☐

Compared to the beginning of this test period (test period 2) how has the discolouration of the subject's skin around the stoma changed during the study? (based on photo from visit 2 and the skin at visit 3)

Very much improved ☐ (12)
Much improved ☐
A little improved ☐
No change ☐
A little worse ☐
Much worse ☐
Very much worse ☐

Compared to the beginning of this test period (test period 2) how has the erosion of the subject's skin around the stoma changed during the study? (based on photo from visit 2 and the skin at visit 3)

Very much improved ☐ (13)
Much improved ☐
A little improved ☐

No change ☐
A little worse ☐
Much worse ☐
Very much worse ☐

Please remember to check for adverse events, device deficiencies and concomitant medication

---

	Field Name	Data Type	Units	Values	Pre-Filled Values	Include Field OID
2	Skin complications	1		0 = No<br>1 = Yes		COMPLICATION_YN
3	Skin complications severity	1		1 = Very mild<br>2 = Mild<br>3 = Moderate<br>4 = Severe<br>5 = Very severe		COMPLICATION_IF_YES
4	Discolouration	1		0 = No<br>1 = Yes		DISCOLOUR_YN
5	Discolouration severity	1		1 = Very mild<br>2 = Mild<br>3 = Moderate<br>4 = Severe<br>5 = Very severe		DISCOLOUR_IF_YES
6	Erosion	1		0 = No<br>1 = Yes		EROSION_YN
7	Erosion severity	1		1 = Very mild<br>2 = Mild<br>3 = Moderate		EROSION_IF_YES

	Field Name	Data Type	Units	Values	Pre-Filled Values	Include Field OID
				4 = Severe<br>5 = Very severe		
8	Discolouration complications	1		0 = No<br>1 = Yes		DISCOLOUR_COMP_YN
9	Erosion complications	1		0 = No<br>1 = Yes		EROSION_COMP_YN
10	Tissue complications	1		0 = No<br>1 = Yes		TISSUE_COMP_YN
11	Comparison of skin	1		1 = Very much improved<br>2 = Much improved<br>3 = A little improved<br>4 = No change<br>5 = A little worse<br>6 = Much worse<br>7 = Very much worse		COMPARE_SKIN

	Field Name	Data Type	Units	Values	Pre-Filled Values	Include Field OID
12	Comparison of discolouration	1		1 = Very much improved<br>2 = Much improved<br>3 = A little improved<br>4 = No change<br>5 = A little worse<br>6 = Much worse<br>7 = Very much worse		COMPARE_ DISCOLOUR
13	Comparison of erosion	1		1 = Very much improved<br>2 = Much improved<br>3 = A little improved<br>4 = No change<br>5 = A little worse<br>6 = Much worse<br>7 = Very much worse		COMPARE_ EROSION

Registration of phone call

Date of the call ______________ (2)

Please check the subject's well-being and make sure to ask the subject the following questions

---

Have you seen a change in the skin condition since the last visit/call? No ☐ Yes ☐ (4)

If yes, please describe ______________ (5)

Did the stoma behave differently from normal since the last visit/call? No ☐ Yes ☐ (6)

If the stoma behaved differently, please describe ______________ (7)

Please remember to check for adverse events, device deficiencies and concomitant medication

---

Production Germany 14. Jan. 2020: Primary matrix
Folder: Unscheduled call
Form: Phone call
Generated On: 30 Sep 2020 12:31:31

	Field Name	Data Type	Units	Values	Pre-Filled Values	Include Field OID
2	Date of call	dd MMM yyyy				VISITDAT
4	Check of skin	1		0 = No<br>1 = Yes		SKINCHECK
5	If yes, please describe	$300				SKINCHECK_IF_YES
6	Check of stoma	1		0 = No<br>1 = Yes		STOMA_CHECK
7	If yes please describe	$300				STOMACHECK_IF_YES

Protocol Deviation Form
This form is used for deviations related to data. In case of several deviations, please fill in one form for each deviation

---

Please state the date the deviation took place ________ (2)

Please state what the deviation is related to: (3)

- Informed consent ☐
- Inclusion / exclusion criteria ☐
- Study procedures ☐
- Safety registrations ☐
- Randomisation ☐
- Other ☐

If 'Other', please describe ________ (4)

Please provide any supplementary description of the deviation if applicable ________ (5)

Please describe any actions taken with regards to the deviation ________ (6)

Production Germany 14. Jan. 2020: Primary matrix
Folder: Deviations
Form: Deviation Form
Generated On: 30 Sep 2020 12:31:31

	Field Name	Data Type	Units	Values	Pre-Filled Values	Include Field OID
2	Date	dd MMM yyyy				DATE
3	Type of deviation	2		1 = Informed consent<br>2 = Inclusion / exclusion criteria<br>3 = Study procedures<br>4 = Safety registrations<br>5 = Randomisation<br>6 = Other		DEVIATION_TYPE
4	Other deviation type	$100				DEV_OTHER
5	Description of deviation	$500				DEV_TERM
6	Action after deviation	$200				DEV_ACTION

Did the subject experience any adverse events since last visit / call?	No ☐ (1)<br>Yes ☐

Production Germany 14. Jan. 2020: Primary matrix
Folder: Adverse event summary
Form: Adverse/Serious Events Summary
Generated On: 30 Sep 2020 12:31:31

	Field Name	Data Type	Units	Values	Pre-Filled Values	Include Field OID
1	AEYN	1		0 = No<br>1 = Yes		AEYN

Did the subject experience any device deficiencies since last visit / call?	No ▢ (1)<br>Yes ▢

Production Germany 14. Jan. 2020: Primary matrix
Folder: Device deficiency summary
Form: Device Deficiency Summary
Generated On: 30 Sep 2020 12:31:31

	Field Name	Data Type	Units	Values	Pre-Filled Values	Include Field OID
1	DDYN	1		0 = No<br>1 = Yes		DDYN

Does the subject use any concomitant medication or did the subject change concomitant medications since last visit / call?	No ☐ (1)<br>Yes ☐

Production Germany 14. Jan. 2020: Primary matrix
Folder: Con medication summary
Form: Concomitant Medication Summary
Generated On: 30 Sep 2020 12:31:31

	Field Name	Data Type	Units	Values	Pre-Filled Values	Include Field OID
1	CMYN	1		0 = No<br>1 = Yes		CMYN

Termination Form

Date of completion/discontinuation of the study: ________ (2)

Did the subject complete the study as planned? (3)
- No ☐
- Yes ☐

Please select the primary reason for the discontinuation: (4)
- Adverse Event ☐
- Device deficiency, which led to an Adverse Event ☐
- Device deficiency, which DID NOT lead to an Adverse Event ☐
- Lack of effectiveness ☐
- Subject wishes to discontinue ☐
- Protocol deviation ☐
- Screening failure ☐
- Withdrawal of Consent ☐
- Lost to Follow-Up ☐
- Other ☐

If Protocol Deviation, please specify: ________ (5)

If Other, please specify: ________ (6)

Production Germany 14. Jan. 2020: Primary matrix
Folder: Termination
Form: Termination Form
Generated On: 30 Sep 2020 12:31:31

	Field Name	Data Type	Units	Values	Pre-Filled Values	Include Field OID
2	DSSTDAT	dd MMM yyyy				DSSTDAT
3	DSDECOD	1		0 = No<br>1 = Yes		DSDECOD
4	DSTERM	$35		1 = Adverse Event<br>2 = Device deficiency, which led to an Adverse Event<br>3 = Device deficiency, which DID NOT lead to an Adverse Event<br>4 = Lack of effectivenes s<br>5 = Subject wishes to discontinue<br>6 = Protocol deviation<br>9 = Screening failure<br>10 = Withdrawal of Consent		DSTERM

	Field Name	Data Type	Units	Values	Pre-Filled Values	Include Field OID
				7 = Lost to Follow-Up<br>8 = Other		
5	DSPD	$100				DSPD
6	DSOTH	$100				DSOTH

Do you have any skin complications around your stoma today? (1)

- No ☐
- Yes ☐

If yes, how would you describe the skin complications around your stoma today? (2)

- No skin complications ☐
- Very mild ☐
- Mild ☐
- Moderate ☐
- Severe ☐
- Very severe ☐

In the last seven days, due to leakage or worry about leakage... (3) ____________

I felt panic (4)

- All of the time ☐
- Often ☐
- Sometimes ☐
- Rarely or never ☐

I felt stressed out (5)

- All of the time ☐
- Often ☐
- Sometimes ☐
- Rarely or never ☐

I felt more afraid about leaks in the future (6)

- All of the time ☐
- Often ☐
- Sometimes ☐
- Rarely or never ☐

I felt worry (7)

- All of the time ☐

	Often ☐<br>Sometimes ☐<br>Rarely or never ☐
I felt frustrated	All of the time ☐ (8)<br>Often ☐<br>Sometimes ☐<br>Rarely or never ☐
I felt embarrassed	All of the time ☐ (9)<br>Often ☐<br>Sometimes ☐<br>Rarely or never ☐
I felt worried that I might leak	All of the time ☐ (10)<br>Often ☐<br>Sometimes ☐<br>Rarely or never ☐
I couldn't sleep	All of the time ☐ (11)<br>Often ☐<br>Sometimes ☐<br>Rarely or never ☐
I kept waking up at night to check my stoma	All of the time ☐ (12)<br>Often ☐<br>Sometimes ☐<br>Rarely or never ☐

I kept checking my ostomy bag to see if I have leaked — 13

- All of the time ☐
- Often ☐
- Sometimes ☐
- Rarely or never ☐

Ususal and Social activities — 14
In the last seven days due to leakage or worry about leakage...

I decided to stay at home — 15

- All of the time ☐
- Often ☐
- Sometimes ☐
- Rarely or never ☐
- Not applicable ☐

I couldn't do light activities — 16

- All of the time ☐
- Often ☐
- Sometimes ☐
- Rarely or never ☐
- Not applicable ☐

I changed my plans — 17

- All of the time ☐
- Often ☐
- Sometimes ☐
- Rarely or never ☐
- Not applicable ☐

I was unable to go out and meet family and friends — 18

- All of the time ☐
- Often ☐

	Sometimes ☐<br>Rarely or never ☐<br>Not applicable ☐
I avoided close physical contact with family and friends	All of the time ☐ 19<br>Often ☐<br>Sometimes ☐<br>Rarely or never ☐<br>Not applicable ☐
I did not want to see people	All of the time ☐ 20<br>Often ☐<br>Sometimes ☐<br>Rarely or never ☐<br>Not applicable ☐
I avoided people	All of the time ☐ 21<br>Often ☐<br>Sometimes ☐<br>Rarely or never ☐<br>Not applicable ☐
I tried to avoid meeting new people	All of the time ☐ 22<br>Often ☐<br>Sometimes ☐<br>Rarely or never ☐<br>Not applicable ☐

Coping and in control
In the last 7 days, due to leakage or worry about leakage... ______ 23

I felt in control — All of the time ☐ 24 / Often ☐ / Sometimes ☐ / Rarely or never ☐

I was able to cope — All of the time ☐ 25 / Often ☐ / Sometimes ☐ / Rarely or never ☐

I felt calm — All of the time ☐ 26 / Often ☐ / Sometimes ☐ / Rarely or never ☐

I saw my friends as I usually do — All of the time ☐ 27 / Often ☐ / Sometimes ☐ / Rarely or never ☐

Discreetness
In the last seven days... ______ 28

It was difficult to hide the stoma appliance under clothing — Strongly agree ☐ 29 / Slightly agree ☐

	Neither agree nor disagree ☐<br>Slightly disagree ☐<br>Strongly disagree ☐
I was self-conscious about the appearance of the stoma appliance	Strongly agree ☐ 30<br>Slightly agree ☐<br>Neither agree nor disagree ☐<br>Slightly disagree ☐<br>Strongly disagree ☐
The stoma appliance limited the choice of clothes that I could wear	Strongly agree ☐ 31<br>Slightly agree ☐<br>Neither agree nor disagree ☐<br>Slightly disagree ☐<br>Strongly disagree ☐
The stoma appliance was obvious to other people	Strongly agree ☐ 32<br>Slightly agree ☐<br>Neither agree nor disagree ☐<br>Slightly disagree ☐<br>Strongly disagree ☐
The color of the stoma appliance was discreet	Strongly agree ☐ 33<br>Slightly agree ☐<br>Neither agree nor disagree ☐<br>Slightly disagree ☐<br>Strongly disagree ☐

It was difficult to hide the stoma appliance because of ballooning — 34

- Strongly agree ☐
- Slightly agree ☐
- Neither agree nor disagree ☐
- Slightly disagree ☐
- Strongly disagree ☐

Comfort
In the last seven days... — 35

The stoma appliance was comfortable to wear — 36

- Strongly agree ☐
- Slightly agree ☐
- Neither agree nor disagree ☐
- Slightly disagree ☐
- Strongly disagree ☐

I was not concerned about skin irritation under the stoma appliance (for example feelings of burning, itching, pinching or pain) — 37

- Strongly agree ☐
- Slightly agree ☐
- Neither agree nor disagree ☐
- Slightly disagree ☐
- Strongly disagree ☐

It was uncomfortable to remove the stoma appliance from my body — 38

- Strongly agree ☐
- Slightly agree ☐
- Neither agree nor disagree ☐
- Slightly disagree ☐
- Strongly disagree ☐

I often forgot that I was wearing the stoma appliance — 39

- Strongly agree ☐
- Slightly agree ☐

	Neither agree nor disagree ☐<br>Slightly disagree ☐<br>Strongly disagree ☐	
The stoma appliance was comfortable as it fitted well to my body movements	Strongly agree ☐<br>Slightly agree ☐<br>Neither agree nor disagree ☐<br>Slightly disagree ☐<br>Strongly disagree ☐	40
The stoma appliance disrupted my sleep during the night	Strongly agree ☐<br>Slightly agree ☐<br>Neither agree nor disagree ☐<br>Slightly disagree ☐<br>Strongly disagree ☐	41
Confidence<br>In the last seven days...	______	42
I was confident that the stoma appliance would not leak	Strongly agree ☐<br>Slightly agree ☐<br>Neither agree nor disagree ☐<br>Slightly disagree ☐<br>Strongly disagree ☐	43
I worried that the stoma appliance would become loose from my body	Strongly agree ☐<br>Slightly agree ☐<br>Neither agree nor disagree ☐<br>Slightly disagree ☐	44

Strongly disagree ☐

I felt confident that I could spend the night away from home despite wearing the stoma appliance

Strongly agree ☐ 45
Slightly agree ☐
Neither agree nor disagree ☐
Slightly disagree ☐
Strongly disagree ☐

I was confident the stoma appliance would not cause any problems for me

Strongly agree ☐ 46
Slightly agree ☐
Neither agree nor disagree ☐
Slightly disagree ☐
Strongly disagree ☐

I felt confident to take part in physical activities (for example, sports) whilst wearing the stoma appliance

Strongly agree ☐ 47
Slightly agree ☐
Neither agree nor disagree ☐
Slightly disagree ☐
Strongly disagree ☐

I worried that the stoma appliance would make a rustling noise

Strongly agree ☐ 48
Slightly agree ☐
Neither agree nor disagree ☐
Slightly disagree ☐
Strongly disagree ☐

Social life and relationships
In the last seven days... ______________ 49

I worried that my family and friends felt awkward around me because of the stoma appliance	Strongly agree ☐<br>Slightly agree ☐<br>Neither agree nor disagree ☐<br>Slightly disagree ☐<br>Strongly disagree ☐	50
I felt my social life had been restricted because of the stoma appliance	Strongly agree ☐<br>Slightly agree ☐<br>Neither agree nor disagree ☐<br>Slightly disagree ☐<br>Strongly disagree ☐	51
I avoided close physical contact with family and friends because of the stoma appliance	Strongly agree ☐<br>Slightly agree ☐<br>Neither agree nor disagree ☐<br>Slightly disagree ☐<br>Strongly disagree ☐	52
I worried about whether I could have a relationship because of my stoma appliance	Strongly agree ☐<br>Slightly agree ☐<br>Neither agree nor disagree ☐<br>Slightly disagree ☐<br>Strongly disagree ☐	53
I worried about whether the stoma appliance would affect my sex life	Strongly agree ☐<br>Slightly agree ☐<br>Neither agree nor disagree ☐<br>Slightly disagree ☐<br>Strongly disagree ☐	54

	Field Name	Data Type	Units	Values	Pre-Filled Values	Include Field OID
1	Complications today	1		0 = No<br>1 = Yes		COMP_TODAY
2	Severity of complications	1		0 = No skin complications<br>1 = Very mild<br>2 = Mild<br>3 = Moderate<br>4 = Severe<br>5 = Very severe		COMP_IF_YES
3	LEAKAGE_HEADER	1				LEAKAGE_HEADER
4	LEAKAGE_QS_1	1		0 = All of the time<br>1 = Often<br>2 = Sometimes<br>3 = Rarely or never		LEAKAGE_QS_1
5	LEAKAGE_QS_2	1		0 = All of the time<br>1 = Often<br>2 = Sometimes<br>3 = Rarely or never		LEAKAGE_QS_2

Production Germany 14. Jan. 2020: Primary matrix
Folder: Paper CRF data
Form: Paper Evaluation visit 1
Generated On: 30 Sep 2020 12:31:31

	Field Name	Data Type	Units	Values	Pre-Filled Values	Include Field OID
6	LEAKAGE_Q1S_3			0 = All of the time<br>1 = Often<br>2 = Sometimes<br>3 = Rarely or never		LEAKAGE_QS_3
7	LEAKAGE_Q1S_4			0 = All of the time<br>1 = Often<br>2 = Sometimes<br>3 = Rarely or never		LEAKAGE_QS_4
8	LEAKAGE_Q1S_5			0 = All of the time<br>1 = Often<br>2 = Sometimes<br>3 = Rarely or never		LEAKAGE_QS_5
9	LEAKAGE_Q1S_6			0 = All of the time<br>1 = Often<br>2 = Sometimes<br>3 = Rarely or never		LEAKAGE_QS_6
10	LEAKAGE_Q1S_7			0 = All of the time		LEAKAGE_QS_7

	Field Name	Data Type	Units	Values	Pre-Filled Values	Include Field OID
				1 = Often<br>2 = Sometimes<br>3 = Rarely or never		
11	LEAKAGE_Q1S_8			0 = All of the time<br>1 = Often<br>2 = Sometimes<br>3 = Rarely or never		LEAKAGE_QS_8
12	LEAKAGE_Q1S_9			0 = All of the time<br>1 = Often<br>2 = Sometimes<br>3 = Rarely or never		LEAKAGE_QS_9
13	LEAKAGE_Q1S_10			0 = All of the time<br>1 = Often<br>2 = Sometimes<br>3 = Rarely or never		LEAKAGE_QS_10
14	LEAKAGE_H1EADER2					LEAKAGE_HEADER2

	Field Name	Data Type	Units	Values	Pre-Filled Values	Include Field OID
15	LEAKAGE_Q1S_11			0 = All of the time<br>1 = Often<br>2 = Sometimes<br>3 = Rarely or never<br>9 = Not applicable		LEAKAGE_QS_11
16	LEAKAGE_Q1S_12			0 = All of the time<br>1 = Often<br>2 = Sometimes<br>3 = Rarely or never<br>9 = Not applicable		LEAKAGE_QS_12
17	LEAKAGE_Q1S_13			0 = All of the time<br>1 = Often<br>2 = Sometimes<br>3 = Rarely or never<br>9 = Not applicable		LEAKAGE_QS_13
18	LEAKAGE_Q1S_14			0 = All of the time<br>1 = Often<br>2 = Sometimes		LEAKAGE_QS_14

	Field Name	Data Type	Units	Values	Pre-Filled Values	Include Field OID
				3 = Rarely or never<br>9 = Not applicable		
19	LEAKAGE_Q1 S_15			0 = All of the time<br>1 = Often<br>2 = Sometimes<br>3 = Rarely or never<br>9 = Not applicable		LEAKAGE_Q S_15
20	LEAKAGE_Q1 S_16			0 = All of the time<br>1 = Often<br>2 = Sometimes<br>3 = Rarely or never<br>9 = Not applicable		LEAKAGE_Q S_16
21	LEAKAGE_Q1 S_17			0 = All of the time<br>1 = Often<br>2 = Sometimes<br>3 = Rarely or never<br>9 = Not applicable		LEAKAGE_Q S_17

	Field Name	Data Type	Units	Values	Pre-Filled Values	Include Field OID
22	LEAKAGE_Q1S_18			0 = All of the time<br>1 = Often<br>2 = Sometimes<br>3 = Rarely or never<br>9 = Not applicable		LEAKAGE_QS_18
23	LEAKAGE_H1EADER3					LEAKAGE_HEADER3
24	LEAKAGE_Q1S_19			0 = All of the time<br>1 = Often<br>2 = Sometimes<br>3 = Rarely or never		LEAKAGE_QS_19
25	LEAKAGE_Q1S_20			0 = All of the time<br>1 = Often<br>2 = Sometimes<br>3 = Rarely or never		LEAKAGE_QS_20
26	LEAKAGE_Q1S_21			0 = All of the time<br>1 = Often<br>2 = Sometimes		LEAKAGE_QS_21

	Field Name	Data Type	Units	Values	Pre-Filled Values	Include Field OID
				3 = Rarely or never		
27	LEAKAGE_QS_22			0 = All of the time<br>1 = Often<br>2 = Sometimes<br>3 = Rarely or never		LEAKAGE_QS_22
28	QOLH1	1				QOLH1
29	QOL_QS_1	1		1 = Strongly agree<br>2 = Slightly agree<br>3 = Neither agree nor disagree<br>4 = Slightly disagree<br>5 = Strongly disagree		QOL_QS_1
30	QOL_QS_2	1		1 = Strongly agree<br>2 = Slightly agree		QOL_QS_2

	Field Name	Data Type	Units	Values	Pre-Filled Values	Include Field OID
				3 = Neither agree nor disagree<br>4 = Slightly disagree<br>5 = Strongly disagree		
31	QOL_QS_3	1		1 = Strongly agree<br>2 = Slightly agree<br>3 = Neither agree nor disagree<br>4 = Slightly disagree<br>5 = Strongly disagree		QOL_QS_3
32	QOL_QS_4	1		1 = Strongly agree<br>2 = Slightly agree<br>3 = Neither agree nor disagree<br>4 = Slightly disagree<br>5 = Strongly disagree		QOL_QS_4

	Field Name	Data Type	Units	Values	Pre-Filled Values	Include Field OID
33	QOL_QS_5	1		1 = Strongly agree<br>2 = Slightly agree<br>3 = Neither agree nor disagree<br>4 = Slightly disagree<br>5 = Strongly disagree		QOL_QS_5
34	QOL_QS_6	1		1 = Strongly agree<br>2 = Slightly agree<br>3 = Neither agree nor disagree<br>4 = Slightly disagree<br>5 = Strongly disagree		QOL_QS_6
35	QOLH2	1				QOLH2
36	QOL_QS_7	1		1 = Strongly agree		QOL_QS_7

	Field Name	Data Type	Units	Values	Pre-Filled Values	Include Field OID
				2 = Slightly agree<br>3 = Neither agree nor disagree<br>4 = Slightly disagree<br>5 = Strongly disagree		
37	QOL_QS_8	1		1 = Strongly agree<br>2 = Slightly agree<br>3 = Neither agree nor disagree<br>4 = Slightly disagree<br>5 = Strongly disagree		QOL_QS_8
38	QOL_QS_9	1		1 = Strongly agree<br>2 = Slightly agree<br>3 = Neither agree nor disagree<br>4 = Slightly disagree		QOL_QS_9

	Field Name	Data Type	Units	Values	Pre-Filled Values	Include Field OID
				5 = Strongly disagree		
39	QOL_QS_101			1 = Strongly agree<br>2 = Slightly agree<br>3 = Neither agree nor disagree<br>4 = Slightly disagree<br>5 = Strongly disagree		QOL_QS_10
40	QOL_QS_111			1 = Strongly agree<br>2 = Slightly agree<br>3 = Neither agree nor disagree<br>4 = Slightly disagree<br>5 = Strongly disagree		QOL_QS_11

	Field Name	Data Type	Units	Values	Pre-Filled Values	Include Field OID
41	QOL_QS_121			1 = Strongly agree<br>2 = Slightly agree<br>3 = Neither agree nor disagree<br>4 = Slightly disagree<br>5 = Strongly disagree		QOL_QS_12
42	QOLH3	1				QOLH3
43	QOL_QS_131			1 = Strongly agree<br>2 = Slightly agree<br>3 = Neither agree nor disagree<br>4 = Slightly disagree<br>5 = Strongly disagree		QOL_QS_13
44	QOL_QS_141			1 = Strongly agree		QOL_QS_14

	Field Name	Data Type	Units	Values	Pre-Filled Values	Include Field OID
				2 = Slightly agree<br>3 = Neither agree nor disagree<br>4 = Slightly disagree<br>5 = Strongly disagree		
45	QOL_QS_151			1 = Strongly agree<br>2 = Slightly agree<br>3 = Neither agree nor disagree<br>4 = Slightly disagree<br>5 = Strongly disagree		QOL_QS_15
46	QOL_QS_161			1 = Strongly agree<br>2 = Slightly agree<br>3 = Neither agree nor disagree<br>4 = Slightly disagree		QOL_QS_16

	Field Name	Data Type	Units	Values	Pre-Filled Values	Include Field OID
				5 = Strongly disagree		
47	QOL_QS_171			1 = Strongly agree<br>2 = Slightly agree<br>3 = Neither agree nor disagree<br>4 = Slightly disagree<br>5 = Strongly disagree		QOL_QS_17
48	QOL_QS_181			1 = Strongly agree<br>2 = Slightly agree<br>3 = Neither agree nor disagree<br>4 = Slightly disagree<br>5 = Strongly disagree		QOL_QS_18
49	QOLH4	1				QOLH4

	Field Name	Data Type	Units	Values	Pre-Filled Values	Include Field OID
50	QOL_QS_191			1 = Strongly agree<br>2 = Slightly agree<br>3 = Neither agree nor disagree<br>4 = Slightly disagree<br>5 = Strongly disagree		QOL_QS_19
51	QOL_QS_201			1 = Strongly agree<br>2 = Slightly agree<br>3 = Neither agree nor disagree<br>4 = Slightly disagree<br>5 = Strongly disagree		QOL_QS_20
52	QOL_QS_211			1 = Strongly agree<br>2 = Slightly agree<br>3 = Neither agree nor disagree		QOL_QS_21

	Field Name	Data Type	Units	Values	Pre-Filled Values	Include Field OID
				4 = Slightly disagree<br>5 = Strongly disagree		
53	QOL_QS_221			1 = Strongly agree<br>2 = Slightly agree<br>3 = Neither agree nor disagree<br>4 = Slightly disagree<br>5 = Strongly disagree		QOL_QS_22
54	QOL_QS_231			1 = Strongly agree<br>2 = Slightly agree<br>3 = Neither agree nor disagree<br>4 = Slightly disagree<br>5 = Strongly disagree		QOL_QS_23

Production Germany 14. Jan. 2020: Primary matrix
Folder: Product change TP1
Form: Type of change
Generated On: 30 Sep 2020 12:31:31

Product sequence number ______ (1)

What type of change do you want to register? Bag change ☐ (2)
Baseplate change ☐

______ (3)

Production Germany 14. Jan. 2020: Primary matrix
Folder: Product change TP1
Form: Type of change
Generated On: 30 Sep 2020 12:31:31

	Field Name	Data Type	Units	Values	Pre-Filled Values	Include Field OID
1	Product sequence number	3				PRODSEQUENCE
2	Type of change	$3		BAG = Bag change<br>BP = Baseplate change		CNG_TYPE
3	HIDDEN	$100		{ "Change_type": { "BAG": "CHANGETIME_BP", "BP": "CHANGETIME_BP" } }	1: { "Change_type": { "BAG": "CHANGETIME_BP", "BP": "CHANGETIME_BP" } }	HIDDEN

Production Germany 14. Jan. 2020: Primary matrix
Folder: Product change TP1
Form: Time for performing change of bag
Generated On: 30 Sep 2020 12:31:31

Product sequence number ______________________ (1)

Are you going to change now or have you changed previously without using the app? (2)

- [ ] I am going to change now
- [ ] I have changed previously without using the app (e.g. during the night)

---

(3)
{ "CNGnowprev": { "N": "BAGCNG", "P": "BAGCNGPREV" } }

Production Germany 14. Jan. 2020: Primary matrix
Folder: Product change TP1
Form: Time for performing change of bag
Generated On: 30 Sep 2020 12:31:31

	Field Name	Data Type	Units	Values	Pre-Filled Values	Include Field OID
1	Product sequence number	3				PRODSEQUENCE
2	Time for performing chance of bag	$1		N = I am going to change now P = I have changed previously without using the app (e.g. during the night)		CHANGETIME_BAG
3	HIDDEN	$100		{ "CNGnowprev": { "N": "BAGCNG", "P": "BAGCNGPREV" } }		HIDDEN

Production Germany 14. Jan. 2020: Primary matrix
Folder: Product change TP1
Form: Bag change previously
Generated On: 30 Sep 2020 12:31:31

Product sequence number ______________ ①

When did you change the bag (approximately)? ______________ ②

Production Germany 14. Jan. 2020: Primary matrix
Folder: Product change TP1
Form: Bag change previously
Generated On: 30 Sep 2020 12:31:31

	Field Name	Data Type	Units	Values	Pre-Filled Values	Include Field OID
1	Product sequence number	3				PRODSEQUENCE
2	When did you change the bag	dd MMM yyyy HH:nn				CNGPREVDTC

Production Germany 14. Jan. 2020: Primary matrix
Folder: Product change TP1
Form: Bag change
Generated On: 30 Sep 2020 12:31:31

Product sequence number	______	1
Photo of the baseplate on the skin	______	2
Baseplate on skin photo file name	______	3
Baseplate on skin photo date and time	______	4

Production Germany 14. Jan. 2020: Primary matrix
Folder: Product change TP1
Form: Bag change
Generated On: 30 Sep 2020 12:31:31

	Field Name	Data Type	Units	Values	Pre-Filled Values	Include Field OID
1	Product sequence number	3				PRODSEQUENCE
2	Photo of the baseplate on the skin	1				PHOTO_BPONSKIN
3	Photo file name	$100				BPONSKIN_PHOTO
4	Baseplate on skin photo	dd MMM yyyy HH:nn				PHT03DTC

Production Germany 14. Jan. 2020: Primary matrix
Folder: Product change TP1
Form: Time for performing change of BP
Generated On: 30 Sep 2020 12:31:31

Product sequence number ________________ (1)

Are you going to change now or have you changed previously?

- I am going to change now ☐ (2)
- I have changed previously without using the app (e.g. during the night) ☐

________________ (3)

Production Germany 14. Jan. 2020: Primary matrix
Folder: Product change TP1
Form: Time for performing change of BP
Generated On: 30 Sep 2020 12:31:31

	Field Name	Data Type	Units	Values	Pre-Filled Values	Include Field OID
1	Product sequence number	3				PRODSEQUENCE
2	Time for performing change of baseplate	$1		N = I am going to change now<br>P = I have changed previously without using the app (e.g. during the night)		CHANGETIME_BP
3	HIDDEN	$100		{ "CNGnowprev": { "N": "BPCNG", "P": "BPCNGPREV" } }	1: { "CNGnowprev": { "N": "BPCNG", "P": "BPCNGPREV" } }	HIDDEN

Production Germany 14. Jan. 2020: Primary matrix
Folder: Product change TP1
Form: Baseplate change previously
Generated On: 30 Sep 2020 12:31:31

Product sequence number ____________________ ①

When did you change the product (approximately)? ____________________ ②

Production Germany 14. Jan. 2020: Primary matrix
Folder: Product change TP1
Form: Baseplate change previously
Generated On: 30 Sep 2020 12:31:31

	Field Name	Data Type	Units	Values	Pre-Filled Values	Include Field OID
1	Product sequence number	3				PRODSEQUENCE
2	Product change previously	dd MMM yyyy HH:nn				CNGPREVDTC

Production Germany 14. Jan. 2020: Primary matrix
Folder: Product change TP1
Form: Baseplate change TP 1
Generated On: 30 Sep 2020 12:31:31

Product sequence number	________	1
Photo of the stoma	________	2
Stoma photo file name	________	3
Stoma photo date and time	________	4
Photo of the product	________	5
Product photo file name	________	6
Product photo date and time	________	7
Do you experience any bleeding from the skin around your stoma right now when changing your product?	Not experiencing ☐<br>Experiencing ☐	8
Once you have cleaned and dried the skin, do you still experience any weeping or moisture on the skin around your stoma right now when changing your product?	Not experiencing ☐<br>Experiencing ☐	9
Are you experiencing any ulcers or sores around your stoma right now when changing your product?	Not experiencing ☐<br>Experiencing ☐	10
How much of the time have you experienced any itching around your stoma since you last changed your product?	None of the time ☐<br>A little of the time ☐<br>Some of the time ☐<br>A lot of the time ☐<br>All of the time ☐	11

Please rate on a scale from 0-10 how itchy the skin around your stoma has been at its worst since you last changed your product

12

- 0 No itch ☐
- 1 Very mild itch ☐
- 2 ☐
- 3 ☐
- 4 ☐
- 5 ☐
- 6 ☐
- 7 ☐
- 8 ☐
- 9 ☐
- 10 Worst possible peristomal skin itch ☐

How much of the time have you experienced any pain around your stoma since you last changed your product?

13

- None of the time ☐
- A little of the time ☐
- Some of the time ☐
- A lot of the time ☐
- All of the time ☐

Please rate on a scale from 0-10 how painful the skin around your stoma has been at its worst since you last changed your product

14

- 0 No pain ☐
- 1 Very mild pain ☐
- 2 ☐
- 3 ☐
- 4 ☐
- 5 ☐
- 6 ☐
- 7 ☐
- 8 ☐
- 9 ☐
- 10 Worst possible peristomal skin pain ☐

How much of the time have you experienced any burning feelings from the skin around your stoma since you last changed your product?

- None of the time ▢ 15
- A little of the time ▢
- Some of the time ▢
- A lot of the time ▢
- All of the time ▢

Please rate on a scale from 0-10 any burning feelings from the skin around your stoma at its worst since you last changed your product

- 0 No burning ▢ 16
- 1 Very mild burning ▢
- 2 ▢
- 3 ▢
- 4 ▢
- 5 ▢
- 6 ▢
- 7 ▢
- 8 ▢
- 9 ▢
- 10 Worst possible peristomal skin burning ▢

How would you rate on a scale of 0-10 your overall physical discomfort from the skin around your stoma since you last changed your product?

- 0 No discomfort ▢ 17
- 1 ▢
- 2 ▢
- 3 ▢
- 4 ▢
- 5 ▢
- 6 ▢
- 7 ▢
- 8 ▢
- 9 ▢
- 10 A lot of discomfort ▢

18

On a scale of 0-10, how much have skin complications around your stoma caused you difficulty in your overall ability to move around since you last changed your product? For example, bending or walking

- No skin complications ☐
- 0 No difficulty ☐
- 1 ☐
- 2 ☐
- 3 ☐
- 4 ☐
- 5 ☐
- 6 ☐
- 7 ☐
- 8 ☐
- 9 ☐
- 10 A lot of difficulty ☐

19

On a scale of 0-10, how much have skin complications around your stoma caused you difficulty in your overall ability to stick the stoma bag adhesive to your skin since you last changed your product?

- No skin complications ☐
- 0 No difficulty ☐
- 1 ☐
- 2 ☐
- 3 ☐
- 4 ☐
- 5 ☐
- 6 ☐
- 7 ☐
- 8 ☐
- 9 ☐
- 10 A lot of difficulty ☐

20

What was the main reason for change of the baseplate? ____________________

21

I followed my usual changing pattern ____________________

Production Germany 14. Jan. 2020: Primary matrix
Folder: Product change TP1
Form: Baseplate change TP 1
Generated On: 30 Sep 2020 12:31:31

I thought it would be nice with a clean product	______	22
In preparation of an activity (i.e. going out, doing sports, travelling)	______	23
The entire baseplate had become detached	______	24
The outer edge of the baseplate had become detached	______	25
The center of the baseplate had become detached	______	26
I was afraid the baseplate would become detached	______	27
The area around the stoma was itching	______	28
The area around the stoma was painful	______	29
There was leakage underneath and outside the baseplate	______	30
There was leakage underneath the baseplate (but not outside the baseplate)	______	31
The bag was full of air (ballooning)	______	32
There was a vacuum in the bag (pancaking)	______	33
Due to visit in the investigation	______	34
Other reason	______	35

If other reason for change is selected, please specify ____________ 36

Did you experience leakage outside the baseplate (i.e. soiling the clothes)? 37

- No ☐
- Yes ☐

Did you experience the bag becoming overfilled with air during use (ballooning)? 38

- Not at all ☐
- A little ☐
- Some ☐
- Much ☐
- Very much ☐

Which stoma accessories did you use?

None ____________ 40

Adhesive remover ____________ 41

Paste ____________ 42

Rings ____________ 43

Ostomy tape ____________ 44

Ostomy belt ____________ 45

Hernia belt ____________ 46

Stoma powder ____________ 47

Production Germany 14. Jan. 2020: Primary matrix
Folder: Product change TP1
Form: Baseplate change TP 1
Generated On: 30 Sep 2020 12:31:31

Barrier lotion	______	48
Barrier cream	______	49
Barrier spray	______	50
Barrier wipes	______	51
Cleansing wipes/cleansing spray	______	52
Odour remover	______	53
Other accessories	______	54
If 'Other' please specify:	______	55

Production Germany 14. Jan. 2020: Primary matrix
Folder: Product change TP1
Form: Baseplate change TP 1
Generated On: 30 Sep 2020 12:31:31

	Field Name	Data Type	Units	Values	Pre-Filled Values	Include Field OID
1	Product sequence number	3				PRODSEQU ENCE
2	Photo of the stoma	1				PHOTO_SKI N
3	Stoma photo name	$100				PHT0101
4	Stoma photo date and time	dd MMM yyyy HH:nn				PHT01DTC
5	Photo of the product	1				PHOTO_PR ODUCT
6	Product photo name	$100				PROD_PHO TO
7	Product photo date and time	dd MMM yyyy HH:nn				PHT02DTC
8	Ostomy Skin Tool 1	1		0 = Not experiencin g<br>1 = Experiencin g		OST_1

Production Germany 14. Jan. 2020: Primary matrix
Folder: Product change TP1
Form: Baseplate change TP 1
Generated On: 30 Sep 2020 12:31:31

	Field Name	Data Type	Units	Values	Pre-Filled Values	Include Field OID
9	Ostomy Skin Tool 2	1		0 = Not experiencing<br>1 = Experiencing		OST_2
10	Ostomy Skin Tool 3	1		0 = Not experiencing<br>1 = Experiencing		OST_3
11	Ostomy Skin Tool 4	1		1 = None of the time<br>2 = A little of the time<br>3 = Some of the time<br>4 = A lot of the time<br>5 = All of the time		OST_4
12	Ostomy Skin Tool 5	2		0 = 0 No itch<br>1 = 1 Very mild itch<br>2 = 2<br>3 = 3<br>4 = 4<br>5 = 5<br>6 = 6<br>7 = 7		LONGSCALE_OST_5

	Field Name	Data Type	Units	Values	Pre-Filled Values	Include Field OID
				8 = 8<br>9 = 9<br>10 = 10 Worst possible peristomal skin itch		
13	Ostomy Skin Tool 6	1		1 = None of the time<br>2 = A little of the time<br>3 = Some of the time<br>4 = A lot of the time<br>5 = All of the time		OST_6
14	Ostomy Skin Tool 7	2		0 = 0 No pain<br>1 = 1 Very mild pain<br>2 = 2<br>3 = 3<br>4 = 4<br>5 = 5<br>6 = 6<br>7 = 7<br>8 = 8<br>9 = 9<br>10 = 10 Worst possible peristomal skin pain		LONGSCALE_OST_7

Production Germany 14. Jan. 2020: Primary matrix
Folder: Product change TP1
Form: Baseplate change TP 1
Generated On: 30 Sep 2020 12:31:31

	Field Name	Data Type	Units	Values	Pre-Filled Values	Include Field OID
15	Ostomy Skin Tool 8	1		1 = None of the time<br>2 = A little of the time<br>3 = Some of the time<br>4 = A lot of the time<br>5 = All of the time		OST_8
16	Ostomy Skin Tool 9	2		0 = 0 No burning<br>1 = 1 Very mild burning<br>2 = 2<br>3 = 3<br>4 = 4<br>5 = 5<br>6 = 6<br>7 = 7<br>8 = 8<br>9 = 9<br>10 = 10 Worst possible peristomal skin burning		LONGSCALE_OST_9
17	Ostomy Skin Tool 10	2		0 = 0 No discomfort<br>1 = 1<br>2 = 2<br>3 = 3<br>4 = 4		LONGSCALE_OST_10

	Field Name	Data Type	Units	Values	Pre-Filled Values	Include Field OID
				5 = 5<br>6 = 6<br>7 = 7<br>8 = 8<br>9 = 9<br>10 = 10 A lot of discomfort		
18	Ostomy Skin Tool 11	2		99 = No skin complications<br>0 = 0 No difficulty<br>1 = 1<br>2 = 2<br>3 = 3<br>4 = 4<br>5 = 5<br>6 = 6<br>7 = 7<br>8 = 8<br>9 = 9<br>10 = 10 A lot of difficulty		LONGSCALE _OST_11
19	Ostomy Skin Tool 12	2		99 = No skin complications<br>0 = 0 No difficulty<br>1 = 1<br>2 = 2		LONGSCALE _OST_12

	Field Name	Data Type	Units	Values	Pre-Filled Values	Include Field OID
				3 = 3<br>4 = 4<br>5 = 5<br>6 = 6<br>7 = 7<br>8 = 8<br>9 = 9<br>10 = 10 A lot of difficulty		
20	MULTI_CNG_HEADER	1				MULTI_CNG_HEADER
21	Reason for change no. 1	1				MULTI_CNG_1
22	Reason for change no. 2	1				MULTI_CNG_2
23	Reason for change no. 3	1				MULTI_CNG_3
24	Reason for change no. 4	1				MULTI_CNG_4

Production Germany 14. Jan. 2020: Primary matrix
Folder: Product change TP1
Form: Baseplate change TP 1
Generated On: 30 Sep 2020 12:31:31

	Field Name	Data Type	Units	Values	Pre-Filled Values	Include Field OID
25	Reason for change no. 5	1				MULTI_CNG _5
26	Reason for change no. 6	1				MULTI_CNG _6
27	Reason for change no. 7	1				MULTI_CNG _7
28	Reason for change no. 8	1				MULTI_CNG _8
29	Reason for change no. 9	1				MULTI_CNG _9
30	Reason for change no. 10	1				MULTI_CNG _10
31	Reason for change no. 11	1				MULTI_CNG _11
32	Reason for change no. 12	1				MULTI_CNG _12

	Field Name	Data Type	Units	Values	Pre-Filled Values	Include Field OID
33	Reason for change no. 13	1				MULTI_CNG _13
34	Reason for change no. 14	1				MULTI_CNG _14
35	Reason for change no. 15	1				MULTI_CNG _15
36	Description of other reason for change	$100				MULTI_CNG _OTHER
37	Leakage outside	1		0 = No<br>1 = Yes		LEAKAGE_O UTSIDE
38	Degree of ballooning	1		1 = Not at all<br>2 = A little<br>3 = Some<br>4 = Much<br>5 = Very much		BALLOONIN G_DEGREE
40	Accessories 1	1				MULTI_ACC _1

Production Germany 14. Jan. 2020: Primary matrix
Folder: Product change TP1
Form: Baseplate change TP 1
Generated On: 30 Sep 2020 12:31:31

	Field Name	Data Type	Units	Values	Pre-Filled Values	Include	Field OID
41	Accessories 12						MULTI_ACC_2
42	Accessories 13						MULTI_ACC_3
43	Accessories 14						MULTI_ACC_4
44	Accessories 15						MULTI_ACC_5
45	Accessories 16						MULTI_ACC_6
46	Accessories 17						MULTI_ACC_7
47	Accessories 18						MULTI_ACC_8
48	Accessories 19						MULTI_ACC_9
49	Accessories 110						MULTI_ACC_10
50	Accessories 111						MULTI_ACC_11

Production Germany 14. Jan. 2020: Primary matrix
Folder: Product change TP1
Form: Baseplate change TP 1
Generated On: 30 Sep 2020 12:31:31

	Field Name	Data Type	Units	Values	Pre-Filled Values	Include Field OID
51	Accessories 12	1				MULTI_ACC_12
52	Accessories 13	1				MULTI_ACC_13
53	Accessories 14	1				MULTI_ACC_14
54	Accessories 15	1				MULTI_ACC_15
55	Accessories other	$50				MULTI_ACC_OTHER

Do you have any skin complications around your stoma today? (1)

- No ☐
- Yes ☐

If yes, how would you describe the skin complications around your stoma today? (2)

- No skin complications ☐
- Very mild ☐
- Mild ☐
- Moderate ☐
- Severe ☐
- Very severe ☐

How often have you experienced itchy skin around your stoma in the past 14 days? (3)

- None of the time ☐
- A little of the time ☐
- Some of the time ☐
- A lot of the time ☐
- All of the time ☐

How often have you experienced any pain in the skin around the stoma in the past 14 days? (4)

- None of the time ☐
- A little of the time ☐
- Some of the time ☐
- A lot of the time ☐
- All of the time ☐

How often have you experienced burning feelings in the skin around your stoma in the past 14 days? (5)

- None of the time ☐
- A little of the time ☐
- Some of the time ☐
- A lot of the time ☐
- All of the time ☐

Compared to the beginning of this test period (Test Period 1) how have any skin complications around your stoma changed? (6)

- Very much improved ☐
- Much improved ☐
- A little improved ☐
- No change ☐
- A little worse ☐
- Much worse ☐
- Very much worse ☐

Did your stoma behave differently from normal since your last visit/call? (7)

- No ☐
- Yes ☐

If yes, please describe (8) ______________________

Emotional impact. In the last seven days, due to leakage or worry about leakage...
(please tick the box to continue) (9) ______________________

I felt panic (10)

- All of the time ☐
- Often ☐
- Sometimes ☐
- Rarely or never ☐

I felt stressed out (11)

- All of the time ☐
- Often ☐
- Sometimes ☐
- Rarely or never ☐

I felt more afraid about leaks in the future (12)

- All of the time ☐
- Often ☐

Sometimes ☐
Rarely or never ☐

I felt worry

All of the time ☐ 13
Often ☐
Sometimes ☐
Rarely or never ☐

I felt frustrated

All of the time ☐ 14
Often ☐
Sometimes ☐
Rarely or never ☐

I felt embarrassed

All of the time ☐ 15
Often ☐
Sometimes ☐
Rarely or never ☐

I felt worried that I might leak

All of the time ☐ 16
Often ☐
Sometimes ☐
Rarely or never ☐

I couldn't sleep

All of the time ☐ 17
Often ☐
Sometimes ☐
Rarely or never ☐

I kept waking up at night to check my stoma — 18

- All of the time ☐
- Often ☐
- Sometimes ☐
- Rarely or never ☐

I kept checking my ostomy bag to see if I have leaked — 19

- All of the time ☐
- Often ☐
- Sometimes ☐
- Rarely or never ☐

Usual and social activities.
In the last seven days due to leakage or worry about leakage... (please tick to continue) — 20

____

I decided to stay at home — 21

- All of the time ☐
- Often ☐
- Sometimes ☐
- Rarely or never ☐
- Not applicable ☐

I couldn't do light activities — 22

- All of the time ☐
- Often ☐
- Sometimes ☐
- Rarely or never ☐
- Not applicable ☐

I changed my plans — 23

- All of the time ☐
- Often ☐
- Sometimes ☐

Rarely or never ☐
Not applicable ☐

I was unable to go out and meet family and friends

All of the time ☐ 24
Often ☐
Sometimes ☐
Rarely or never ☐
Not applicable ☐

I avoided close physical contact with family and friends

All of the time ☐ 25
Often ☐
Sometimes ☐
Rarely or never ☐
Not applicable ☐

I did not want to see people

All of the time ☐ 26
Often ☐
Sometimes ☐
Rarely or never ☐
Not applicable ☐

I avoided people

All of the time ☐ 27
Often ☐
Sometimes ☐
Rarely or never ☐
Not applicable ☐

I tried to avoid meeting new people

All of the time ☐ 28
Often ☐

Sometimes ☐
Rarely or never ☐
Not applicable ☐

Coping and in control.
In the last 7 days, due to leakage or worry about leakage... 29

I felt in control 30

All of the time ☐
Often ☐
Sometimes ☐
Rarely or never ☐

I was able to cope 31

All of the time ☐
Often ☐
Sometimes ☐
Rarely or never ☐

I felt calm 32

All of the time ☐
Often ☐
Sometimes ☐
Rarely or never ☐

I saw my friends as I usually do 33

All of the time ☐
Often ☐
Sometimes ☐
Rarely or never ☐

Discreetness.
In the last seven days... 34

It was difficult to hide the stoma appliance under clothing 35

- Strongly agree ☐
- Slightly agree ☐
- Neither agree nor disagree ☐
- Slightly disagree ☐
- Strongly disagree ☐

I was self-conscious about the appearance of the stoma appliance 36

- Strongly agree ☐
- Slightly agree ☐
- Neither agree nor disagree ☐
- Slightly disagree ☐
- Strongly disagree ☐

The stoma appliance limited the choice of clothes that I could wear 37

- Strongly agree ☐
- Slightly agree ☐
- Neither agree nor disagree ☐
- Slightly disagree ☐
- Strongly disagree ☐

The stoma appliance was obvious to other people 38

- Strongly agree ☐
- Slightly agree ☐
- Neither agree nor disagree ☐
- Slightly disagree ☐
- Strongly disagree ☐

The color of the stoma appliance was discreet 39

- Strongly agree ☐
- Slightly agree ☐

	Neither agree nor disagree ☐<br>Slightly disagree ☐<br>Strongly disagree ☐
It was difficult to hide the stoma appliance because of ballooning	Strongly agree ☐ 40<br>Slightly agree ☐<br>Neither agree nor disagree ☐<br>Slightly disagree ☐<br>Strongly disagree ☐
Comfort.<br>In the last seven days...	41
The stoma appliance was comfortable to wear	Strongly agree ☐ 42<br>Slightly agree ☐<br>Neither agree nor disagree ☐<br>Slightly disagree ☐<br>Strongly disagree ☐
I was not concerned about skin irritation under the stoma appliance (for example feelings of burning, itching, pinching or pain)	Strongly agree ☐ 43<br>Slightly agree ☐<br>Neither agree nor disagree ☐<br>Slightly disagree ☐<br>Strongly disagree ☐
It was uncomfortable to remove the stoma appliance from my body	Strongly agree ☐ 44<br>Slightly agree ☐<br>Neither agree nor disagree ☐<br>Slightly disagree ☐

Strongly disagree ▢

I often forgot that I was wearing the stoma appliance 45

- Strongly agree ▢
- Slightly agree ▢
- Neither agree nor disagree ▢
- Slightly disagree ▢
- Strongly disagree ▢

The stoma appliance was comfortable as it fitted well to my body movements 46

- Strongly agree ▢
- Slightly agree ▢
- Neither agree nor disagree ▢
- Slightly disagree ▢
- Strongly disagree ▢

The stoma appliance disrupted my sleep during the night 47

- Strongly agree ▢
- Slightly agree ▢
- Neither agree nor disagree ▢
- Slightly disagree ▢
- Strongly disagree ▢

Confidence.
In the last seven days... 48

I was confident that the stoma appliance would not leak 49

- Strongly agree ▢
- Slightly agree ▢
- Neither agree nor disagree ▢
- Slightly disagree ▢
- Strongly disagree ▢

I worried that the stoma appliance would become loose from my body

50

- Strongly agree ☐
- Slightly agree ☐
- Neither agree nor disagree ☐
- Slightly disagree ☐
- Strongly disagree ☐

I felt confident that I could spend the night away from home despite wearing the stoma appliance

51

- Strongly agree ☐
- Slightly agree ☐
- Neither agree nor disagree ☐
- Slightly disagree ☐
- Strongly disagree ☐

I was confident the stoma appliance would not cause any problems for me

52

- Strongly agree ☐
- Slightly agree ☐
- Neither agree nor disagree ☐
- Slightly disagree ☐
- Strongly disagree ☐

I felt confident to take part in physical activities (for example, sports) whilst wearing the stoma appliance

53

- Strongly agree ☐
- Slightly agree ☐
- Neither agree nor disagree ☐
- Slightly disagree ☐
- Strongly disagree ☐

I worried that the stoma appliance would make a rustling noise

54

- Strongly agree ☐
- Slightly agree ☐
- Neither agree nor disagree ☐
- Slightly disagree ☐
- Strongly disagree ☐

Social life and relationships.
In the last seven days... 55

I worried that my family and friends felt awkward around me because of the stoma appliance 56

- Strongly agree
- Slightly agree
- Neither agree nor disagree
- Slightly disagree
- Strongly disagree

I felt my social life had been restricted because of the stoma appliance 57

- Strongly agree
- Slightly agree
- Neither agree nor disagree
- Slightly disagree
- Strongly disagree

I avoided close physical contact with family and friends because of the stoma appliance 58

- Strongly agree
- Slightly agree
- Neither agree nor disagree
- Slightly disagree
- Strongly disagree

I worried about whether I could have a relationship because of my stoma appliance 59

- Strongly agree
- Slightly agree
- Neither agree nor disagree
- Slightly disagree
- Strongly disagree

I worried about whether the stoma appliance would affect my sex life 60

- Strongly agree
- Slightly agree

- Neither agree nor disagree ☐
- Slightly disagree ☐
- Strongly disagree ☐

How was the feeling of security while wearing the product? (61)

- Very poor ☐
- Poor ☐
- Neither poor nor good ☐
- Good ☐
- Very good ☐

How did you find cutting the baseplate? (62)

- Very difficult ☐
- Difficult ☐
- Neither difficult nor easy ☐
- Easy ☐
- Very easy ☐

How did you find applying the baseplate? (63)

- Very difficult ☐
- Difficult ☐
- Neither difficult nor easy ☐
- Easy ☐
- Very easy ☐

How did you find removing the baseplate? (64)

- Very difficult ☐
- Difficult ☐
- Neither difficult nor easy ☐
- Easy ☐
- Very easy ☐

Production Germany 14. Jan. 2020: Primary matrix
Folder: Final evaluation TP1
Form: Final evaluation TP 1
Generated On: 30 Sep 2020 12:31:31

	Field Name	Data Type	Units	Values	Pre-Filled Values	Include Field OID
1	Complications today	1		0 = No<br>1 = Yes		COMP_TODAY
2	Severity of complications	1		0 = No skin complications<br>1 = Very mild<br>2 = Mild<br>3 = Moderate<br>4 = Severe<br>5 = Very severe		COMP_IF_YES
3	Itching in 14 days	1		1 = None of the time<br>2 = A little of the time<br>3 = Some of the time<br>4 = A lot of the time<br>5 = All of the time		ITCHING_14DAYS
4	Pain in 14 days	1		1 = None of the time<br>2 = A little of the time<br>3 = Some of the time<br>4 = A lot of the time		PAIN_14DAYS

	Field Name	Data Type	Units	Values	Pre-Filled Values	Include Field OID
				5 = All of the time		
5	Burning in 14 days	1		1 = None of the time<br>2 = A little of the time<br>3 = Some of the time<br>4 = A lot of the time<br>5 = All of the time		BURNING_14DAYS
6	Own comparison of skin	1		1 = Very much improved<br>2 = Much improved<br>3 = A little improved<br>4 = No change<br>5 = A little worse<br>6 = Much worse<br>7 = Very much worse		COMPARE_OWN
7	Stoma behaviour	1		0 = No<br>1 = Yes		STOMABEHAVE_YN

	Field Name	Data Type	Units	Values	Pre-Filled Values	Include Field OID
8	Different stoma behaviour	$200				BEHAVE_IF_YES
9	LEAKAGE_HEADER	1				LEAKAGE_HEADER
10	LEAKAGE_QS_1	1		0 = All of the time<br>1 = Often<br>2 = Sometimes<br>3 = Rarely or never		LEAKAGE_QS_1
11	LEAKAGE_QS_2	1		0 = All of the time<br>1 = Often<br>2 = Sometimes<br>3 = Rarely or never		LEAKAGE_QS_2
12	LEAKAGE_QS_3	1		0 = All of the time<br>1 = Often<br>2 = Sometimes<br>3 = Rarely or never		LEAKAGE_QS_3
13	LEAKAGE_QS_4	1		0 = All of the time		LEAKAGE_QS_4

	Field Name	Data Type	Units	Values	Pre-Filled Values	Include Field OID
				1 = Often<br>2 = Sometimes<br>3 = Rarely or never		
14	LEAKAGE_Q1S_5			0 = All of the time<br>1 = Often<br>2 = Sometimes<br>3 = Rarely or never		LEAKAGE_QS_5
15	LEAKAGE_Q1S_6			0 = All of the time<br>1 = Often<br>2 = Sometimes<br>3 = Rarely or never		LEAKAGE_QS_6
16	LEAKAGE_Q1S_7			0 = All of the time<br>1 = Often<br>2 = Sometimes<br>3 = Rarely or never		LEAKAGE_QS_7
17	LEAKAGE_Q1S_8			0 = All of the time<br>1 = Often		LEAKAGE_QS_8

	Field Name	Data Type	Units	Values	Pre-Filled Values	Include Field OID
				2 = Sometimes 3 = Rarely or never		
18	LEAKAGE_Q1 S_9			0 = All of the time 1 = Often 2 = Sometimes 3 = Rarely or never		LEAKAGE_Q S_9
19	LEAKAGE_Q1 S_10			0 = All of the time 1 = Often 2 = Sometimes 3 = Rarely or never		LEAKAGE_Q S_10
20	LEAKAGE_H1 EADER2					LEAKAGE_H EADER2
21	LEAKAGE_Q1 S_11			0 = All of the time 1 = Often 2 = Sometimes 3 = Rarely or never 9 = Not applicable		LEAKAGE_Q S_11

Production Germany 14. Jan. 2020: Primary matrix
Folder: Final evaluation TP1
Form: Final evaluation TP 1
Generated On: 30 Sep 2020 12:31:31

	Field Name	Data Type	Units	Values	Pre-Filled Values	Include Field OID
22	LEAKAGE_Q1 S_12			0 = All of the time<br>1 = Often<br>2 = Sometimes<br>3 = Rarely or never<br>9 = Not applicable		LEAKAGE_Q S_12
23	LEAKAGE_Q1 S_13			0 = All of the time<br>1 = Often<br>2 = Sometimes<br>3 = Rarely or never<br>9 = Not applicable		LEAKAGE_Q S_13
24	LEAKAGE_Q1 S_14			0 = All of the time<br>1 = Often<br>2 = Sometimes<br>3 = Rarely or never<br>9 = Not applicable		LEAKAGE_Q S_14
25	LEAKAGE_Q1 S_15			0 = All of the time<br>1 = Often<br>2 = Sometimes		LEAKAGE_Q S_15

	Field Name	Data Type	Units	Values	Pre-Filled Values	Include Field OID
				3 = Rarely or never<br>9 = Not applicable		
26	LEAKAGE_Q1 S_16			0 = All of the time<br>1 = Often<br>2 = Sometimes<br>3 = Rarely or never<br>9 = Not applicable		LEAKAGE_Q S_16
27	LEAKAGE_Q1 S_17			0 = All of the time<br>1 = Often<br>2 = Sometimes<br>3 = Rarely or never<br>9 = Not applicable		LEAKAGE_Q S_17
28	LEAKAGE_Q1 S_18			0 = All of the time<br>1 = Often<br>2 = Sometimes<br>3 = Rarely or never<br>9 = Not applicable		LEAKAGE_Q S_18

Production Germany 14. Jan. 2020: Primary matrix
Folder: Final evaluation TP1
Form: Final evaluation TP 1
Generated On: 30 Sep 2020 12:31:31

	Field Name	Data Type	Units	Values	Pre-Filled Values	Include Field OID
29	LEAKAGE_H1 EADER3					LEAKAGE_H EADER3
30	LEAKAGE_Q1 S_19			0 = All of the time 1 = Often 2 = Sometimes 3 = Rarely or never		LEAKAGE_Q S_19
31	LEAKAGE_Q1 S_20			0 = All of the time 1 = Often 2 = Sometimes 3 = Rarely or never		LEAKAGE_Q S_20
32	LEAKAGE_Q1 S_21			0 = All of the time 1 = Often 2 = Sometimes 3 = Rarely or never		LEAKAGE_Q S_21
33	LEAKAGE_Q1 S_22			0 = All of the time 1 = Often 2 = Sometimes 3 = Rarely or never		LEAKAGE_Q S_22

	Field Name	Data Type	Units	Values	Pre-Filled Values	Include Field OID
34	QOLH1	1				QOLH1
35	QOL_QS_1	1		1 = Strongly agree<br>2 = Slightly agree<br>3 = Neither agree nor disagree<br>4 = Slightly disagree<br>5 = Strongly disagree		QOL_QS_1
36	QOL_QS_2	1		1 = Strongly agree<br>2 = Slightly agree<br>3 = Neither agree nor disagree<br>4 = Slightly disagree<br>5 = Strongly disagree		QOL_QS_2
37	QOL_QS_3	1		1 = Strongly agree		QOL_QS_3

	Field Name	Data Type	Units	Values	Pre-Filled Values	Include Field OID
				2 = Slightly agree<br>3 = Neither agree nor disagree<br>4 = Slightly disagree<br>5 = Strongly disagree		
38	QOL_QS_4	1		1 = Strongly agree<br>2 = Slightly agree<br>3 = Neither agree nor disagree<br>4 = Slightly disagree<br>5 = Strongly disagree		QOL_QS_4
39	QOL_QS_5	1		1 = Strongly agree<br>2 = Slightly agree<br>3 = Neither agree nor disagree<br>4 = Slightly disagree		QOL_QS_5

	Field Name	Data Type	Units	Values	Pre-Filled Values	Include Field OID
				5 = Strongly disagree		
40	QOL_QS_6	1		1 = Strongly agree<br>2 = Slightly agree<br>3 = Neither agree nor disagree<br>4 = Slightly disagree<br>5 = Strongly disagree		QOL_QS_6
41	QOLH2	1				QOLH2
42	QOL_QS_7	1		1 = Strongly agree<br>2 = Slightly agree<br>3 = Neither agree nor disagree<br>4 = Slightly disagree<br>5 = Strongly disagree		QOL_QS_7

	Field Name	Data Type	Units	Values	Pre-Filled Values	Include Field OID
43	QOL_QS_8	1		1 = Strongly agree<br>2 = Slightly agree<br>3 = Neither agree nor disagree<br>4 = Slightly disagree<br>5 = Strongly disagree		QOL_QS_8
44	QOL_QS_9	1		1 = Strongly agree<br>2 = Slightly agree<br>3 = Neither agree nor disagree<br>4 = Slightly disagree<br>5 = Strongly disagree		QOL_QS_9
45	QOL_QS_10	1		1 = Strongly agree<br>2 = Slightly agree<br>3 = Neither agree nor disagree		QOL_QS_10

	Field Name	Data Type	Units	Values	Pre-Filled Values	Include Field OID
				4 = Slightly disagree<br>5 = Strongly disagree		
46	QOL_QS_111			1 = Strongly agree<br>2 = Slightly agree<br>3 = Neither agree nor disagree<br>4 = Slightly disagree<br>5 = Strongly disagree		QOL_QS_11
47	QOL_QS_121			1 = Strongly agree<br>2 = Slightly agree<br>3 = Neither agree nor disagree<br>4 = Slightly disagree<br>5 = Strongly disagree		QOL_QS_12

	Field Name	Data Type	Units	Values	Pre-Filled Values	Include Field OID
48	QOLH3	1				QOLH3
49	QOL_QS_131			1 = Strongly agree<br>2 = Slightly agree<br>3 = Neither agree nor disagree<br>4 = Slightly disagree<br>5 = Strongly disagree		QOL_QS_13
50	QOL_QS_141			1 = Strongly agree<br>2 = Slightly agree<br>3 = Neither agree nor disagree<br>4 = Slightly disagree<br>5 = Strongly disagree		QOL_QS_14
51	QOL_QS_151			1 = Strongly agree		QOL_QS_15

	Field Name	Data Type	Units	Values	Pre-Filled Values	Include Field OID
				2 = Slightly agree<br>3 = Neither agree nor disagree<br>4 = Slightly disagree<br>5 = Strongly disagree		
52	QOL_QS_161			1 = Strongly agree<br>2 = Slightly agree<br>3 = Neither agree nor disagree<br>4 = Slightly disagree<br>5 = Strongly disagree		QOL_QS_16
53	QOL_QS_171			1 = Strongly agree<br>2 = Slightly agree<br>3 = Neither agree nor disagree<br>4 = Slightly disagree		QOL_QS_17

	Field Name	Data Type	Units	Values	Pre-Filled Values	Include Field OID
				5 = Strongly disagree		
54	QOL_QS_181			1 = Strongly agree<br>2 = Slightly agree<br>3 = Neither agree nor disagree<br>4 = Slightly disagree<br>5 = Strongly disagree		QOL_QS_18
55	QOLH4	1				QOLH4
56	QOL_QS_191			1 = Strongly agree<br>2 = Slightly agree<br>3 = Neither agree nor disagree<br>4 = Slightly disagree<br>5 = Strongly disagree		QOL_QS_19

Production Germany 14. Jan. 2020: Primary matrix
Folder: Final evaluation TP1
Form: Final evaluation TP 1
Generated On: 30 Sep 2020 12:31:31

	Field Name	Data Type	Units	Values	Pre-Filled Values	Include Field OID
57	QOL_QS_201			1 = Strongly agree<br>2 = Slightly agree<br>3 = Neither agree nor disagree<br>4 = Slightly disagree<br>5 = Strongly disagree		QOL_QS_20
58	QOL_QS_211			1 = Strongly agree<br>2 = Slightly agree<br>3 = Neither agree nor disagree<br>4 = Slightly disagree<br>5 = Strongly disagree		QOL_QS_21
59	QOL_QS_221			1 = Strongly agree<br>2 = Slightly agree<br>3 = Neither agree nor disagree		QOL_QS_22

	Field Name	Data Type	Units	Values	Pre-Filled Values	Include Field OID
				4 = Slightly disagree<br>5 = Strongly disagree		
60	QOL_QS_23	1		1 = Strongly agree<br>2 = Slightly agree<br>3 = Neither agree nor disagree<br>4 = Slightly disagree<br>5 = Strongly disagree		QOL_QS_23
61	Feeling of security	1		1 = Very poor<br>2 = Poor<br>3 = Neither poor nor good<br>4 = Good<br>5 = Very good		SECURITY
62	Cutting difficulty	1		1 = Very difficult<br>2 = Difficult		CUTTING_DIFF

	Field Name	Data Type	Units	Values	Pre-Filled Values	Include Field OID
				3 = Neither difficult nor easy<br>4 = Easy<br>5 = Very easy		
63	Application of product	1		1 = Very difficult<br>2 = Difficult<br>3 = Neither difficult nor easy<br>4 = Easy<br>5 = Very easy		APPLICATION
64	Removal of baseplate	1		1 = Very difficult<br>2 = Difficult<br>3 = Neither difficult nor easy<br>4 = Easy<br>5 = Very easy		REMOVAL

Production Germany 14. Jan. 2020: Primary matrix
Folder: Product change TP2
Form: Type of change
Generated On: 30 Sep 2020 12:31:31

Product sequence number ______ (1)

What type of change do you want to register?

Bag change ☐ (2)
Baseplate change ☐

______ (3)

Production Germany 14. Jan. 2020: Primary matrix
Folder: Product change TP2
Form: Type of change
Generated On: 30 Sep 2020 12:31:31

	Field Name	Data Type	Units	Values	Pre-Filled Values	Include Field OID
1	Product sequence number	3				PRODSEQUENCE
2	Type of change	$3		BAG = Bag change<br>BP = Baseplate change		CNG_TYPE
3	HIDDEN	$100		{ "Change_type": { "BAG": "CHANGETIME_BP", "BP": "CHANGETIME_BP" } }	1: { "Change_type": { "BAG": "CHANGETIME_BP", "BP": "CHANGETIME_BP" } }	HIDDEN

Production Germany 14. Jan. 2020: Primary matrix
Folder: Product change TP2
Form: Time for performing change of bag
Generated On: 30 Sep 2020 12:31:31

Product sequence number ________________ (1)

Are you going to change now or have you changed previously without using the app? (2)

- ☐ I am going to change now
- ☐ I have changed previously without using the app (e.g. during the night)

---

(3)
{ "CNGnowprev": { "N": "BAGCNG", "P": "BAGCNGPREV" } }

Production Germany 14. Jan. 2020: Primary matrix
Folder: Product change TP2
Form: Time for performing change of bag
Generated On: 30 Sep 2020 12:31:31

	Field Name	Data Type	Units	Values	Pre-Filled Values	Include Field OID
1	Product sequence number	3				PRODSEQUENCE
2	Time for performing chance of bag	$1		N = I am going to change now P = I have changed previously without using the app (e.g. during the night)		CHANGETIME_BAG
3	HIDDEN	$100		{ "CNGnowprev": { "N": "BAGCNG", "P": "BAGCNGPREV" } }		HIDDEN

Production Germany 14. Jan. 2020: Primary matrix
Folder: Product change TP2
Form: Bag change previously
Generated On: 30 Sep 2020 12:31:31

Product sequence number ____________ (1)

When did you change the bag (approximately)? ____________ (2)

Production Germany 14. Jan. 2020: Primary matrix
Folder: Product change TP2
Form: Bag change previously
Generated On: 30 Sep 2020 12:31:31

	Field Name	Data Type	Units	Values	Pre-Filled Values	Include Field OID
1	Product sequence number	3				PRODSEQUENCE
2	When did you change the bag	dd MMM yyyy HH:nn				CNGPREVDTC

Production Germany 14. Jan. 2020: Primary matrix
Folder: Product change TP2
Form: Bag change
Generated On: 30 Sep 2020 12:31:31

Product sequence number	______	1
Photo of the baseplate on the skin	______	2
Baseplate on skin photo file name	______	3
Baseplate on skin photo date and time	______	4

Production Germany 14. Jan. 2020: Primary matrix
Folder: Product change TP2
Form: Bag change
Generated On: 30 Sep 2020 12:31:31

	Field Name	Data Type	Units	Values	Pre-Filled Values	Include Field OID
1	Product sequence number	3				PRODSEQUENCE
2	Photo of the baseplate on the skin	1				PHOTO_BPONSKIN
3	Photo file name	$100				BPONSKIN_PHOTO
4	Baseplate on skin photo	dd MMM yyyy HH:nn				PHT03DTC

Production Germany 14. Jan. 2020: Primary matrix
Folder: Product change TP2
Form: Time for performing change of BP
Generated On: 30 Sep 2020 12:31:31

Product sequence number ______________ (1)

Are you going to change now or have you changed previously?

- I am going to change now ☐ (2)
- I have changed previously without using the app (e.g. during the night) ☐

______________ (3)

Production Germany 14. Jan. 2020: Primary matrix
Folder: Product change TP2
Form: Time for performing change of BP
Generated On: 30 Sep 2020 12:31:31

	Field Name	Data Type	Units	Values	Pre-Filled Values	Include Field OID
1	Product sequence number	3				PRODSEQUENCE
2	Time for performing change of baseplate	$1		N = I am going to change now<br>P = I have changed previously without using the app (e.g. during the night)		CHANGETIME_BP
3	HIDDEN	$100		{ "CNGnowprev": { "N": "BPCNG", "P": "BPCNGPREV" } }	1: { "CNGnowprev": { "N": "BPCNG", "P": "BPCNGPREV" } }	HIDDEN

Production Germany 14. Jan. 2020: Primary matrix
Folder: Product change TP2
Form: Baseplate change previously
Generated On: 30 Sep 2020 12:31:31

Product sequence number ____________________ (1)

When did you change the product (approximately)? ____________________ (2)

Production Germany 14. Jan. 2020: Primary matrix
Folder: Product change TP2
Form: Baseplate change previously
Generated On: 30 Sep 2020 12:31:31

	Field Name	Data Type	Units	Values	Pre-Filled Values	Include Field OID
1	Product sequence number	3				PRODSEQU ENCE
2	Product change previously	dd MMM yyyy HH:nn				CNGPREVD TC

Production Germany 14. Jan. 2020: Primary matrix
Folder: Product change TP2
Form: Baseplate change TP 1
Generated On: 30 Sep 2020 12:31:31

Product sequence number ________ (1)

Photo of the stoma ________ (2)

Stoma photo file name ________ (3)

Stoma photo date and time ________ (4)

Photo of the product ________ (5)

Product photo file name ________ (6)

Product photo date and time ________ (7)

Do you experience any bleeding from the skin around your stoma right now when changing your product? (8)
- Not experiencing ☐
- Experiencing ☐

Once you have cleaned and dried the skin, do you still experience any weeping or moisture on the skin around your stoma right now when changing your product? (9)
- Not experiencing ☐
- Experiencing ☐

Are you experiencing any ulcers or sores around your stoma right now when changing your product? (10)
- Not experiencing ☐
- Experiencing ☐

How much of the time have you experienced any itching around your stoma since you last changed your product? (11)
- None of the time ☐
- A little of the time ☐
- Some of the time ☐
- A lot of the time ☐
- All of the time ☐

Please rate on a scale from 0-10 how itchy the skin around your stoma has been at its worst since you last changed your product

12

- ☐ 0 No itch
- ☐ 1 Very mild itch
- ☐ 2
- ☐ 3
- ☐ 4
- ☐ 5
- ☐ 6
- ☐ 7
- ☐ 8
- ☐ 9
- ☐ 10 Worst possible peristomal skin itch

How much of the time have you experienced any pain around your stoma since you last changed your product?

13

- ☐ None of the time
- ☐ A little of the time
- ☐ Some of the time
- ☐ A lot of the time
- ☐ All of the time

Please rate on a scale from 0-10 how painful the skin around your stoma has been at its worst since you last changed your product

14

- ☐ 0 No pain
- ☐ 1 Very mild pain
- ☐ 2
- ☐ 3
- ☐ 4
- ☐ 5
- ☐ 6
- ☐ 7
- ☐ 8
- ☐ 9
- ☐ 10 Worst possible peristomal skin pain

How much of the time have you experienced any burning feelings from the skin around your stoma since you last changed your product?

- None of the time ▢ 15
- A little of the time ▢
- Some of the time ▢
- A lot of the time ▢
- All of the time ▢

Please rate on a scale from 0-10 any burning feelings from the skin around your stoma at its worst since you last changed your product

- 0 No burning ▢ 16
- 1 Very mild burning ▢
- 2 ▢
- 3 ▢
- 4 ▢
- 5 ▢
- 6 ▢
- 7 ▢
- 8 ▢
- 9 ▢
- 10 Worst possible peristomal skin burning ▢

How would you rate on a scale of 0-10 your overall physical discomfort from the skin around your stoma since you last changed your product?

- 0 No discomfort ▢ 17
- 1 ▢
- 2 ▢
- 3 ▢
- 4 ▢
- 5 ▢
- 6 ▢
- 7 ▢
- 8 ▢
- 9 ▢
- 10 A lot of discomfort ▢

On a scale of 0-10, how much have skin complications around your stoma caused you difficulty in your overall ability to move around since you last changed your product? For example, bending or walking 18

- No skin complications ▢
- 0 No difficulty ▢
- 1 ▢
- 2 ▢
- 3 ▢
- 4 ▢
- 5 ▢
- 6 ▢
- 7 ▢
- 8 ▢
- 9 ▢
- 10 A lot of difficulty ▢

On a scale of 0-10, how much have skin complications around your stoma caused you difficulty in your overall ability to stick the stoma bag adhesive to your skin since you last changed your product? 19

- No skin complications ▢
- 0 No difficulty ▢
- 1 ▢
- 2 ▢
- 3 ▢
- 4 ▢
- 5 ▢
- 6 ▢
- 7 ▢
- 8 ▢
- 9 ▢
- 10 A lot of difficulty ▢

What was the main reason for change of the baseplate? ____________________ 20

I followed my usual changing pattern ____________________ 21

I thought it would be nice with a clean product	______	22
In preparation of an activity (i.e. going out, doing sports, travelling)	______	23
The entire baseplate had become detached	______	24
The outer edge of the baseplate had become detached	______	25
The center of the baseplate had become detached	______	26
I was afraid the baseplate would become detached	______	27
The area around the stoma was itching	______	28
The area around the stoma was painful	______	29
There was leakage underneath and outside the baseplate	______	30
There was leakage underneath the baseplate (but not outside the baseplate)	______	31
The bag was full of air (ballooning)	______	32
There was a vacuum in the bag (pancaking)	______	33
Due to visit in the investigation	______	34
Other reason	______	35

If other reason for change is selected, please specify	____________	36
Did you experience leakage outside the baseplate (i.e. soiling the clothes)?	No ☐<br>Yes ☐	37
Did you experience the bag becoming overfilled with air during use (ballooning)?	Not at all ☐<br>A little ☐<br>Some ☐<br>Much ☐<br>Very much ☐	38
Which stoma accessories did you use?		
None	____________	40
Adhesive remover	____________	41
Paste	____________	42
Rings	____________	43
Ostomy tape	____________	44
Ostomy belt	____________	45
Hernia belt	____________	46
Stoma powder	____________	47

Production Germany 14. Jan. 2020: Primary matrix
Folder: Product change TP2
Form: Baseplate change TP 1
Generated On: 30 Sep 2020 12:31:31

Barrier lotion	______	48
Barrier cream	______	49
Barrier spray	______	50
Barrier wipes	______	51
Cleansing wipes/cleansing spray	______	52
Odour remover	______	53
Other accessories	______	54
If ‘Other’ please specify:	______	55

Production Germany 14. Jan. 2020: Primary matrix
Folder: Product change TP2
Form: Baseplate change TP 1
Generated On: 30 Sep 2020 12:31:31

	Field Name	Data Type	Units	Values	Pre-Filled Values	Include Field OID
1	Product sequence number	3				PRODSEQUENCE
2	Photo of the stoma	1				PHOTO_SKIN
3	Stoma photo name	$100				PHT0101
4	Stoma photo date and time	dd MMM yyyy HH:nn				PHT01DTC
5	Photo of the product	1				PHOTO_PRODUCT
6	Product photo name	$100				PROD_PHOTO
7	Product photo date and time	dd MMM yyyy HH:nn				PHT02DTC
8	Ostomy Skin Tool 1	1		0 = Not experiencing<br>1 = Experiencing		OST_1

Production Germany 14. Jan. 2020: Primary matrix
Folder: Product change TP2
Form: Baseplate change TP 1
Generated On: 30 Sep 2020 12:31:31

	Field Name	Data Type	Units	Values	Pre-Filled Values	Include Field OID
9	Ostomy Skin Tool 2	1		0 = Not experiencing<br>1 = Experiencing		OST_2
10	Ostomy Skin Tool 3	1		0 = Not experiencing<br>1 = Experiencing		OST_3
11	Ostomy Skin Tool 4	1		1 = None of the time<br>2 = A little of the time<br>3 = Some of the time<br>4 = A lot of the time<br>5 = All of the time		OST_4
12	Ostomy Skin Tool 5	2		0 = 0 No itch<br>1 = 1 Very mild itch<br>2 = 2<br>3 = 3<br>4 = 4<br>5 = 5<br>6 = 6<br>7 = 7		LONGSCALE_OST_5

	Field Name	Data Type	Units	Values	Pre-Filled Values	Include Field OID
				8 = 8<br>9 = 9<br>10 = 10 Worst possible peristomal skin itch		
13	Ostomy Skin Tool 6	1		1 = None of the time<br>2 = A little of the time<br>3 = Some of the time<br>4 = A lot of the time<br>5 = All of the time		OST_6
14	Ostomy Skin Tool 7	2		0 = 0 No pain<br>1 = 1 Very mild pain<br>2 = 2<br>3 = 3<br>4 = 4<br>5 = 5<br>6 = 6<br>7 = 7<br>8 = 8<br>9 = 9<br>10 = 10 Worst possible peristomal skin pain		LONGSCALE_OST_7

	Field Name	Data Type	Units	Values	Pre-Filled Values	Include Field OID
15	Ostomy Skin Tool 8	1		1 = None of the time<br>2 = A little of the time<br>3 = Some of the time<br>4 = A lot of the time<br>5 = All of the time		OST_8
16	Ostomy Skin Tool 9	2		0 = 0 No burning<br>1 = 1 Very mild burning<br>2 = 2<br>3 = 3<br>4 = 4<br>5 = 5<br>6 = 6<br>7 = 7<br>8 = 8<br>9 = 9<br>10 = 10 Worst possible peristomal skin burning		LONGSCALE _OST_9
17	Ostomy Skin Tool 10	2		0 = 0 No discomfort<br>1 = 1<br>2 = 2<br>3 = 3<br>4 = 4		LONGSCALE _OST_10

	Field Name	Data Type	Units	Values	Pre-Filled Values	Include Field OID
				5 = 5<br>6 = 6<br>7 = 7<br>8 = 8<br>9 = 9<br>10 = 10 A lot of discomfort		
18	Ostomy Skin Tool 11	2		99 = No skin complications<br>0 = 0 No difficulty<br>1 = 1<br>2 = 2<br>3 = 3<br>4 = 4<br>5 = 5<br>6 = 6<br>7 = 7<br>8 = 8<br>9 = 9<br>10 = 10 A lot of difficulty		LONGSCALE_OST_11
19	Ostomy Skin Tool 12	2		99 = No skin complications<br>0 = 0 No difficulty<br>1 = 1<br>2 = 2		LONGSCALE_OST_12

	Field Name	Data Type	Units	Values	Pre-Filled Values	Include Field OID
				3 = 3<br>4 = 4<br>5 = 5<br>6 = 6<br>7 = 7<br>8 = 8<br>9 = 9<br>10 = 10 A lot of difficulty		
20	MULTI_CNG_HEADER	1				MULTI_CNG_HEADER
21	Reason for change no. 1	1				MULTI_CNG_1
22	Reason for change no. 2	1				MULTI_CNG_2
23	Reason for change no. 3	1				MULTI_CNG_3
24	Reason for change no. 4	1				MULTI_CNG_4

Production Germany 14. Jan. 2020: Primary matrix
Folder: Product change TP2
Form: Baseplate change TP 1
Generated On: 30 Sep 2020 12:31:31

	Field Name	Data Type	Units	Values	Pre-Filled Values	Include Field OID
25	Reason for change no. 5	1				MULTI_CNG _5
26	Reason for change no. 6	1				MULTI_CNG _6
27	Reason for change no. 7	1				MULTI_CNG _7
28	Reason for change no. 8	1				MULTI_CNG _8
29	Reason for change no. 9	1				MULTI_CNG _9
30	Reason for change no. 10	1				MULTI_CNG _10
31	Reason for change no. 11	1				MULTI_CNG _11
32	Reason for change no. 12	1				MULTI_CNG _12

Production Germany 14. Jan. 2020: Primary matrix
Folder: Product change TP2
Form: Baseplate change TP 1
Generated On: 30 Sep 2020 12:31:31

	Field Name	Data Type	Units	Values	Pre-Filled Values	Include Field OID
33	Reason for change no. 13	1				MULTI_CNG_13
34	Reason for change no. 14	1				MULTI_CNG_14
35	Reason for change no. 15	1				MULTI_CNG_15
36	Description of other reason for change	$100				MULTI_CNG_OTHER
37	Leakage outside	1		0 = No<br>1 = Yes		LEAKAGE_OUTSIDE
38	Degree of ballooning	1		1 = Not at all<br>2 = A little<br>3 = Some<br>4 = Much<br>5 = Very much		BALLOONING_DEGREE
40	Accessories 1	1				MULTI_ACC_1

	Field Name	Data Type	Units	Values	Pre-Filled Values	Include	Field OID
41	Accessories 2	1					MULTI_ACC_2
42	Accessories 3	1					MULTI_ACC_3
43	Accessories 4	1					MULTI_ACC_4
44	Accessories 5	1					MULTI_ACC_5
45	Accessories 6	1					MULTI_ACC_6
46	Accessories 7	1					MULTI_ACC_7
47	Accessories 8	1					MULTI_ACC_8
48	Accessories 9	1					MULTI_ACC_9
49	Accessories 10	1					MULTI_ACC_10
50	Accessories 11	1					MULTI_ACC_11

	Field Name	Data Type	Units	Values	Pre-Filled Values	Include Field OID
51	Accessories 12	1				MULTI_ACC_12
52	Accessories 13	1				MULTI_ACC_13
53	Accessories 14	1				MULTI_ACC_14
54	Accessories 15	1				MULTI_ACC_15
55	Accessories other	$50				MULTI_ACC_OTHER

Do you have any skin complications around your stoma today?

- No ☐ (1)
- Yes ☐

If yes, how would you describe the skin complications around your stoma today?

- No skin complications ☐ (2)
- Very mild ☐
- Mild ☐
- Moderate ☐
- Severe ☐
- Very severe ☐

How often have you experienced itchy skin around your stoma in the past 14 days?

- None of the time ☐ (3)
- A little of the time ☐
- Some of the time ☐
- A lot of the time ☐
- All of the time ☐

How often have you experienced any pain in the skin around the stoma in the past 14 days?

- None of the time ☐ (4)
- A little of the time ☐
- Some of the time ☐
- A lot of the time ☐
- All of the time ☐

How often have you experienced burning feelings in the skin around your stoma in the past 14 days?

- None of the time ☐ (5)
- A little of the time ☐
- Some of the time ☐
- A lot of the time ☐
- All of the time ☐

Compared to the beginning of this test period (Test Period 2) how have any skin complications around your stoma changed? **6**

- Very much improved ☐
- Much improved ☐
- A little improved ☐
- No change ☐
- A little worse ☐
- Much worse ☐
- Very much worse ☐

Did your stoma behave differently from normal since your last visit/call? **7**

- No ☐
- Yes ☐

If yes, please describe ________________ **8**

Emotional impact. In the last seven days, due to leakage or worry about leakage...
(please tick the box to continue) ________________ **9**

I felt panic **10**

- All of the time ☐
- Often ☐
- Sometimes ☐
- Rarely or never ☐

I felt stressed out **11**

- All of the time ☐
- Often ☐
- Sometimes ☐
- Rarely or never ☐

I felt more afraid about leaks in the future **12**

- All of the time ☐
- Often ☐

Sometimes ☐
Rarely or never ☐

I felt worry

All of the time ☐ 13
Often ☐
Sometimes ☐
Rarely or never ☐

I felt frustrated

All of the time ☐ 14
Often ☐
Sometimes ☐
Rarely or never ☐

I felt embarrassed

All of the time ☐ 15
Often ☐
Sometimes ☐
Rarely or never ☐

I felt worried that I might leak

All of the time ☐ 16
Often ☐
Sometimes ☐
Rarely or never ☐

I couldn't sleep

All of the time ☐ 17
Often ☐
Sometimes ☐
Rarely or never ☐

I kept waking up at night to check my stoma — 18

- All of the time ☐
- Often ☐
- Sometimes ☐
- Rarely or never ☐

I kept checking my ostomy bag to see if I have leaked — 19

- All of the time ☐
- Often ☐
- Sometimes ☐
- Rarely or never ☐

Usual and social activities.
In the last seven days due to leakage or worry about leakage... — 20

I decided to stay at home — 21

- All of the time ☐
- Often ☐
- Sometimes ☐
- Rarely or never ☐
- Not applicable ☐

I couldn't do light activities — 22

- All of the time ☐
- Often ☐
- Sometimes ☐
- Rarely or never ☐
- Not applicable ☐

I changed my plans — 23

- All of the time ☐
- Often ☐
- Sometimes ☐

Rarely or never ▢
Not applicable ▢

I was unable to go out and meet family and friends — 24
All of the time ▢
Often ▢
Sometimes ▢
Rarely or never ▢
Not applicable ▢

I avoided close physical contact with family and friends — 25
All of the time ▢
Often ▢
Sometimes ▢
Rarely or never ▢
Not applicable ▢

I did not want to see people — 26
All of the time ▢
Often ▢
Sometimes ▢
Rarely or never ▢
Not applicable ▢

I avoided people — 27
All of the time ▢
Often ▢
Sometimes ▢
Rarely or never ▢
Not applicable ▢

I tried to avoid meeting new people — 28
All of the time ▢
Often ▢

Sometimes ☐
Rarely or never ☐
Not applicable ☐

Coping and in control.
In the last 7 days, due to leakage or worry about leakage... 29

I felt in control 30

All of the time ☐
Often ☐
Sometimes ☐
Rarely or never ☐

I was able to cope 31

All of the time ☐
Often ☐
Sometimes ☐
Rarely or never ☐

I felt calm 32

All of the time ☐
Often ☐
Sometimes ☐
Rarely or never ☐

I saw my friends as I usually do 33

All of the time ☐
Often ☐
Sometimes ☐
Rarely or never ☐

Discreetness.
In the last seven days... 34

It was difficult to hide the stoma appliance under clothing 35

- Strongly agree ☐
- Slightly agree ☐
- Neither agree nor disagree ☐
- Slightly disagree ☐
- Strongly disagree ☐

I was self-conscious about the appearance of the stoma appliance 36

- Strongly agree ☐
- Slightly agree ☐
- Neither agree nor disagree ☐
- Slightly disagree ☐
- Strongly disagree ☐

The stoma appliance limited the choice of clothes that I could wear 37

- Strongly agree ☐
- Slightly agree ☐
- Neither agree nor disagree ☐
- Slightly disagree ☐
- Strongly disagree ☐

The stoma appliance was obvious to other people 38

- Strongly agree ☐
- Slightly agree ☐
- Neither agree nor disagree ☐
- Slightly disagree ☐
- Strongly disagree ☐

The color of the stoma appliance was discreet 39

- Strongly agree ☐
- Slightly agree ☐

Neither agree nor disagree ☐
Slightly disagree ☐
Strongly disagree ☐

It was difficult to hide the stoma appliance because of ballooning

Strongly agree ☐ 40
Slightly agree ☐
Neither agree nor disagree ☐
Slightly disagree ☐
Strongly disagree ☐

Comfort.
In the last seven days... 41

The stoma appliance was comfortable to wear

Strongly agree ☐ 42
Slightly agree ☐
Neither agree nor disagree ☐
Slightly disagree ☐
Strongly disagree ☐

I was not concerned about skin irritation under the stoma appliance (for example feelings of burning, itching, pinching or pain)

Strongly agree ☐ 43
Slightly agree ☐
Neither agree nor disagree ☐
Slightly disagree ☐
Strongly disagree ☐

It was uncomfortable to remove the stoma appliance from my body

Strongly agree ☐ 44
Slightly agree ☐
Neither agree nor disagree ☐
Slightly disagree ☐

Strongly disagree ▢

I often forgot that I was wearing the stoma appliance (45)

- Strongly agree ▢
- Slightly agree ▢
- Neither agree nor disagree ▢
- Slightly disagree ▢
- Strongly disagree ▢

The stoma appliance was comfortable as it fitted well to my body movements (46)

- Strongly agree ▢
- Slightly agree ▢
- Neither agree nor disagree ▢
- Slightly disagree ▢
- Strongly disagree ▢

The stoma appliance disrupted my sleep during the night (47)

- Strongly agree ▢
- Slightly agree ▢
- Neither agree nor disagree ▢
- Slightly disagree ▢
- Strongly disagree ▢

Confidence.
In the last seven days... (48)

____

I was confident that the stoma appliance would not leak (49)

- Strongly agree ▢
- Slightly agree ▢
- Neither agree nor disagree ▢
- Slightly disagree ▢
- Strongly disagree ▢

I worried that the stoma appliance would become loose from my body

50

- Strongly agree
- Slightly agree
- Neither agree nor disagree
- Slightly disagree
- Strongly disagree

I felt confident that I could spend the night away from home despite wearing the stoma appliance

51

- Strongly agree
- Slightly agree
- Neither agree nor disagree
- Slightly disagree
- Strongly disagree

I was confident the stoma appliance would not cause any problems for me

52

- Strongly agree
- Slightly agree
- Neither agree nor disagree
- Slightly disagree
- Strongly disagree

I felt confident to take part in physical activities (for example, sports) whilst wearing the stoma appliance

53

- Strongly agree
- Slightly agree
- Neither agree nor disagree
- Slightly disagree
- Strongly disagree

I worried that the stoma appliance would make a rustling noise

54

- Strongly agree
- Slightly agree
- Neither agree nor disagree
- Slightly disagree
- Strongly disagree

Social life and relationships.
In the last seven days... 55

I worried that my family and friends felt awkward around me because of the stoma appliance 56

- Strongly agree ☐
- Slightly agree ☐
- Neither agree nor disagree ☐
- Slightly disagree ☐
- Strongly disagree ☐

I felt my social life had been restricted because of the stoma appliance 57

- Strongly agree ☐
- Slightly agree ☐
- Neither agree nor disagree ☐
- Slightly disagree ☐
- Strongly disagree ☐

I avoided close physical contact with family and friends because of the stoma appliance 58

- Strongly agree ☐
- Slightly agree ☐
- Neither agree nor disagree ☐
- Slightly disagree ☐
- Strongly disagree ☐

I worried about whether I could have a relationship because of my stoma appliance 59

- Strongly agree ☐
- Slightly agree ☐
- Neither agree nor disagree ☐
- Slightly disagree ☐
- Strongly disagree ☐

I worried about whether the stoma appliance would affect my sex life 60

- Strongly agree ☐
- Slightly agree ☐

	Neither agree nor disagree ▢<br>Slightly disagree ▢<br>Strongly disagree ▢
How was the feeling of security while wearing the product?	Very poor ▢ (61)<br>Poor ▢<br>Neither poor nor good ▢<br>Good ▢<br>Very good ▢
How did you find cutting the baseplate?	Very difficult ▢ (62)<br>Difficult ▢<br>Neither difficult nor easy ▢<br>Easy ▢<br>Very easy ▢
How did you find applying the baseplate?	Very difficult ▢ (63)<br>Difficult ▢<br>Neither difficult nor easy ▢<br>Easy ▢<br>Very easy ▢
How did you find removing the baseplate?	Very difficult ▢ (64)<br>Difficult ▢<br>Neither difficult nor easy ▢<br>Easy ▢<br>Very easy ▢
Which product do you prefer?	(65)

The product from the first test period ▢
The product from the second test period ▢

Why do you prefer this product over the other? (you may tick more than one box)

Less leakage	______	67
Better feeling of security	______	68
Better comfort	______	69
Better discretion	______	70
Less pancaking	______	71
Less ballooning	______	72
Better skin condition	______	73
Better ability to fit and follow the body	______	74
Other reason	______	75

Which product do you prefer? (76)

- The product from the first test period ▢
- The product from the second test period ▢
- Your own product ▢

Production Germany 14. Jan. 2020: Primary matrix
Folder: Final evaluation TP2
Form: Final evaluation TP 2
Generated On: 30 Sep 2020 12:31:31

Why do you prefer this product over the other? (you may tick more than one box)

Less leakage	78
Better feeling of security	79
Better comfort	80
Better discretion	81
Less pancaking	82
Less ballooning	83
Better skin condition	84
Better ability to fit and follow the body	85
Other reason	86

	Field Name	Data Type	Units	Values	Pre-Filled Values	Include Field OID
1	Complications today	1		0 = No<br>1 = Yes		COMP_TODAY
2	Severity of complications	1		0 = No skin complications<br>1 = Very mild<br>2 = Mild<br>3 = Moderate<br>4 = Severe<br>5 = Very severe		COMP_IF_YES
3	Itching in 14 days	1		1 = None of the time<br>2 = A little of the time<br>3 = Some of the time<br>4 = A lot of the time<br>5 = All of the time		ITCHING_14DAYS
4	Pain in 14 days	1		1 = None of the time<br>2 = A little of the time<br>3 = Some of the time<br>4 = A lot of the time		PAIN_14DAYS

	Field Name	Data Type	Units	Values	Pre-Filled Values	Include Field OID
				5 = All of the time		
5	Burning in 14 days	1		1 = None of the time<br>2 = A little of the time<br>3 = Some of the time<br>4 = A lot of the time<br>5 = All of the time		BURNING_14DAYS
6	Own comparison of skin	1		1 = Very much improved<br>2 = Much improved<br>3 = A little improved<br>4 = No change<br>5 = A little worse<br>6 = Much worse<br>7 = Very much worse		COMPARE_OWN
7	Stoma behaviour	1		0 = No<br>1 = Yes		STOMABEHAVE_YN

	Field Name	Data Type	Units	Values	Pre-Filled Values	Include Field OID
8	Different stoma behaviour	$200				BEHAVE_IF_YES
9	LEAKAGE_HEADER	1				LEAKAGE_HEADER
10	LEAKAGE_QS_1	1		0 = All of the time<br>1 = Often<br>2 = Sometimes<br>3 = Rarely or never		LEAKAGE_QS_1
11	LEAKAGE_QS_2	1		0 = All of the time<br>1 = Often<br>2 = Sometimes<br>3 = Rarely or never		LEAKAGE_QS_2
12	LEAKAGE_QS_3	1		0 = All of the time<br>1 = Often<br>2 = Sometimes<br>3 = Rarely or never		LEAKAGE_QS_3
13	LEAKAGE_QS_4	1		0 = All of the time		LEAKAGE_QS_4

	Field Name	Data Type	Units	Values	Pre-Filled Values	Include Field OID
				1 = Often<br>2 = Sometimes<br>3 = Rarely or never		
14	LEAKAGE_Q1 S_5			0 = All of the time<br>1 = Often<br>2 = Sometimes<br>3 = Rarely or never		LEAKAGE_Q S_5
15	LEAKAGE_Q1 S_6			0 = All of the time<br>1 = Often<br>2 = Sometimes<br>3 = Rarely or never		LEAKAGE_Q S_6
16	LEAKAGE_Q1 S_7			0 = All of the time<br>1 = Often<br>2 = Sometimes<br>3 = Rarely or never		LEAKAGE_Q S_7
17	LEAKAGE_Q1 S_8			0 = All of the time<br>1 = Often		LEAKAGE_Q S_8

	Field Name	Data Type	Units	Values	Pre-Filled Values	Include Field OID
				2 = Sometimes 3 = Rarely or never		
18	LEAKAGE_Q1S_9			0 = All of the time 1 = Often 2 = Sometimes 3 = Rarely or never		LEAKAGE_QS_9
19	LEAKAGE_Q1S_10			0 = All of the time 1 = Often 2 = Sometimes 3 = Rarely or never		LEAKAGE_QS_10
20	LEAKAGE_H1EADER2					LEAKAGE_HEADER2
21	LEAKAGE_Q1S_11			0 = All of the time 1 = Often 2 = Sometimes 3 = Rarely or never 9 = Not applicable		LEAKAGE_QS_11

	Field Name	Data Type	Units	Values	Pre-Filled Values	Include Field OID
22	LEAKAGE_Q1S_12			0 = All of the time<br>1 = Often<br>2 = Sometimes<br>3 = Rarely or never<br>9 = Not applicable		LEAKAGE_QS_12
23	LEAKAGE_Q1S_13			0 = All of the time<br>1 = Often<br>2 = Sometimes<br>3 = Rarely or never<br>9 = Not applicable		LEAKAGE_QS_13
24	LEAKAGE_Q1S_14			0 = All of the time<br>1 = Often<br>2 = Sometimes<br>3 = Rarely or never<br>9 = Not applicable		LEAKAGE_QS_14
25	LEAKAGE_Q1S_15			0 = All of the time<br>1 = Often<br>2 = Sometimes		LEAKAGE_QS_15

	Field Name	Data Type	Units	Values	Pre-Filled Values	Include Field OID
				3 = Rarely or never 9 = Not applicable		
26	LEAKAGE_Q1 S_16			0 = All of the time 1 = Often 2 = Sometimes 3 = Rarely or never 9 = Not applicable		LEAKAGE_Q S_16
27	LEAKAGE_Q1 S_17			0 = All of the time 1 = Often 2 = Sometimes 3 = Rarely or never 9 = Not applicable		LEAKAGE_Q S_17
28	LEAKAGE_Q1 S_18			0 = All of the time 1 = Often 2 = Sometimes 3 = Rarely or never 9 = Not applicable		LEAKAGE_Q S_18

Production Germany 14. Jan. 2020: Primary matrix
Folder: Final evaluation TP2
Form: Final evaluation TP 2
Generated On: 30 Sep 2020 12:31:31

	Field Name	Data Type	Units	Values	Pre-Filled Values	Include Field OID
29	LEAKAGE_H1 EADER3					LEAKAGE_H EADER3
30	LEAKAGE_Q1 S_19			0 = All of the time 1 = Often 2 = Sometimes 3 = Rarely or never		LEAKAGE_Q S_19
31	LEAKAGE_Q1 S_20			0 = All of the time 1 = Often 2 = Sometimes 3 = Rarely or never		LEAKAGE_Q S_20
32	LEAKAGE_Q1 S_21			0 = All of the time 1 = Often 2 = Sometimes 3 = Rarely or never		LEAKAGE_Q S_21
33	LEAKAGE_Q1 S_22			0 = All of the time 1 = Often 2 = Sometimes 3 = Rarely or never		LEAKAGE_Q S_22

	Field Name	Data Type	Units	Values	Pre-Filled Values	Include Field OID
34	QOLH1	1				QOLH1
35	QOL_QS_1	1		1 = Strongly agree<br>2 = Slightly agree<br>3 = Neither agree nor disagree<br>4 = Slightly disagree<br>5 = Strongly disagree		QOL_QS_1
36	QOL_QS_2	1		1 = Strongly agree<br>2 = Slightly agree<br>3 = Neither agree nor disagree<br>4 = Slightly disagree<br>5 = Strongly disagree		QOL_QS_2
37	QOL_QS_3	1		1 = Strongly agree		QOL_QS_3

	Field Name	Data Type	Units	Values	Pre-Filled Values	Include Field OID
				2 = Slightly agree<br>3 = Neither agree nor disagree<br>4 = Slightly disagree<br>5 = Strongly disagree		
38	QOL_QS_4	1		1 = Strongly agree<br>2 = Slightly agree<br>3 = Neither agree nor disagree<br>4 = Slightly disagree<br>5 = Strongly disagree		QOL_QS_4
39	QOL_QS_5	1		1 = Strongly agree<br>2 = Slightly agree<br>3 = Neither agree nor disagree<br>4 = Slightly disagree		QOL_QS_5

	Field Name	Data Type	Units	Values	Pre-Filled Values	Include Field OID
				5 = Strongly disagree		
40	QOL_QS_6	1		1 = Strongly agree<br>2 = Slightly agree<br>3 = Neither agree nor disagree<br>4 = Slightly disagree<br>5 = Strongly disagree		QOL_QS_6
41	QOLH2	1				QOLH2
42	QOL_QS_7	1		1 = Strongly agree<br>2 = Slightly agree<br>3 = Neither agree nor disagree<br>4 = Slightly disagree<br>5 = Strongly disagree		QOL_QS_7

	Field Name	Data Type	Units	Values	Pre-Filled Values	Include Field OID
43	QOL_QS_8	1		1 = Strongly agree<br>2 = Slightly agree<br>3 = Neither agree nor disagree<br>4 = Slightly disagree<br>5 = Strongly disagree		QOL_QS_8
44	QOL_QS_9	1		1 = Strongly agree<br>2 = Slightly agree<br>3 = Neither agree nor disagree<br>4 = Slightly disagree<br>5 = Strongly disagree		QOL_QS_9
45	QOL_QS_10	1		1 = Strongly agree<br>2 = Slightly agree<br>3 = Neither agree nor disagree		QOL_QS_10

	Field Name	Data Type	Units	Values	Pre-Filled Values	Include Field OID
				4 = Slightly disagree<br>5 = Strongly disagree		
46	QOL_QS_111			1 = Strongly agree<br>2 = Slightly agree<br>3 = Neither agree nor disagree<br>4 = Slightly disagree<br>5 = Strongly disagree		QOL_QS_11
47	QOL_QS_121			1 = Strongly agree<br>2 = Slightly agree<br>3 = Neither agree nor disagree<br>4 = Slightly disagree<br>5 = Strongly disagree		QOL_QS_12

Production Germany 14. Jan. 2020: Primary matrix
Folder: Final evaluation TP2
Form: Final evaluation TP 2
Generated On: 30 Sep 2020 12:31:31

	Field Name	Data Type	Units	Values	Pre-Filled Values	Include Field OID
48	QOLH3	1				QOLH3
49	QOL_QS_131			1 = Strongly agree<br>2 = Slightly agree<br>3 = Neither agree nor disagree<br>4 = Slightly disagree<br>5 = Strongly disagree		QOL_QS_13
50	QOL_QS_141			1 = Strongly agree<br>2 = Slightly agree<br>3 = Neither agree nor disagree<br>4 = Slightly disagree<br>5 = Strongly disagree		QOL_QS_14
51	QOL_QS_151			1 = Strongly agree		QOL_QS_15

	Field Name	Data Type	Units	Values	Pre-Filled Values	Include Field OID
				2 = Slightly agree<br>3 = Neither agree nor disagree<br>4 = Slightly disagree<br>5 = Strongly disagree		
52	QOL_QS_161			1 = Strongly agree<br>2 = Slightly agree<br>3 = Neither agree nor disagree<br>4 = Slightly disagree<br>5 = Strongly disagree		QOL_QS_16
53	QOL_QS_171			1 = Strongly agree<br>2 = Slightly agree<br>3 = Neither agree nor disagree<br>4 = Slightly disagree		QOL_QS_17

	Field Name	Data Type	Units	Values	Pre-Filled Values	Include Field OID
				5 = Strongly disagree		
54	QOL_QS_181			1 = Strongly agree<br>2 = Slightly agree<br>3 = Neither agree nor disagree<br>4 = Slightly disagree<br>5 = Strongly disagree		QOL_QS_18
55	QOLH4	1				QOLH4
56	QOL_QS_191			1 = Strongly agree<br>2 = Slightly agree<br>3 = Neither agree nor disagree<br>4 = Slightly disagree<br>5 = Strongly disagree		QOL_QS_19

	Field Name	Data Type	Units	Values	Pre-Filled Values	Include Field OID
57	QOL_QS_201			1 = Strongly agree<br>2 = Slightly agree<br>3 = Neither agree nor disagree<br>4 = Slightly disagree<br>5 = Strongly disagree		QOL_QS_20
58	QOL_QS_211			1 = Strongly agree<br>2 = Slightly agree<br>3 = Neither agree nor disagree<br>4 = Slightly disagree<br>5 = Strongly disagree		QOL_QS_21
59	QOL_QS_221			1 = Strongly agree<br>2 = Slightly agree<br>3 = Neither agree nor disagree		QOL_QS_22

	Field Name	Data Type	Units	Values	Pre-Filled Values	Include Field OID
				4 = Slightly disagree<br>5 = Strongly disagree		
60	QOL_QS_231			1 = Strongly agree<br>2 = Slightly agree<br>3 = Neither agree nor disagree<br>4 = Slightly disagree<br>5 = Strongly disagree		QOL_QS_23
61	Feeling of security	1		1 = Very poor<br>2 = Poor<br>3 = Neither poor nor good<br>4 = Good<br>5 = Very good		SECURITY
62	Cutting difficulty	1		1 = Very difficult<br>2 = Difficult		CUTTING_DIFF

	Field Name	Data Type	Units	Values	Pre-Filled Values	Include Field OID
				3 = Neither difficult nor easy<br>4 = Easy<br>5 = Very easy		
63	Application of product	1		1 = Very difficult<br>2 = Difficult<br>3 = Neither difficult nor easy<br>4 = Easy<br>5 = Very easy		APPLICATIO N
64	Removal of baseplate	1		1 = Very difficult<br>2 = Difficult<br>3 = Neither difficult nor easy<br>4 = Easy<br>5 = Very easy		REMOVAL
65	Preference 1	1		1 = The product from the first test period		PREFERENC E_1

	Field Name	Data Type	Units	Values	Pre-Filled Values	Include Field OID
				2 = The product from the second test period		
67	Preference no. 1	1				MULTI_PREF_1
68	Preference no. 2	1				MULTI_PREF_2
69	Preference no. 3	1				MULTI_PREF_3
70	Preference no. 4	1				MULTI_PREF_4
71	Preference no. 5	1				MULTI_PREF_5
72	Preference no. 6	1				MULTI_PREF_6
73	Preference no. 7	1				MULTI_PREF_7
74	Preference no. 8	1				MULTI_PREF_8

Production Germany 14. Jan. 2020: Primary matrix
Folder: Final evaluation TP2
Form: Final evaluation TP 2
Generated On: 30 Sep 2020 12:31:31

	Field Name	Data Type	Units	Values	Pre-Filled Values	Include Field OID
75	Preference no. 9	1				MULTI_PREF_9
76	Preference 2	1		1 = The product from the first test period<br>2 = The product from the second test period<br>3 = Your own product		PREFERENCE_2
78	Preference no. 21	1				MULTI_PREF2_1
79	Preference no. 22	1				MULTI_PREF2_2
80	Preference no. 23	1				MULTI_PREF2_3
81	Preference no. 24	1				MULTI_PREF2_4
82	Preference no. 25	1				MULTI_PREF2_5

	Field Name	Data Type	Units	Values	Pre-Filled Values	Include Field OID
83	Preference no. 26	1				MULTI_PREF2_6
84	Preference no. 27	1				MULTI_PREF2_7
85	Preference no. 28	1				MULTI_PREF2_28
86	Preference no. 29	1				MULTI_PREF2_9

Production Germany 14. Jan. 2020: Primary matrix
Folder: Measurements
Form: Skin discolouration
Generated On: 30 Sep 2020 12:31:31

STUDY ID	______	1
SITE ID	______	2
SUBJECT ID	______	3
Photo date	______	4
Photo time	______	5
Sequence no.	______	6
PHOTO ID	______	7
Total discoloration area (cm2)	______	8
Discoloration area_Inner zone 3 cm (cm2)	______	9
Discoloration area_Outer zone 3 cm (cm2)	______	10
Total Intensity 0-10% (cm2)	______	11
Total Intensity 11-20% (cm2)	______	12
Total Intensity 21-30% (cm2)	______	13

Total Intensity
31-40% (cm2) ______ 14

Total Intensity
41-50% (cm2) ______ 15

Total Intensity
51-60% (cm2) ______ 16

Total Intensity
61-70% (cm2) ______ 17

Total Intensity
71-80% (cm2) ______ 18

Total Intensity
81-90% (cm2) ______ 19

Total Intensity
91-100% (cm2) ______ 20

Intensity_Inner zone (3 cm)
0-10% (cm2) ______ 21

Intensity_Inner zone (3 cm)
11-20% (cm2) ______ 22

Intensity_Inner zone (3 cm)
21-30% (cm2) ______ 23

Intensity_Inner zone (3 cm)
31-40% (cm2) ______ 24

Production Germany 14. Jan. 2020: Primary matrix
Folder: Measurements
Form: Skin discolouration
Generated On: 30 Sep 2020 12:31:31

Intensity_Inner zone (3 cm)
41-50% (cm2) ______________ 25

Intensity_Inner zone (3 cm)
51-60% (cm2) ______________ 26

Intensity_Inner zone (3 cm)
61-70% (cm2) ______________ 27

Intensity_Inner zone (3 cm)
71-80% (cm2) ______________ 28

Intensity_Inner zone (3 cm)
81-90% (cm2) ______________ 29

Intensity_Inner zone (3 cm)
91-100% (cm2) ______________ 30

Intensity_outer zone (3 cm)
0-10% (cm2) ______________ 31

Intensity_outer zone (3 cm)
11-20% (cm2) ______________ 32

Intensity_outer zone (3 cm)
21-30% (cm2) ______________ 33

Intensity_outer zone (3 cm)
31-40% (cm2) ______________ 34

Intensity_outer zone (3 cm)
41-50% (cm2) ______________ 35

Intensity_outer zone (3 cm) 51-60% (cm2) ______ 36

Intensity_outer zone (3 cm) 61-70% (cm2) ______ 37

Intensity_outer zone (3 cm) 71-80% (cm2) ______ 38

Intensity_outer zone (3 cm) 81-90% (cm2) ______ 39

Intensity_outer zone (3 cm) 91-100% (cm2) ______ 40

Production Germany 14. Jan. 2020: Primary matrix
Folder: Measurements
Form: Skin discolouration
Generated On: 30 Sep 2020 12:31:31

	Field Name	Data Type	Units	Values	Pre-Filled Values	Include Field OID
1	STUDY ID	$50				STUDY_ID
2	SITE ID	$50				SITE_ID
3	SUBJECT ID	$5				SUBJECT_ID
4	Photo date	dd MMM yyyy				PHOTODAT
5	Photo time	$12				PHOTOTM
6	Sequence no.	5				SEQUENCE
7	PHOTO ID	$70				PHOTO_ID
8	Total discolouration	3.3				DISCOLOURATION_TOTAL
9	Inner discolouration	3.3				DISCOLOURATION_INNER
10	Outer discolouration	3.3				DISCOLOURATION_OUTER

	Field Name	Data Type	Units	Values	Pre-Filled Values	Include Field OID
11	Total intensity 0	3.3				INTENSITY _TOTAL_0
12	Total intensity 1	3.3				INTENSITY _TOTAL_1
13	Total intensity 2	3.3				INTENSITY _TOTAL_2
14	Total intensity 3	3.3				INTENSITY _TOTAL_3
15	Total intensity 4	3.3				INTENSITY _TOTAL_4
16	Total intensity 5	3.3				INTENSITY _TOTAL_5
17	Total intensity 6	3.3				INTENSITY _TOTAL_6
18	Total intensity 7	3.3				INTENSITY _TOTAL_7
19	Total intensity 8	3.3				INTENSITY _TOTAL_8
20	Total intensity 9	3.3				INTENSITY _TOTAL_9

Production Germany 14. Jan. 2020: Primary matrix
Folder: Measurements
Form: Skin discolouration
Generated On: 30 Sep 2020 12:31:31

	Field Name	Data Type	Units	Values	Pre-Filled Values	Include Field OID
21	Inner intensity 0	3.3				INTENSITY_INNER_0
22	Inner intensity 1	3.3				INTENSITY_INNER_1
23	Inner intensity 2	3.3				INTENSITY_INNER_2
24	Inner intensity 3	3.3				INTENSITY_INNER_3
25	Inner intensity 4	3.3				INTENSITY_INNER_4
26	Inner intensity 5	3.3				INTENSITY_INNER_5
27	Inner intensity 6	3.3				INTENSITY_INNER_6
28	Inner intensity 7	3.3				INTENSITY_INNER_7
29	Inner intensity 8	3.3				INTENSITY_INNER_8
30	Inner intensity 9	3.3				INTENSITY_INNER_9

Production Germany 14. Jan. 2020: Primary matrix
Folder: Measurements
Form: Skin discolouration
Generated On: 30 Sep 2020 12:31:31

	Field Name	Data Type	Units	Values	Pre-Filled Values	Include Field OID
31	Outer intensity 0	3.3				INTENSITY _OUTER_0
32	Outer intensity 1	3.3				INTENSITY _OUTER_1
33	Outer intensity 2	3.3				INTENSITY _OUTER_2
34	Outer intensity 3	3.3				INTENSITY _OUTER_3
35	Outer intensity 4	3.3				INTENSITY _OUTER_4
36	Outer intensity 5	3.3				INTENSITY _OUTER_5
37	Outer intensity 6	3.3				INTENSITY _OUTER_6
38	Outer intensity 7	3.3				INTENSITY _OUTER_7
39	Outer intensity 8	3.3				INTENSITY _OUTER_8
40	Outer intensity 9	3.3				INTENSITY _OUTER_9

Production Germany 14. Jan. 2020: Primary matrix
Folder: Measurements
Form: Leakage measurements
Generated On: 30 Sep 2020 12:31:31

STUDY ID	____________	1
SITE ID	____________	2
SUBJECT ID	____________	3
Photo date	____________	4
Photo time	____________	5
Sequence no.	____________	6
PHOTO ID	____________	7
Leakage area (cm2)	____________	8
Shortest distance to edge (cm)	____________	9
Area center hole	____________	10
Area baseplate	____________	11

Production Germany 14. Jan. 2020: Primary matrix
Folder: Measurements
Form: Leakage measurements
Generated On: 30 Sep 2020 12:31:31

	Field Name	Data Type	Units	Values	Pre-Filled Values	Include Field OID
1	STUDY ID	$50				STUDY_ID
2	SITE ID	$50				SITE_ID
3	SUBJECT ID	$5				SUBJECT_ID
4	Photo date	dd MMM yyyy				PHOTODAT
5	Photo time	$12				PHOTOTM
6	Sequence no.	5				SEQUENCE
7	PHOTO ID	$70				PHOTO_ID
8	Area of leakage	3.3				AREA_LEAKAGE
9	Shortest distance	3.3				DISTANCE
10	Area of center hole	3.3				AREA_CENTERHOLE
11	Area of baseplate	3.3				AREA_BASEPLATE

Production Germany 14. Jan. 2020: Primary matrix
Folder: Measurements >> DET score CA visit 1
Form: DET score by external nurse
Generated On: 30 Sep 2020 12:31:31

Please evaluate the skin on the photo using the DET score

Domain 1: Discolouration

Area of discolouration (3)

0 ☐
1 ☐
2 ☐
3 ☐

Severity of discolouration (4)

1 ☐
2 ☐

Domain 2: Erosion

Area of erosion (6)

0 ☐
1 ☐
2 ☐
3 ☐

Severity of erosion (7)

1 ☐
2 ☐

Domain 3: Tissue overgrowth

Area of tissue overgrowth (9)

0 ☐
1 ☐
2 ☐
3 ☐

Production Germany 14. Jan. 2020: Primary matrix
Folder: Measurements >> DET score CA visit 1
Form: DET score by external nurse
Generated On: 30 Sep 2020 12:31:31

Severity of tissue overgrowth 1 ☐ 10
2 ☐

Total DET score ____________ 11

	Field Name	Data Type	Units	Values	Pre-Filled Values	Include Field OID
3	Area of discolouration	1		0 = 0<br>1 = 1<br>2 = 2<br>3 = 3		DISCOLOUR_AREA
4	Severity of discolouration	1		1 = 1<br>2 = 2		DISCOLOUR_SEVERITY
6	Area of erosion	1		0 = 0<br>1 = 1<br>2 = 2<br>3 = 3		EROSION_AREA
7	Severity of erosion	1		1 = 1<br>2 = 2		EROSION_SEVERITY
9	Area of tissue overgrowth	1		0 = 0<br>1 = 1<br>2 = 2<br>3 = 3		OVERGROWTH_AREA
10	Severity of tissue overgrowth	1		1 = 1<br>2 = 2		OVERGROWTH_SEVERITY
11	Total DET score	2				DETSCORE_TOTAL

Please evaluate the skin on the photo using the DET score

Domain 1: Discolouration

Area of discolouration	0 ▢ (3)<br>1 ▢<br>2 ▢<br>3 ▢
Severity of discolouration	1 ▢ (4)<br>2 ▢

Domain 2: Erosion

Area of erosion	0 ▢ (6)<br>1 ▢<br>2 ▢<br>3 ▢
Severity of erosion	1 ▢ (7)<br>2 ▢

Domain 3: Tissue overgrowth

Area of tissue overgrowth	0 ▢ (9)<br>1 ▢<br>2 ▢<br>3 ▢

Severity of tissue overgrowth — 1 ☐ 2 ☐ (10)

Total DET score ________ (11)

Production Germany 14. Jan. 2020: Primary matrix
Folder: Measurements >> DET score CA visit 2
Form: DET score by external nurse
Generated On: 30 Sep 2020 12:31:31

	Field Name	Data Type	Units	Values	Pre-Filled Values	Include Field OID
3	Area of discolouration	1		0 = 0<br>1 = 1<br>2 = 2<br>3 = 3		DISCOLOUR_AREA
4	Severity of discolouration	1		1 = 1<br>2 = 2		DISCOLOUR_SEVERITY
6	Area of erosion	1		0 = 0<br>1 = 1<br>2 = 2<br>3 = 3		EROSION_AREA
7	Severity of erosion	1		1 = 1<br>2 = 2		EROSION_SEVERITY
9	Area of tissue overgrowth	1		0 = 0<br>1 = 1<br>2 = 2<br>3 = 3		OVERGROWTH_AREA
10	Severity of tissue overgrowth	1		1 = 1<br>2 = 2		OVERGROWTH_SEVERITY
11	Total DET score	2				DETSCORE_TOTAL

Please evaluate the skin on the photo using the DET score

Domain 1: Discolouration

Area of discolouration	0 ☐ (3)<br>1 ☐<br>2 ☐<br>3 ☐
Severity of discolouration	1 ☐ (4)<br>2 ☐

Domain 2: Erosion

Area of erosion	0 ☐ (6)<br>1 ☐<br>2 ☐<br>3 ☐
Severity of erosion	1 ☐ (7)<br>2 ☐

Domain 3: Tissue overgrowth

Area of tissue overgrowth	0 ☐ (9)<br>1 ☐<br>2 ☐<br>3 ☐

Production Germany 14. Jan. 2020: Primary matrix
Folder: Measurements >> DET score CA visit 3
Form: DET score by external nurse
Generated On: 30 Sep 2020 12:31:31

Severity of tissue overgrowth 1 ☐ (10)
2 ☐

Total DET score ________ (11)

Production Germany 14. Jan. 2020: Primary matrix
Folder: Measurements >> DET score CA visit 3
Form: DET score by external nurse
Generated On: 30 Sep 2020 12:31:31

	Field Name	Data Type	Units	Values	Pre-Filled Values	Include Field OID
3	Area of discolouration	1		0 = 0<br>1 = 1<br>2 = 2<br>3 = 3		DISCOLOUR_AREA
4	Severity of discolouration	1		1 = 1<br>2 = 2		DISCOLOUR_SEVERITY
6	Area of erosion	1		0 = 0<br>1 = 1<br>2 = 2<br>3 = 3		EROSION_AREA
7	Severity of erosion	1		1 = 1<br>2 = 2		EROSION_SEVERITY
9	Area of tissue overgrowth	1		0 = 0<br>1 = 1<br>2 = 2<br>3 = 3		OVERGROWTH_AREA
10	Severity of tissue overgrowth	1		1 = 1<br>2 = 2		OVERGROWTH_SEVERITY
11	Total DET score	2				DETSCORE_TOTAL

Please evaluate the skin on the photo using the DET score

Domain 1: Discolouration

Area of discolouration (3)

- 0 ☐
- 1 ☐
- 2 ☐
- 3 ☐

Severity of discolouration (4)

- 1 ☐
- 2 ☐

Domain 2: Erosion

Area of erosion (6)

- 0 ☐
- 1 ☐
- 2 ☐
- 3 ☐

Severity of erosion (7)

- 1 ☐
- 2 ☐

Domain 3: Tissue overgrowth

Area of tissue overgrowth (9)

- 0 ☐
- 1 ☐
- 2 ☐
- 3 ☐

Severity of tissue overgrowth	1 ▢ (10)<br>2 ▢
Total DET score	______ (11)

Production Germany 14. Jan. 2020: Primary matrix
Folder: Measurements >> DET score DK visit 1
Form: DET score by external nurse
Generated On: 30 Sep 2020 12:31:31

	Field Name	Data Type	Units	Values	Pre-Filled Values	Include Field OID
3	Area of discolouration	1		0 = 0<br>1 = 1<br>2 = 2<br>3 = 3		DISCOLOUR_AREA
4	Severity of discolouration	1		1 = 1<br>2 = 2		DISCOLOUR_SEVERITY
6	Area of erosion	1		0 = 0<br>1 = 1<br>2 = 2<br>3 = 3		EROSION_AREA
7	Severity of erosion	1		1 = 1<br>2 = 2		EROSION_SEVERITY
9	Area of tissue overgrowth	1		0 = 0<br>1 = 1<br>2 = 2<br>3 = 3		OVERGROWTH_AREA
10	Severity of tissue overgrowth	1		1 = 1<br>2 = 2		OVERGROWTH_SEVERITY
11	Total DET score	2				DETSCORE_TOTAL

Production Germany 14. Jan. 2020: Primary matrix
Folder: Measurements >> DET score DK visit 2
Form: DET score by external nurse
Generated On: 30 Sep 2020 12:31:31

Please evaluate the skin on the photo using the DET score

Domain 1: Discolouration

Area of discolouration (3)

0 ☐
1 ☐
2 ☐
3 ☐

Severity of discolouration (4)

1 ☐
2 ☐

Domain 2: Erosion

Area of erosion (6)

0 ☐
1 ☐
2 ☐
3 ☐

Severity of erosion (7)

1 ☐
2 ☐

Domain 3: Tissue overgrowth

Area of tissue overgrowth (9)

0 ☐
1 ☐
2 ☐
3 ☐

Severity of tissue overgrowth	1 ☐ (10)<br>2 ☐
Total DET score	________ (11)

Production Germany 14. Jan. 2020: Primary matrix
Folder: Measurements >> DET score DK visit 2
Form: DET score by external nurse
Generated On: 30 Sep 2020 12:31:31

	Field Name	Data Type	Units	Values	Pre-Filled Values	Include	Field OID
3	Area of discolouration	1		0 = 0<br>1 = 1<br>2 = 2<br>3 = 3			DISCOLOUR_AREA
4	Severity of discolouration	1		1 = 1<br>2 = 2			DISCOLOUR_SEVERITY
6	Area of erosion	1		0 = 0<br>1 = 1<br>2 = 2<br>3 = 3			EROSION_AREA
7	Severity of erosion	1		1 = 1<br>2 = 2			EROSION_SEVERITY
9	Area of tissue overgrowth	1		0 = 0<br>1 = 1<br>2 = 2<br>3 = 3			OVERGROWTH_AREA
10	Severity of tissue overgrowth	1		1 = 1<br>2 = 2			OVERGROWTH_SEVERITY
11	Total DET score	2					DETSCORE_TOTAL

Please evaluate the skin on the photo using the DET score

Domain 1: Discolouration

Area of discolouration **(3)**

- 0 ☐
- 1 ☐
- 2 ☐
- 3 ☐

Severity of discolouration **(4)**

- 1 ☐
- 2 ☐

Domain 2: Erosion

Area of erosion **(6)**

- 0 ☐
- 1 ☐
- 2 ☐
- 3 ☐

Severity of erosion **(7)**

- 1 ☐
- 2 ☐

Domain 3: Tissue overgrowth

Area of tissue overgrowth **(9)**

- 0 ☐
- 1 ☐
- 2 ☐
- 3 ☐

Production Germany 14. Jan. 2020: Primary matrix
Folder: Measurements >> DET score DK visit 3
Form: DET score by external nurse
Generated On: 30 Sep 2020 12:31:31

Severity of tissue overgrowth 1 ▢ (10)
2 ▢

Total DET score ____________ (11)

Production Germany 14. Jan. 2020: Primary matrix
Folder: Measurements >> DET score DK visit 3
Form: DET score by external nurse
Generated On: 30 Sep 2020 12:31:31

	Field Name	Data Type	Units	Values	Pre-Filled Values	Include Field OID
3	Area of discolouration	1		0 = 0<br>1 = 1<br>2 = 2<br>3 = 3		DISCOLOUR_AREA
4	Severity of discolouration	1		1 = 1<br>2 = 2		DISCOLOUR_SEVERITY
6	Area of erosion	1		0 = 0<br>1 = 1<br>2 = 2<br>3 = 3		EROSION_AREA
7	Severity of erosion	1		1 = 1<br>2 = 2		EROSION_SEVERITY
9	Area of tissue overgrowth	1		0 = 0<br>1 = 1<br>2 = 2<br>3 = 3		OVERGROWTH_AREA
10	Severity of tissue overgrowth	1		1 = 1<br>2 = 2		OVERGROWTH_SEVERITY
11	Total DET score	2				DETSCORE_TOTAL

Please evaluate the skin on the photo using the DET score

Domain 1: Discolouration

Area of discolouration (3)

0 ▢
1 ▢
2 ▢
3 ▢

Severity of discolouration (4)

1 ▢
2 ▢

Domain 2: Erosion

Area of erosion (6)

0 ▢
1 ▢
2 ▢
3 ▢

Severity of erosion (7)

1 ▢
2 ▢

Domain 3: Tissue overgrowth

Area of tissue overgrowth (9)

0 ▢
1 ▢
2 ▢
3 ▢

Severity of tissue overgrowth	1 ▢ (10)<br>2 ▢
Total DET score	______________ (11)

Production Germany 14. Jan. 2020: Primary matrix
Folder: Measurements >> DET score final visit 1
Form: DET score by external nurse
Generated On: 30 Sep 2020 12:31:31

	Field Name	Data Type	Units	Values	Pre-Filled Values	Include Field OID
3	Area of discolouration	1		0 = 0<br>1 = 1<br>2 = 2<br>3 = 3		DISCOLOUR_AREA
4	Severity of discolouration	1		1 = 1<br>2 = 2		DISCOLOUR_SEVERITY
6	Area of erosion	1		0 = 0<br>1 = 1<br>2 = 2<br>3 = 3		EROSION_AREA
7	Severity of erosion	1		1 = 1<br>2 = 2		EROSION_SEVERITY
9	Area of tissue overgrowth	1		0 = 0<br>1 = 1<br>2 = 2<br>3 = 3		OVERGROWTH_AREA
10	Severity of tissue overgrowth	1		1 = 1<br>2 = 2		OVERGROWTH_SEVERITY
11	Total DET score	2				DETSCORE_TOTAL

Production Germany 14. Jan. 2020: Primary matrix
Folder: Measurements >> DET score final visit 2
Form: DET score by external nurse
Generated On: 30 Sep 2020 12:31:31

Please evaluate the skin on the photo using the DET score

Domain 1: Discolouration

Area of discolouration (3)

0 ▢
1 ▢
2 ▢
3 ▢

Severity of discolouration (4)

1 ▢
2 ▢

Domain 2: Erosion

Area of erosion (6)

0 ▢
1 ▢
2 ▢
3 ▢

Severity of erosion (7)

1 ▢
2 ▢

Domain 3: Tissue overgrowth

Area of tissue overgrowth (9)

0 ▢
1 ▢
2 ▢
3 ▢

Severity of tissue overgrowth	1 ▢ (10)<br>2 ▢
Total DET score	________ (11)

Production Germany 14. Jan. 2020: Primary matrix
Folder: Measurements >> DET score final visit 2
Form: DET score by external nurse
Generated On: 30 Sep 2020 12:31:31

	Field Name	Data Type	Units	Values	Pre-Filled Values	Include Field OID
3	Area of discolourati on	1		0 = 0<br>1 = 1<br>2 = 2<br>3 = 3		DISCOLOUR _AREA
4	Severity of discolourati on	1		1 = 1<br>2 = 2		DISCOLOUR _SEVERITY
6	Area of erosion	1		0 = 0<br>1 = 1<br>2 = 2<br>3 = 3		EROSION_A REA
7	Severity of erosion	1		1 = 1<br>2 = 2		EROSION_S EVERITY
9	Area of tissue overgrowth	1		0 = 0<br>1 = 1<br>2 = 2<br>3 = 3		OVERGROW TH_AREA
10	Severity of tissue overgrowth	1		1 = 1<br>2 = 2		OVERGROW TH_SEVERI TY
11	Total DET score	2				DETSCORE_ TOTAL

Production Germany 14. Jan. 2020: Primary matrix
Folder: Measurements >> DET score final visit 3
Form: DET score by external nurse
Generated On: 30 Sep 2020 12:31:31

Please evaluate the skin on the photo using the DET score

Domain 1: Discolouration

Area of discolouration (3)

0 ▢
1 ▢
2 ▢
3 ▢

Severity of discolouration (4)

1 ▢
2 ▢

Domain 2: Erosion

Area of erosion (6)

0 ▢
1 ▢
2 ▢
3 ▢

Severity of erosion (7)

1 ▢
2 ▢

Domain 3: Tissue overgrowth

Area of tissue overgrowth (9)

0 ▢
1 ▢
2 ▢
3 ▢

Severity of tissue overgrowth — 1 ▢ 2 ▢ (10)

Total DET score ________ (11)

Production Germany 14. Jan. 2020: Primary matrix
Folder: Measurements >> DET score final visit 3
Form: DET score by external nurse
Generated On: 30 Sep 2020 12:31:31

	Field Name	Data Type	Units	Values	Pre-Filled Values	Include Field OID
3	Area of discolouration	1		0 = 0<br>1 = 1<br>2 = 2<br>3 = 3		DISCOLOUR_AREA
4	Severity of discolouration	1		1 = 1<br>2 = 2		DISCOLOUR_SEVERITY
6	Area of erosion	1		0 = 0<br>1 = 1<br>2 = 2<br>3 = 3		EROSION_AREA
7	Severity of erosion	1		1 = 1<br>2 = 2		EROSION_SEVERITY
9	Area of tissue overgrowth	1		0 = 0<br>1 = 1<br>2 = 2<br>3 = 3		OVERGROWTH_AREA
10	Severity of tissue overgrowth	1		1 = 1<br>2 = 2		OVERGROWTH_SEVERITY
11	Total DET score	2				DETSCORE_TOTAL

Production Germany 14. Jan. 2020: Primary matrix
Folder: Administration
Form: DM Administration
Generated On: 30 Sep 2020 12:31:31

Analysis population PP	No ☐ Yes ☐ (1)
Analysis population ITT	No ☐ Yes ☐ (2)
Analysis population Safety	No ☐ Yes ☐ (3)
Analysis population Screen Failure	No ☐ Yes ☐ (4)

Production Germany 14. Jan. 2020: Primary matrix
Folder: Administration
Form: DM Administration
Generated On: 30 Sep 2020 12:31:31

	Field Name	Data Type	Units	Values	Pre-Filled Values	Include Field OID
1	Analysis population Per Protocol	1		0 = No<br>1 = Yes		POPULATION_PP
2	Analysis population ITT	1		0 = No<br>1 = Yes		POPULATION_ITT
3	Analysis population Safety	1		0 = No<br>1 = Yes		POPULATION_SAFETY
4	Analysis population Screen Failure	1		0 = No<br>1 = Yes		POPULATION_SCREEN_FAILURE
